# Quantitative Pharmacokinetics Reveal Impact of Lipid Composition on Microbubble and Nanoprogeny Shell Fate

**DOI:** 10.1002/advs.202304453

**Published:** 2023-11-30

**Authors:** Maneesha A. Rajora, Alexander Dhaliwal, Mark Zheng, Victor Choi, Marta Overchuk, Jenny W. H. Lou, Carly Pellow, David Goertz, Juan Chen, Gang Zheng

**Affiliations:** ^1^ Princess Margaret Cancer Centre University Health Network Toronto Ontario M5G 1L7 Canada; ^2^ Institute of Biomedical Engineering University of Toronto Toronto Ontario M5G 1L7 Canada; ^3^ Department of Medical Biophysics University of Toronto Toronto Ontario M5G 1L7 Canada; ^4^ Joint Department of Biomedical Engineering University of North Carolina at Chapel Hill and North Carolina State University Chapel Hill NC 27599 USA; ^5^ Sunnybrook Research Institute Toronto Ontario M4N 3M5 Canada

**Keywords:** drug‐delivery, focused ultrasound, Microbubbles, Pharmacokinetics, radiolabeling

## Abstract

Microbubble‐enabled focused ultrasound (MB‐FUS) has revolutionized nano and molecular drug delivery capabilities. Yet, the absence of longitudinal, systematic, quantitative studies of microbubble shell pharmacokinetics hinders progress within the MB‐FUS field. Microbubble radiolabeling challenges contribute to this void. This barrier is overcome by developing a one‐pot, purification‐free copper chelation protocol able to stably radiolabel diverse porphyrin‐lipid‐containing Definity® analogues (pDefs) with >95% efficiency while maintaining microbubble physicochemical properties. Five tri‐modal (ultrasound‐, positron emission tomography (PET)‐, and fluorescent‐active) **[** ^64^Cu**]**Cu‐pDefs are created with varying lipid acyl chain length and charge, representing the most prevalently studied microbubble compositions. In vitro, C16 chain length microbubbles yield 2–3x smaller nanoprogeny than C18 microbubbles post FUS. In vivo, **[** ^64^Cu**]**Cu‐pDefs are tracked in healthy and 4T1 tumor‐bearing mice ± FUS over 48 h qualitatively through fluorescence imaging (to characterize particle disruption) and quantitatively through PET and γ‐counting. These studies reveal the impact of microbubble composition and FUS on microbubble dissolution rates, shell circulation, off‐target tissue retention (predominantly the liver and spleen), and FUS enhancement of tumor delivery. These findings yield pharmacokinetic microbubble structure‐activity relationships that disrupt conventional knowledge, the implications of which on MB‐FUS platform design, safety, and nanomedicine delivery are discussed.

## Graphical abstract

Development and application of a new microbubble radiolabeling protocol, yielding multimodal (ultrasound, fluorescence and positron emission tomography active) porphyrin Definity analogues (pDefs) with differing acyl chain lengths and charge. These pDefs are applied +/− focused ultrasound to healthy and 4T1 tumor‐bearing mice. Quantitative and semi‐quantitative pharmacokinetic profiles are obtained, from which initial structure‐activity relationships (microbubble dissolution, clearance, and tissue uptake) are created.

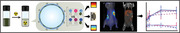

## 1 Introduction

Supramolecular chemistry has expanded cancer imaging and drug delivery capabilities beyond that achievable by small molecules alone. This capacity has been most prevalently explored through nano‐sized supramolecular constructs to improve drug targeting, control bioavailability, and reduce systemic toxicity.^[^ ^1^ ^]^ Yet, these nano systems encounter clinical delivery challenges across intact vasculature and other heterogenous tumor microenvironment barriers, particularly when designed to rely upon passive, non‐universal targeting strategies such as the tumor enhanced permeability and retention (EPR) effect and cancer biomarkers.^[^ ^2^ ^]^ Microbubbles are a relatively new and particularly radical addition to the supramolecular drug delivery family that can overcome these barriers and broaden the therapeutic utility of nano and molecular agents.

As their name implies, microbubbles are micron‐sized agents with a gas core encapsulated typically by a lipid shell that slows core gas dispersion into surrounding biological milieus. The resulting stabilized gas nuclei generate strong ultrasound contrast against surrounding tissue.^[^ ^3^ ^]^ Combination with focused ultrasound (FUS) transformed lipid‐shelled microbubbles from diagnostic agents into revolutionary therapeutic platforms and drug delivery vehicles in the early 2000s.^[^ ^4^ ^]^ In the presence of image‐guided FUS, microbubbles could now be stimulated focally in deep tissues to produce tunable bio‐effects ranging from transient vasculature permeabilization to localized tissue ablation with millimeter precision.^[^ ^5^, ^6^, ^7^, ^8^, ^9^, ^10^, ^11^, ^12^, ^13^ ^]^ This opened uncharted avenues for mechanical modulation of tumor microenvironment barriers to actively deliver nanomedicines and molecular drugs across otherwise impermeable vasculature in a targeted, safe, and minimally invasive manner, notably unreliant on passive EPR and biomarker delivery strategies.^[^ ^9^ ^]^ These unique characteristics led to the rapid expansion of microbubble therapeutic applications in the last two decades (Figure S1A, Supporting Information) and clinical trials of microbubble‐enabled FUS (MB‐FUS) in patients with neurological disorders^[^ ^14^, ^15^ ^]^ and brain, pancreatic, breast, and liver cancers.^[^ ^16^, ^17^, ^18^, ^19^, ^20^, ^21^ ^]^ Though initially studied to improve the delivery and therapeutic efficacy of co‐administered nanomedicines and molecular agents, MB‐FUS has been increasingly explored as an all‐in‐one therapeutic platform (Figure S1B, Supporting Information). Here, nanoparticles and molecular agents are tethered directly onto the microbubble shell, which when irradiated by FUS, undergoes an in situ fragmentation into nano‐sized progeny for localized drug delivery.^[^ ^22^, ^23^ ^]^ Collectively, microbubbles present a promising arsenal of nanomedicine delivery possibilities that exceed the therapeutic diversity and capacity of conventional nano‐sized supramolecular platforms alone.

Despite these advances, the therapeutic study of lipid microbubbles falters compared to their nano‐sized supramolecular counterparts in one key domain: pharmacokinetic study. This tenant of drug delivery vehicle development^[^ ^24^ ^]^ has largely been overlooked in the MB‐FUS field due to the initial application of lipid microbubbles as ultrasound contrast agents. In this context, lipid microbubbles were thought to remain confined to vasculature^[^ ^25^, ^26^, ^27^ ^]^ for the short durations (minutes‐long) in which microbubbles retained their high‐contrast, gas‐filled core. As such, microbubble pharmacokinetic characterization focused on examining microbubble core gas dissolution and gas clearance.^[^ ^28^, ^29^ ^]^ Any limited examination of lipid shell fate was restricted to a one‐hour post‐injection timeframe in the absence of therapeutic FUS.^[^ ^30^, ^31^, ^32^ ^]^ In fact, Definity, North America's most widely used microbubble for clinical imaging, achieved regulatory status in 2001^[^ ^33^ ^]^ without published dissemination of any pharmacokinetic analysis of its lipid shell. Though passable for initial ultrasonography applications, this void in longitudinal lipid microbubble shell fate characterization is no longer acceptable. In the therapeutic domain, microbubble shells should not be viewed as benign, blood‐restricted entities. These shells have been shown to be capable of extravasation beyond vascular boundaries into sonicated tissue.^[^ ^23^, ^34^ ^]^ This on (and off) target shell deposition has safety and efficacy repercussions with growing discussions around dose‐limiting sterile inflammation responses to conventional MB‐FUS^[^ ^35^, ^36^, ^37^ ^]^ and more broadly around lipid agent hypersensitivity reactions.^[^ ^38^, ^39^, ^40^ ^]^ Recent mechanistic studies suggest that the extent of such shell transfer also impacts MB‐FUS efficacy in a microbubble composition‐dependent manner.^[^ ^41^, ^42^ ^]^ Thus, systematic pharmacokinetic profiling of microbubbles of differing compositions in the presence and absence of FUS application is needed to inform and enhance MB‐FUS safety and drug delivery efficacy using mechanism‐based platform design.

Such pharmacokinetic structure‐activity relationships directly govern the evolution of all‐in‐one theranostic microbubbles, whose growth is overtaking conventional co‐delivery MB‐FUS paradigms preclinically, comprising ≈40% of all therapeutic lipid microbubble studies (Figure S1B, Supporting Information). Pharmacokinetic profiling that considers the impacts of FUS and microbubble chemistry can illuminate how to control systemic exposure and off‐target organ toxicity of drug‐conjugated microbubble shells. The power of such pharmacokinetics‐driven intentional design is well‐illustrated in the nanomedicine field, leading to clinical successes like Onpattro and Doxil.^[^ ^43^ ^]^ It could be similarly applied to quantify and maximize on‐target drug delivery efficacy of these increasingly explored all‐in‐one microbubble platforms and delineate their utility versus other nanoparticle and molecular drug delivery platforms. To this end, microbubble shell pharmacokinetic analysis +/− FUS is needed to understand how the nanoprogeny^[^ ^22^, ^23^, ^44^, ^45^, ^46^ ^]^ of microbubbles behave in vivo and thereby determine the strengths of the micro‐to‐nano conversion as a nanomedicine delivery strategy, defining these findings in relation to the existing repertoire of nanomedicine pharmacokinetic knowledge. Collectively, these insights highlight the need for quantitative pharmacokinetic structure‐activity profiling of microbubbles to advance the safety, efficacy, and mechanistic understanding of co‐delivery and all‐in‐one MB‐FUS platforms alike.

Despite their recognized need, there is a continued absence of studies that holistically evaluate lipid microbubble shell clearance, kinetic biodistribution, or FUS and microbubble composition impacts thereof over the requisite 0–48 h timeframes typically assayed for lipid drug delivery agents^[^ ^47^ ^]^ (Figure S1A, Supporting Information). Radioisotope labeling and tracing remains the gold standard for characterizing drug system pharmacokinetic profiles.^[^ ^48^, ^49^ ^]^ However, microbubble radiolabeling remains a non‐trivial barrier to obtaining robust pharmacokinetic structure‐activity relationships. Current microbubble radiolabeling techniques require reaction and purification protocols that can disturb the delicate balance of temperature, pH, salt concentration, and physical stability required to synthesize stable particles with controlled physicochemical properties,^[^ ^50^ ^–^ ^55^ ^]^ intrinsic to MB‐FUS bioeffects.^[^ ^56^, ^57^ ^]^ Furthermore, protocols to‐date employed radioisotopes such as ^18^F (t_1/2_ 1.8 h),^[^ ^30^, ^31^ ^] 99m^Tc (t_1/2_ 6 h),^[^ ^32^, ^58^ ^]^ and ^68^Ga (t_1/2_ 1 h)^[^ ^59^ ^]^ with half‐lives too short to capture the complete 0–48 h window typically studied for lipid supramolecular drug delivery vehicle in vivo pharmacokinetics. As alternatives to microbubble radiolabeling and tracking, some authors use microbubble dissolution, microparticle proxies, and fluorescence imaging to study microbubble pharmacokinetics, which yield unrepresentative or semi‐quantitative data.^[^ ^60^, ^61^ ^]^ Collectively, these obstacles culminate into incomplete, non‐quantitative pharmacokinetic assessments that do not accurately recapitulate clinical and preclinical formulations. To overcome the void of knowledge surrounding microbubble pharmacokinetic structure‐activity relationships, a novel microbubble radiolabeling strategy is needed that efficiently and stably incorporates longer‐living radioisotopes into diverse formulations while conserving microbubble physicochemical properties.

**Figure 1:**
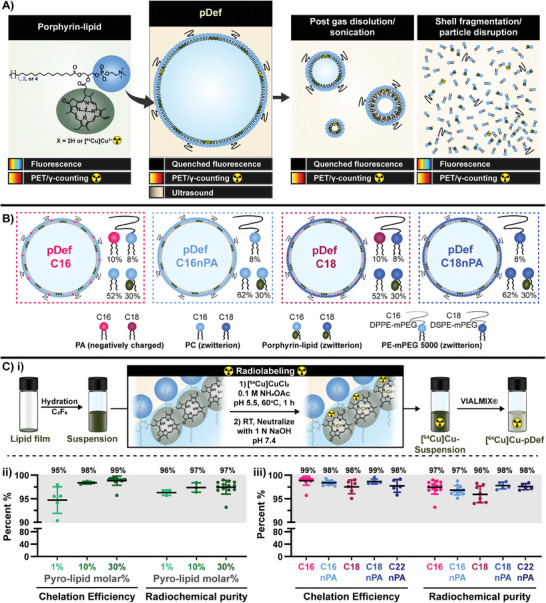
Multimodal (pDef) microbubbles were created to systematically quantify microbubble pharmacokinetics. A) Porphyrin‐lipid imparts pDefs with trimodal, state‐sensitive tracking, enabling qualitative fluorescence and quantitative PET/γ‐counting in the disrupted state and ultrasound contrast, quenched fluorescence, and unaffected PET/γ‐counting in the intact microbubble state. B) Four different microbubbles representing clinically and preclinically significant formulation variants of Definity were constructed by modifying chain length (C16 or C18) and shell charge (nPA: absence of phosphatidic acid, PA: phosphatidic acid inclusive). C) (i) A one‐pot, Cu‐chelation procedure was developed that achieved high chelation efficiency and radio‐chemical purity at varied porphyrin‐lipid inclusion fractions (ii) and for all pDef formulations (iii).

In this study, we develop such a microbubble radiolabeling technique and use it to conduct the first longitudinal, multimodal pharmacokinetic study of lipid microbubble and nanoprogeny shell fate in healthy and tumor‐bearing mice +/− FUS. This was enabled by the inclusion of multimodal porphyrin‐lipid moieties into Definity microbubble analogues (pDefs; **Figure** **1**A,B) with systematically modulated compositions (C16 and C18 lipid chain lengths, neutral or anionic charge). Porphyrins are organic, heterocyclic macrocycles with a highly conjugated central ring that can efficiently chelate a variety of metals and radioisotopes.^[^ ^62^, ^63^ ^]^ The inclusion of porphyrin‐lipid into supramolecular structures quenches its endogenous fluorescence, which is restored upon particle disruption (Figure 1A).^[^ ^64^ ^]^ We exploit these features to generate radio‐, fluorescent‐ and ultrasound‐active pDefs through a novel one‐pot, purification‐free microbubble radiolabeling protocol that efficiently (>95% chelation) and stably labeled pDefs of varying compositions with Copper‐64 while conserving microbubble physicochemical and photonic properties (Figure 1C). This unique combination of multimodality is previously unreported in a single lipid microbubble agent. This allowed for robust quantitative tracking of the different microbubbles over 48 h using a combination of positron emission tomography (PET) and γ‐counting for quantitation of lipid shell biodistribution; activatable fluorescence imaging for semi‐quantitative evaluation of particle state; and ultrasonography for evaluating microbubble dissolution. Through this first‐of‐its kind pharmacokinetic study, we illustrate the strong impact of microbubble composition on shell fate, confirm and overturn knowledge governing microbubble and nanomedicine fields, and initiate microbubble structure‐activity relations for improving MB‐FUS safety and drug delivery efficacy.

## 2 Results and Discussion

### 2.1 Literature‐Informed Selection of pDef Compositions

In order to maximize the broader relevance of this study, we reviewed the most prevalently used lipid microbubble formulations in preclinical and clinical studies (Figure S1, Supporting Information). Definity was the most prevalently used microbubble in clinical MB‐FUS studies (Figure S1C, Supporting Information), and thus its lipid composition served as the foundation for pDef formulations (Table S1, Supporting Information). Review of preclinical studies revealed that C16 and C18 chain length phospholipids served as the most utilized host lipids. As such, we explored the baseline C16 Definity formulation (pDef C16) and its C18 chain length variant (pDef C18). Lastly, most pre‐clinical non‐commercial lipid microbubble formulations contained no charged groups, while the most explored commercial agents (Definity and Sonovue) contain an anionic phosphatidic acid (PA) group. Thus no‐PA neutral versions of pDef (C16nPA and C18nPA) were also constructed to better represent lipid diversity among pre‐clinical formulations. This yielded four broadly relevant Definity analogues (pDef C16, pDef C18, pDef C16nPA, and pDef C18nPA, Figure 1B) that allowed us to systematically evaluate the impact of chain length and charge on microbubble pharmacokinetics.

To do so, we required stable porphyrin‐lipid incorporations in these formulations. The direct Definity analogue pDef C16 and its C18 variant were used to establish optimal molar substitution of the associated PC host lipids with porphyrin‐lipid. Optimization end goals included retaining Definity's average number size (1.1–3.3 µm), maintaining sufficient microbubble yield (> 1 × 10^9^ MB mL^−1^), achieving uniform and reproducible size profiles, minimizing microbubble populations > 8 µm in size to prevent gas embolus formation in capillaries,^[^ ^67^, ^68^ ^]^ and maximizing porphyrin fluorescence quenching (consistently >95%) to enable the on/off activatable fluorescence imaging advantages of pDefs depicted in Figure 1A. These criteria were achieved with a 30 mol% substitution of the host PC lipid with porphyrin‐lipid for both C16 and C18 pDefs (Figure S2, Supporting Information). The in‐house Definity formulation exhibited similar sizing to previously published results,^[^ ^69^ ^]^ including a similar volume‐weighted mean size (3.64 ± 0.09 µm vs 3.99 µm) and overall yield (10 ± 4 × 10^9^ MB mL^−1^ vs 13.0 × 10^9^ MB mL^−1^). Inclusion of porphyrin into this formulation (C16 pDef) slightly reduced microbubble yield and increased microbubble size, but microbubbles remained within average parameters described above. All pDefs were therefore formulated with 30 mol% porphyrin‐lipid with complete compositions provided in Table S1 (Supporting Information).

### 2.2 Development of One‐Pot Microbubble Radiolabeling Strategy that Conserves Microbubble Physicochemical Properties

We developed a protocol to label pDef porphyrin with an appropriate radioisotope. **[** ^64^Cu**]**Cu^2^ **^+^** was selected due to its 12.7 h decay half‐life and positron emission that could respectively enable longitudinal (minimum 48 h) and quantitative (PET‐enabled) tracking of microbubble lipid shell fate.^[^ ^70^ ^]^ Rapid and stable Cu^2+^ chelation to porphyrin within supramolecular agents can occur at two stages: 1) labeling of the porphyrin building block prior to its introduction into a supramolecular particle (pre‐insertion method),^[^ ^71^ ^]^ or 2) labeling of porphyrin‐lipid already encapsulated in a supramolecular particle (post‐insertion method).^[^ ^62^ ^]^ We opted for a modified post‐insertion approach applied at the lipid film re‐hydration step of a typical lipid microbubble synthesis protocol (Figure S3, Supporting Information). Chelation was conducted after porphyrin‐lipid inclusion into lipid films but before microbubble activation, which circumvented: 1) radio‐imaging timing and safety constraints associated with pre‐insertion (inefficient loss of radioactivity during lengthy lipid building block combination and solvent evaporation that would also pose radioactive contamination concerns) and 2) microbubble destabilization constraints associated with post‐MB activation chelation (time, heat, and pH required for chelation can destabilize microbubbles and change their physicochemical properties). “Cold” non‐radioactive copper was used to optimize lipid suspension labeling and characterize the resulting microbubbles. A 1:100 Cu:porphyrin ratio was used as being representative of the approximate metal:porphyrin ratio that would result from radiolabeling 30 mol% porphyrin‐lipid containing pDefs with **[** ^64^Cu**]**CuCl_2_ for a desired 0.5–0.6 mCi per mouse radioisotope and 1 mg kg^−1^ porphyrin dose for robust PET, γ‐counting, and fluorescence image acquisition up to 48 h post‐injection. Various Cu‐porphyrin chelation parameters (reaction time, reaction temperature, buffer, pH, lipid concentration, salt extraction) were explored to yield a one‐pot Cu‐chelation procedure (Figure 1C‐i) that met the optimization endpoints of achieving >90% chelation efficiency and conserving pDef physicochemical properties. Specifically, pDef lipid films were hydrated in a 0.1 M ammonium acetate (pH 5.5) buffer, to which a small volume (< 5 µL) CuCl_2_ or **[** ^64^Cu**]**CuCl_2_ was added. The resulting lipid suspension was heated at 60 °C to enable chelation. After 1 h, the suspension was allowed to cool to room temperature and then neutralized with a small volume (<10 µL) of 1 N sodium hydroxide. This simple neutralization step was key to the subsequent successful and reproducible activation of the labeled suspension into stable pDefs (acidic labeling conditions were not conducive to stable microbubble formation, nor was the extraction of ammonium acetate buffer which is typically conducted in post‐insertion porphyrin chelation protocols). Furthermore, it allowed for all steps to be completed within a singular vial and with minimal adjustment of lipid and salt concentrations or the microbubble synthesis protocol, making it easily adaptable as a low‐risk protocol for other radiosynthesis facilities. This was desired for conserving pDef properties and recapitulating the as‐is out‐of‐the‐vial usage of Definity and other clinical MBs.

**Figure 2:**
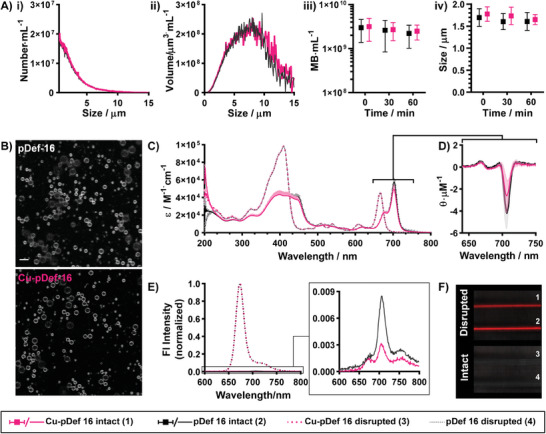
Microbubble physiochemical properties are conserved with Cu chelation. A) C16 pDef Cu‐labeled (pink) and unlabelled (black) average (*n* = 4–7) number‐weighted (i) and volume‐weighted (ii) size distributions and their mean sizes over time (iii, iv) are unaffected by chelation (sizing via Coulter counting, overlapping number distributions). B) Confocal microscopy demonstrates that labeled C16 pDef show comparable microbubble morphology and homogeneity of porphyrin‐lipid distribution throughout the microbubble shell (scale bar = 5 µm). C) Absorbance spectra of intact (PBS) and disrupted (methanol) microbubbles are unchanged by chelation, demonstrating overlapping profiles and indicating similar lipid packing, D) which is confirmed via circular dichroism spectroscopy and the observed negative inflection at the Q‐band associated with porphyrin J‐aggregation (shaded regions for absorbance and CD spectra represent standard deviations). E) Labeled and unlabeled microbubbles both exhibit fluorescence quenching (spectra collected with 410 nm excitation, normalized to disrupted maximum fluorescence intensity) when intact (PBS) and fluorescence recovery upon disruption (via 1% TritonX‐100). Note, when normalized, the fluorescence spectra for disrupted labeled and unlabed pDef overlap, while a small difference is visible for intact fluorescence spectra as can be seen in the insert. F) This fluorescence quenching translates to an on/off indicator of structural state on hyperspectral fluorescence imaging (Maestro II, CRi red filter, 1000 ms exposure, 1 = Cu‐C16 pDef disrupted in 1% TritonX‐100, 2 = unlabeled C16 pDef disrupted in 1% TritonX‐100, 3 = intact Cu‐C16 pDef in PBS, 2 = intact unlabeled C16 pDef in PBS).

This newly developed lipid microbubble radiolabeling protocol was applied across the four rationally designed pDef formulations and three C16 pDef variants of different porphyrin‐lipid molar percentages, all of which achieved 95–99% radiochemical yield and 96–98% radiochemical purity (Figure 1C‐ii,iii; Figure S4, Supporting Information). This high efficiency, high purity labeling circumvented any need for post‐chelation purification unlike previously documented lipid microbubble radiolabeling strategies. Accordingly, this protocol yielded Cu‐labeled pDefs with comparable physicochemical properties as un‐labeled pDefs (**Figure** **2**; Figures S5 and S6, Supporting Information). Using the direct Definity analogue pDef C16 as an example, Figure 2A demonstrates that the Cu‐labelled and unlabelled C16 pDefs had a similar average size distribution (1.8 ± 0.1 µm and 1.7 ± 0.2 µm, respectively), yield (3 ± 1 × 10^9^MB mL^−1^ and 3 ± 2 × 10^9^MB mL^−1^, respectively), and post‐activation stability during typical usage timeframes. This size comparability between Cu‐labeled and unlabeled microbubbles was conserved across the other three pDef formulations (Figure S5 Supporting Information) and with varying porphyrin‐lipid contents (Figure S6, Supporting Information). Confocal microscopy of labeled and unlabeled pDef C16 demonstrated comparable morphology and homogeneous incorporation of porphyrin‐lipid throughout the microbubble shell (Figure 2B). Comparing microbubbles with different chain lengths and charge inclusions, all pDefs achieve sufficient yields (>2 × 10^9^MB mL^−1^) and comparable number‐weighted mean sizes (1.1–1.8 µm). C16 pDefs display a slightly greater volume‐weighted mean size and gas volume than other pDefs. Physicochemical characterization of all four pDef formulations and their chelated variants is summarized in Table S2 (Supporting Information).

The low Cu:porphyrin labeling ratios preserved the photonic properties of pDef. As shown in Figure 2C, labeled and unlabeled intact pDef C16 shells were associated with absorbance spectra akin to those documented for stable incorporation of porphyrin‐lipid into microbubbles.^[^ ^72^ ^]^ Specifically, the intact shells included overlapping red‐shifted Soret and Q‐bands relative to monomeric porphyrin‐lipid. This red‐shift was indicative of ordered aggregation of the porphyrin‐lipid within the pDef shells, confirmed by circular dichroism spectroscopy (Figure 2D), which demonstrated a negative inflection at 705 nm Q‐band for both labeled and unlabeled pDef C16, characteristic of porphyrin J‐aggregation in supramolecular agents. These data indicate that Cu‐chelation did not perturb stable porphyrin loading into pDefs. Correspondingly, both labeled and unlabeled microbubbles featured 98% fluorescence quenching in the intact state that was restored with surfactant‐induced particle disruption (Figure 2E). Copper‐labeling slightly increased C16 pDef fluorescence quenching efficiency. However, as can be seen in Figure 2F, both labeled and unlabeled microbubbles were fluorescently silent on hyperspectral fluorescence imaging and generated strong fluorescence signal post‐disruption. This state‐dependent fluorescence activation of Cu‐pDef is foundational to qualitatively characterizing the timing and location of bulk pDef cell uptake and disaggregation in vivo. These photonic properties were shared among all explored pDef formulations (Figure S7, Supporting Information). A complete summary of photonic properties for pDefs can be found in Table S2 (Supporting Information). The combined analytical characterization of pDefs following our one‐pot Cu chelation protocol clearly demonstrated efficient, purification‐free Cu‐labeling of diversely composed lipid microbubbles in a manner that conserved their size, yield, stability, and photonic properties. This new achievement in lipid microbubble chemistry enabled confident acquisition of pharmacokinetic data that accurately represented characteristics of unlabeled parent formulations used as‐is for MB‐FUS applications.

### 2.3 Size and Chelation Stability of Cu‐pDef Nanoprogeny Following Flow and FUS Application in Phantom

**Figure 3:**
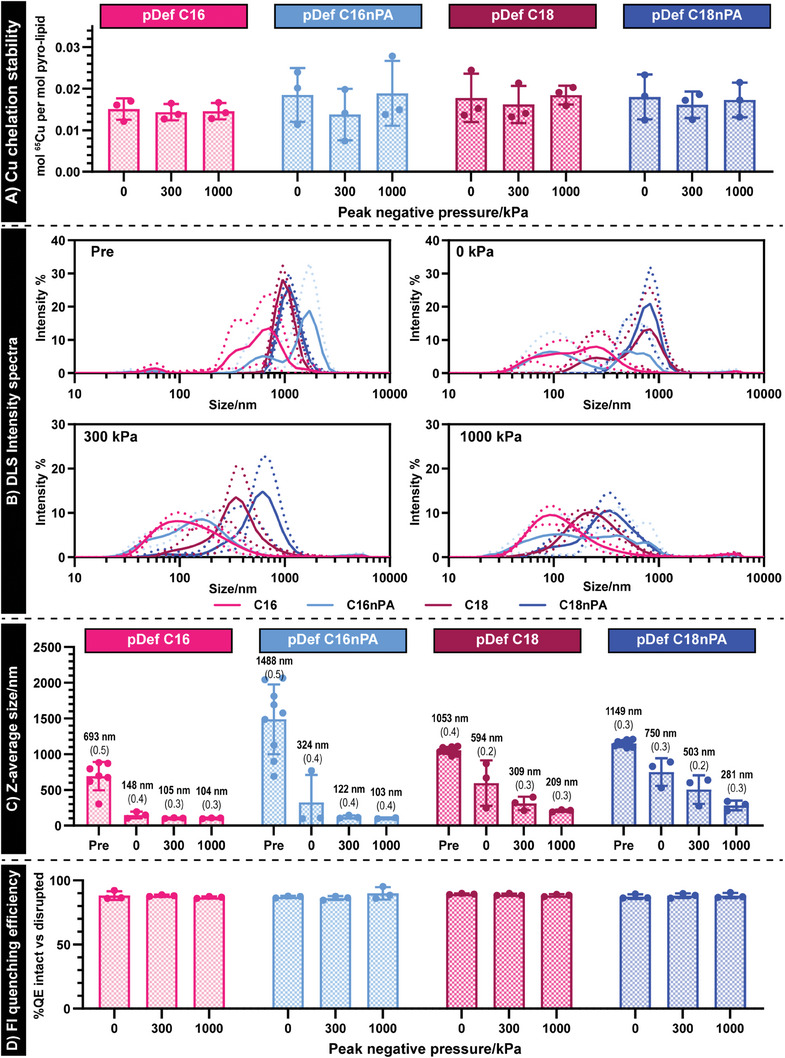
The nanoprogeny generated by microbubbles following FUS exposure are formulation dependent. A) Exposure to low (300 kPa) or high (1000 kPa) peak negative pressure FUS did not affect chelation stability of pDefs, as evident by retained Cu:poprhyin rations. B) Circulation and sonication within a flow phantom results in gas loss and differential structural changes in pDefs, with C) C16 chain length pDefs transitioning to nanostructures 2‐3‐fold smaller than C18 chain length pDefs (mean Z‐average in bold, PDI in brackets). D) Quenching efficiency remained high following ultrasound exposure, confirming pDefs formed daughter nanostructures and not monomeric components (measured via CLARIOstar plate reader).

“Cold” copper‐labeled pDefs were subjected to FUS to characterize the sono‐stability of the Cu‐porphyrin chelation and to ascertain the bulk effects of flow and sonication on pDef shell fate, namely size and supramolecular structure retention. The Cu‐pDefs were passed through an agar flow phantom and exposed to FUS at both high (1000 kPa) and low (300 kPa) peak negative pressures using microbubble concentrations expected in blood following intravenous administration and comparable pulsing strategies as those applied in vivo. Any freed copper was then removed (Figure S8, Supporting Information) and Cu:porphyrin ratios were quantified via inductively coupled plasma mass spectrometry (ICP‐MS). As illustrated in **Figure** **3**A, there was no change in this ratio with sonication at either 300 kPa or 1000 kPa (one‐tailed t‐test, *p* < 0.05). This suggests that FUS irradiation, even at high peak negative pressures, does not disrupt Cu chelation to pDef porphyrin moieties, thereby validating the high sono‐stability of the Cu‐pDef labeling and supporting the use of **[** ^64^Cu**]**Cu‐porphyrin‐lipid as a faithful proxy to monitor microbubble shell fate for both intact and fragmented forms.

Furthermore, sizing of these agents after exposure to flow and FUS (Figure 3B,C) illustrates formulation‐dependent differences in end‐point population sizes. It should be noted that slight differences between Coulter and DLS sizing of nascent microbubbles was expected due to DLS limitations in accurately sizing micron‐sized buoyant species. However, DLS, unlike Coulter Counting, allows for sizing down to 0.5 nm, facilitating more accurate nanoprogeny sizing. Through DLS, it was observed that even in the absence of ultrasound, pDefs decreased in size after flowing through a vascular phantom at 37 °C. C16 chain length pDefs exhibited greater flow‐induced size reductions (4.5‐fold reduction compared to pre‐flow size) than C18 chain length pDefs (1.8 and 1.5‐fold decrease for C18 and C18nPA pDefs, respectively). Sonication at either 300 or 1000 kPa peak negative pressures resulted in further reductions in size, with both pressures yielding nanostructures populations from C16 chain length pDefs of ≈100 nm in size, which were 2–3‐fold smaller than particles generated from C18 chain length pDefs with and without the anionic PA moiety. This suggests that these populations reflect a semi‐stable nanosized state to which these microbubbles may convert as they lose their gas content in circulation. These nanostructures retained high fluorescence quenching efficiencies, and thereby stable supramolecular lipid ordering rather than disordered species (Figure 3D). Collectively, these sizing and fluorescence results confirm in vitro literature reports of individual C16 chain length microbubbles forming smaller, more fragmented daughter structures post FUS compared to C18 chain length microbubbles,^[^ ^73^ ^]^ extending these findings to the bulk in‐solution level for the first time. These differences in the sizes of circulation microbubble daughter nanostructures likely contribute to overall differences seen in pDef lipid shell pharmacokinetic and biodistributions, as the nanomedicine field has strongly linked these physical properties to biological fate.^[^ ^74^, ^75^, ^76^ ^]^

### 2.4 Microbubble Core and Shell Circulation Kinetics are Affected by Shell Composition

We next sought to determine if different pDef compositions also yielded differential microbubble clearance kinetics in vivo. As previously discussed, lipid microbubble circulation kinetics have predominantly examined minutes‐long core perfluorocarbon gas dissolution through ultrasonography and, more minimally, the elimination of perfluorocarbon gas in the blood and lungs through gas chromatography.^[^ ^28^, ^29^ ^]^ These measures have been used as surrogates for overall lipid microbubble blood clearance, with no studies that have clearly quantified microbubble lipid shell circulation clearance beyond a 60 min post‐injection timeframe.^[^ ^30^, ^31^ ^]^ In the realm of therapeutic microbubble applications, such characterization is insufficient and such surrogacy is likely inaccurate, particularly when considering the known phenomenon of microbubble fragmentation into daughter nanoparticles and the known hours‐long circulation half‐lives of lipid nanoparticles.^[^ ^62^ ^]^ Thus, to gain a more complete understanding of lipid microbubble circulation kinetics, we obtained complementary circulation pharmacokinetic measures of both microbubbles in their gas‐containing state and their residual, likely longer‐circulating lipid shell components. This allowed us to determine if gas dissolution studies can in fact be used as surrogates for predicting microbubble lipid shell blood‐clearance trends and also to appropriately select timeframes^[^ ^47^ ^]^ for subsequent PET biodistribution characterization of pDef shell fate.

**Figure 4:**
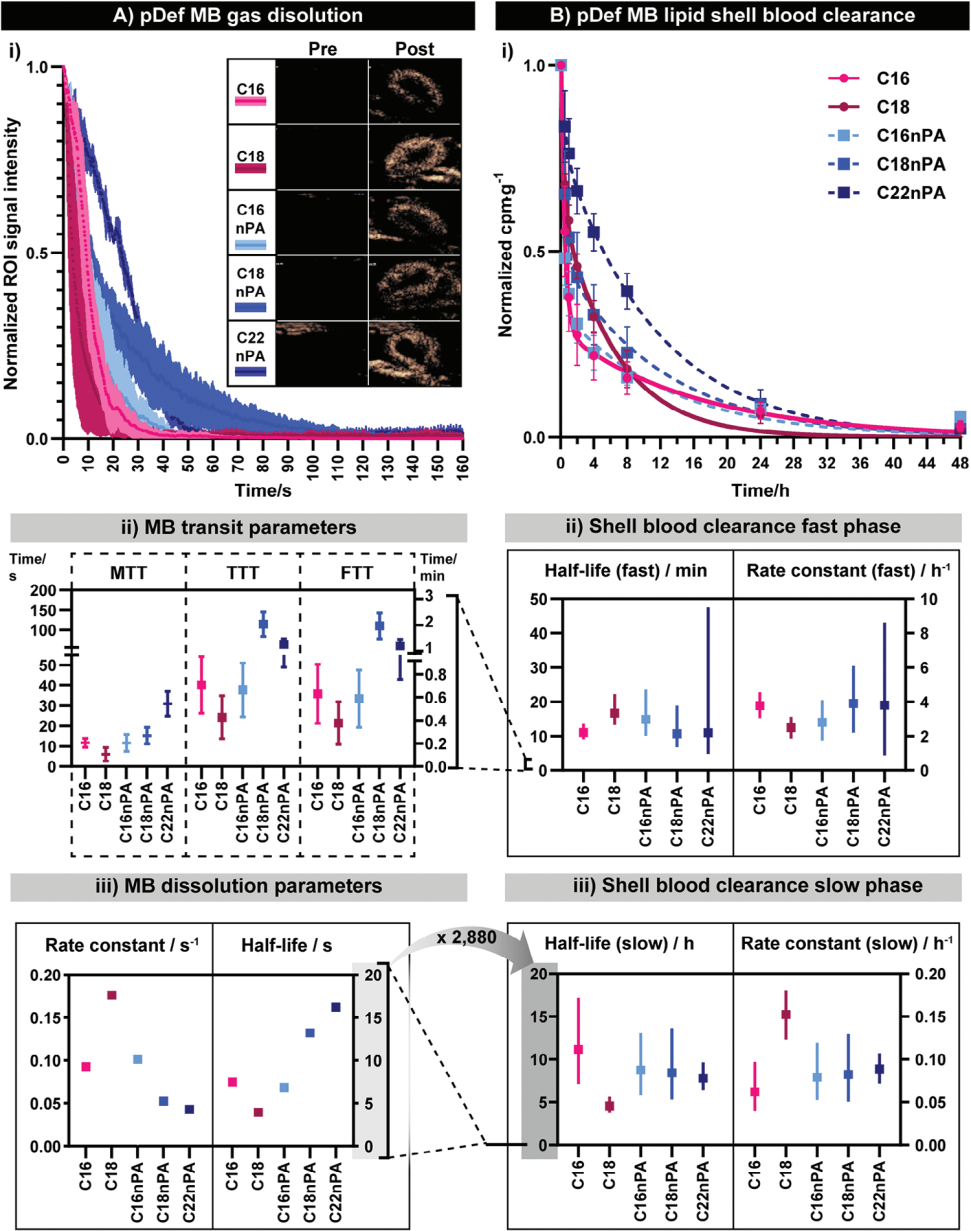
Full pharmacokinetic clearance profiling of pDefs and their shell fragments through complementary ultrasonic and blood tracking. A) (i) Non‐linear contrast imaging of microbubbles captures formulation‐dependent wash out and gas dissolution. (ii) Loss of contrast analysis shows that mean contrast time (MTT), total transit time (TTT), and falling time (FT) vary with chain length (*n* = 3, mean ± standard deviation), while (iii) curve modeling produces pDef rate constants and half‐lives on the order of minutes. In contrast, B) (i) tracking blood clearance of microbubble shell fragments of 48 h shows an extended circulation profile. Clearance rates fit by a two‐phase exponential decay (t‐test vs one‐phase exponential decay, *p* < 0.05) shows (ii) fast and (iii) slow half‐lives with different formulation‐dependencies. This provides a total microbubble blood persistence profile that is almost 3000‐fold longer than that suggested by wash‐out imaging alone. Rate parameters are displayed as mean ± 95% CI. CIs too small to be seen in 4Aiii are listed alongside all other kinetic parameters in Table S3 (Supporting Information).

Microbubble gas dissolution was evaluated through non‐linear contrast imaging of healthy BALB/c mice following the bolus injection of the pDefs (2.5 × 10^7^ microbubbles per mouse) through a tail vein catheter. All pDefs provided vascular contrast immediately following injection, visualized within the highly perfused kidney, which expectedly diminished within 2 min (**Figure** **4**A‐i). Quantification of the contrast evolution in the renal cortex over time revealed differential trends in charged and uncharged pDef transit times (Figure 4A‐ii) and dissolution rates (Figure 4A‐iii). The neutral pDefs (C16nPA, C18nPA) showed an increase in mean, total, and falling transit time, as well as an increased dissolution half‐life with increasing lipid chain length (t_1/2_ 6.84 s, 95% CI [6.73–6.96 s] and t_1/2_ 13.2 s, 95% CI [13.1–13.3 s for C16nPA and C18nPA, respectively), a trend that corroborates with the established literature.^[^ ^65^, ^66^ ^]^ Neutral pDefs composed of C22 chain length lipids (C22nPA) were added to further illustrate this trend, which showed a further lengthening of mean transit time and dissolution half‐life (t_1/2_ 16.2 s, 95% CI [16.1–16.4 s]). Nevertheless, the C18nPA MBs, due to their extended falling time, were visualizable longer than all other MBs. The opposite chain length trend was noted for neutral pDefs: the C16 pDef variant exhibited a statistically insignificant increase in all transit parameters but significantly slower dissolution (t_1/2_ 7.47 s, 95%CI [7.38, 7.57]) than its longer chain length C18 pDef variant (t_1/2_ 3.93 s, 95% CI [3.85, 4.01]). When comparing microbubbles of the same chain length, the presence of charge hastened dissolution of C18 chain length pDefs and had no effect for C16 chain length pDefs.

These compositional effects were consistent with other forms of linear regression modeling,^[^ ^77^, ^78^ ^]^ including selective modeling of the “wash‐out” phase of the curve and total curve modelling using a gamma variate function (Figure S9 and Table S3, Supporting Information). Overall, all pDefs displayed faster microbubble dissolution than an in‐house formulation of commercial Definity (Table S3, Supporting Information) but comparable in vivo contrast kinetics and trends to other non‐commercial, neutral lipid MBs.^[^ ^65^, ^66^ ^]^ While prior studies attribute the slower microbubble gas dissolution of longer acyl chain length bubbles to respective increases in microbubble shell stiffness,^[^ ^66^ ^]^ this deviation from the trend observed for anionic pDefs may suggest that factors beyond shell stiffness could destabilize microbubbles within the biological milieu and hasten shell clearance.

We illuminated this possibility by quantifying the blood clearance of ^64^Cu‐labeled pDef lipid shells, thus capturing the full circulation profile of this multi‐component platform (Figure 4B). The radiolabeled microbubbles were administered intravenously, after which blood was serially collected from the saphenous vein and analyzed by γ‐counting to measure circulating pDef shell presence over 48 h. All pDef shells, regardless of composition, were eliminated from circulation over the course of 24–48 h post‐injection (Figure 4Bi). Their clearance profiles were best fit by two‐phase exponential functions. This gave each formulation a “fast” and “slow” half‐life of shell fragment elimination from circulation akin respectively to “distribution” and “elimination” phases of a two‐compartment model. Fast and slow half‐lives ranged from 14–19 min and 4.5–11 h, respectively (Figure 4B‐ii,iii; Table S4, Supporting Information). All pDef shells exhibited similar rates of slow clearance except for pDef C18, which had a 4.5 h clearance half‐life that was 2–3‐fold lower than its C16 pDef variant (11.2 h) and neutral pDefs (t_1/2_ of 8.75 h, 8.43 h, and 7.8 h for pDefs C16nPA, C18nPA, and C22nPA, respectively). While the slow clearance phase of neutral pDefs did not exhibit such a reduction in circulation half‐life with chain lengthening, the fraction of shells removed during the fast (distribution) phase of circulation decreased with increasing chain length (Figure 4Bi), yielding overall higher area under the curve (AUCs, Table S5, Supporting Information) for longer chain lengths (5.6 ± 0.9, 7 ± 2, and 10 ± 2 normalized mCi·h for C16, C18, and C22nPA pDefs, respectively). No significant difference was observed between C16 and C16nPA pDefs with respect to clearance rates, % fast phase, or circulation AUCs, highlighting the variable impact of anionic PA lipid on both microbubble and shell circulation kinetics.

Collectively these blood clearance data demonstrate: 1) there is no consistent effect of anionic PA lipid on microbubble shell circulation kinetics, 2) chain lengthening hastens clearance of anionic lipid microbubble shells significantly and appreciably, while only minimally and statistically insignificantly hastening neutral lipid microbubble shell clearance, and 3) chain lengthening increases the overall blood pool exposure of neutral lipid shells. The implications of these trends for strategic MB‐FUS platform design will be discussed in Section 2.8. Finally, it was confirmed that porphyrin lipid inclusion into these microbubble systems did not significantly change circulation half‐life parameters, as evaluated by reducing inclusion fractions from 30 mol% to 10 and to 1 mol.% (Figure S9 and Table S4, Supporting Information) with little change in shell pharmacokinetics. This broadens the significance of the above shell clearance trends to the wider microbubble scientific community. Overall, this complementary approach to quantification of microbubble core dissolution and shell fragment circulation kinetics allowed us to confirm that a timeframe up to 48 h was necessary for subsequent PET biodistribution studies to fully capture pDef shell fate. Importantly, these shell clearance timeframes are three orders of magnitude higher than microbubble core dissolution rates and more consistently align with clearance profiles of lipid nanoparticles. This highlights the importance of acquiring pharmacokinetic data using measures relevant to a microbubble's intended application.

To investigate whether these formulation‐dependent changes in circulation might be attributable to their interactions with serum proteins, degree of interaction and destabilization was investigated in vitro (Figure S10, Supporting Information). Following brief incubation of microbubbles with serum, a BCA assay showed no difference in fast, non‐specific protein binding across all pDefs (Figure S10A, Supporting Information). However, in situ serum incubation over 12 h did reveal that chain length shortening, and to a lesser extent anionic charge, resulted in greater degrees of disruption of ordered porphyrin aggregation in pDefs, indicating more lipid‐protein interactions for these formulations over extended timescales (Figure S10B, Supporting Information). While these findings are not sufficient to fully explain the observed trends in microbubble and fragment circulation, they encourage more in‐depth investigation of discrete opsonin binding and specific elimination pathways in an in vivo context.

### 2.5 Establishing Microbubble Shell Elimination Pathways and PET/CT Quantification Protocol in Healthy Animals

**Figure 5:**
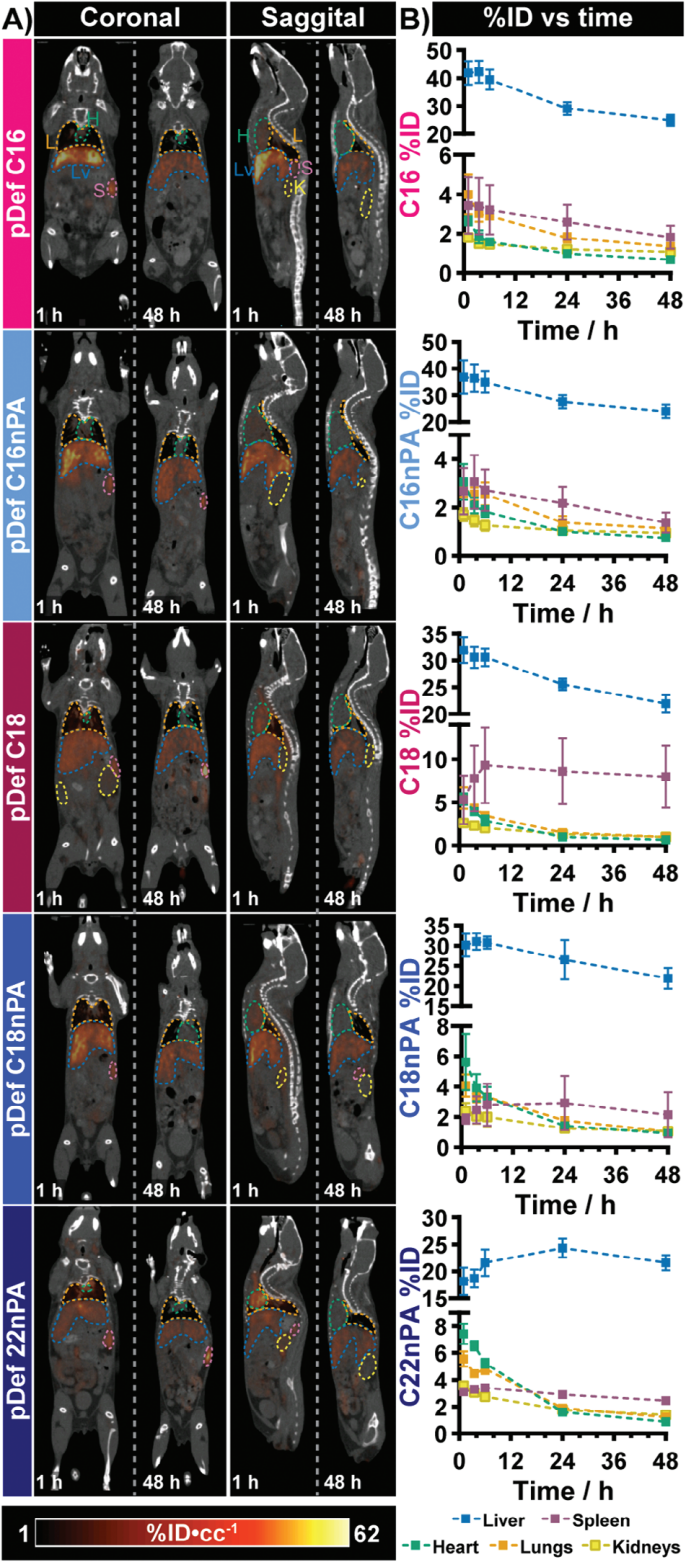
Establishing pDef shell biodistribution imaging workflow in healthy animals. A) Sample PET/CT coronal and sagittal views at early (1 h) and late (48 h) timepoints across 5 different pDef variants. Colored outlines illustrate slices of 3D contours manually constructed for all organs at all timepoints, where H: heart (green), L: lungs (orange), K: kidneys (yellow), LV: liver (blue), and S: spleen (purple). Dynamic range of PET is 1–62% ID cc^−1^. B) Longitudinal quantitative biodistribution from PET imaging of fractional injected dose (%ID), comparing relative organ uptake over time for each pDef formulation.

Building upon the knowledge that pDef formulation impacts microbubble and shell fragment blood clearance, we next applied the ^64^Cu‐radiolabeled pDefs in healthy BALB/c mice to validate a PET/CT protocol for microbubble shell kinetic biodistribution quantification and elucidate clearance pathways. The **[** ^64^Cu**]**Cu‐pDefs were administered intravenously (0.5–0.6 mCi, 20 nmol porphyrin, and ∼2 × 10^8^ microbubbles per animal) to mice, which were serially imaged using PET and CT at 1, 3.5, 6, 24, and 48 h post‐injection to encompass the circulation lifetime of pDef shells.^[^ ^47^ ^]^ Volumetric 3D contours were manually constructed for each data volume to quantify PET signal within major organs of interest (heart, lungs, kidneys, liver, spleen).^[^ ^79^ ^]^ Quantification of this signal per organ volume at each timepoint captured the full course of pDef transit through the body (**Figure** **5**; Figures S11–S14, Supporting Information). At 48 h post‐injection, mice were euthanized and dissected to harvest 14 major organs and tissues, which were subjected to γ‐counting to quantify percentage accumulation of the injected pDef radioactive dose (%ID). This was compared to associated PET/CT quantification to validate the latter's accuracy (Figure S13, Supporting Information).

In brief, the brightest PET signal was observed in the liver and spleen for all variants (Figure 5A; Figures S11 and S12, Supporting Information), with the liver exhibiting the highest %ID of all pDef shells (Figure 5B). Over the 48 h of study, liver PET signals peaked at 6 or 24 h post‐injection and then monotonically decayed thereafter. When normalized by organ volume, the spleen was also found to be a major site of microbubble shell accumulation (Figures S13A,B, Supporting Information), with an accumulation profile that was either stable across the duration of imaging or progressively increased depending on the pDef formulation. Of particular note, the rapid blood clearance experienced by the pDef C18 variant may be partially attributable to its appreciably higher and faster splenic uptake compared to other variants. These retention‐dominated signal profiles in the liver and spleen contrasted the perfusion‐dominated signal in the heart, lungs, and kidneys. PET signal within these three organs was highest at the earliest imaging timepoint and subsequently decayed over time, yielding pharmacokinetic profiles similar to pDef blood clearance (Figure S13C, Supporting Information). In fact, modeling of the heart PET time series yielded statistically indifferent clearance half‐lives to those obtained in pDef circulation studies. This suggests that heart PET signal can appropriately be used as a proxy to model microbubble shell circulation kinetics, a technique previously used for such modeling 1 h post‐injection,^[^ ^30^, ^31^ ^]^ and which is now more explicitly supported by this work across diverse microbubble formulations and over the entirety of microbubble shell circulation lifetimes. This was enabled by the effective and stable chelation of **[** ^64^Cu**]**Cu^2+^ to microbubble lipid shells, exemplified by the lack of any observable free radioisotope PET signal in the bladder at any timepoint. PET signal quantification also corroborated well with end‐point organ γ‐counting (Figure S13D, S14 Supporting Information) of pDef shell %ID accumulation in all five contoured organs.

Overall, these data support a previously assumed and now appropriately evidenced claim that lipid‐shelled MBs undergo a hepatobiliary/fecal mode of clearance. These data notably differ from prior work in which lungs were reported as a primary site of microbubble shell accumulation (even achieving higher %ID cc^−1^ or g^−1^ than the liver).^[^ ^30^, ^32^ ^]^ Due to a lack of microbubble sizing in those papers, it is unclear if such lung accumulation was due to the administration of larger sized microbubbles that were inadvertently trapped in pulmonary capillaries, artificially shortening their blood clearance half‐lives and increasing pulmonary deposition. Previous focus on the lungs may have been a remnant of early microbubble work analyzing the dynamics and safety of gas clearance^[^ ^28^, ^29^ ^]^ for which the lungs were an obvious organ of importance due to their role in gas exchange. However, current evidence does not suggest that microbubble shells themselves remain entrapped preferentially in the lungs from a perspective of gross uptake. Our data also challenges renal excretion as a primary mode of microbubble lipid shell clearance, suggesting previous observations of bladder uptake of radiolabeled microbubbles may have been a result of unstable chelation. This further highlights the importance of microbubble characterization prior to in vivo applications.

### 2.6 Influence of Microbubble Composition and FUS Application on Organ Biodistribution and Accumulation

**Figure 6:**
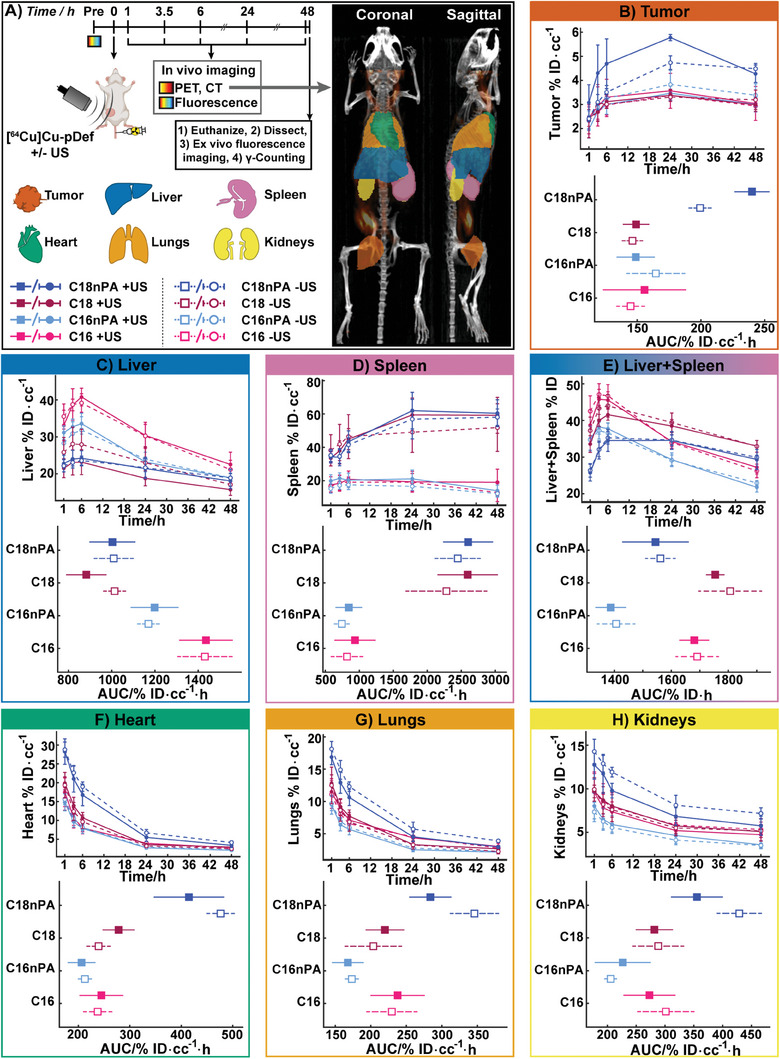
Impact of FUS and shell chemistry on pDef biodistribution in tumor‐bearing animals. A) Experimental timeline for combined PET, CT, and hyperspectral fluorescence imaging of the pDef platform. Legend illustrates filled/unfilled markers correspond to FUS‐treated and FUS‐untreated animals, respectively. Circular markers correspond to %ID cc^−1^ are displayed as mean ± SD, while square markers correspond to AUCs and are displayed as mean ± 95% CIs. Maximum intensity projection views of sample mouse illustrate orthotopic placement of 4T1 breast carcinoma at the inguinal mammary fat pad. Quantitative PET‐based kinetic biodistribution time series over 48 h (%ID cc^−1^) and integrated area‐of‐curve (AUC) total dose exposure plots (%ID·cc^−1^ h) compares formulation‐specific uptake for B) tumor, C) liver, D) spleen, E) hepatosplenic (liver+spleen, presented as %ID only), F) heart, G) lungs, and H) kidneys.

Guided by the workflow and PET/CT quantification feasibilities established in healthy animals, further experiments were conducted in tumor‐bearing mice as a more relevant model for therapeutic applications of MB‐FUS. Female BALB/c mice bearing 4T1 orthotopic breast tumors were administered [^64^Cu]Cu‐pDefs and imaged via PET/CT serially as described for healthy mice. Mice also underwent whole‐body in vivo hyperspectral fluorescence imaging prior to pDef injection and after PET/CT scans at each timepoint. A subset of the animals received FUS treatment at the tumor site immediately following [^64^Cu]Cu‐pDef injection. Successful microbubble and FUS delivery at the tumor site were confirmed by passive acoustic detection (Figure S15, Supporting Information). Following euthanasia and dissection, organs were subjected to fluorescence imaging before undergoing γ‐counting. Manual contouring of PET/CT images was conducted on the liver, spleen, heart, lungs, kidneys, and tumor. Examples of these contours and a summary of the experimental timeline are provided in **Figure** **6**A. Data is presented primarily as %ID cc^−1^ for kinetic biodistribution plots or %ID cc^−1^ h for time‐integrated AUCs of total dose exposure (Figure 6), allowing for an accurate comparison of composition and FUS effects on pDef shell pharmacokinetics without confounding differences in organ and tumor volumes between experimental groups. Representative PET/CT and fluorescence images for C16, C16nPA, C18, and C18nPA pDef treatment groups are respectively provided in Figures S16–S19 (Supporting Information). Cumulative dose exposure for each organ in the form of %ID AUCs are provided in Figure S20 (Supporting Information). A breakdown of the organ, compositional, and ultrasound‐enabled differences as interpreted through both longitudinal and end‐point PET and fluorescence analysis is provided below, with detailed statistics and quantification of relative changes associated with chain length, charge, and FUS provided in Figures S21–S23 (Supporting Information), respectively.

#### 2.6.1 Microbubble Chain Length Impacts Liver and Spleen Accumulation and Particle Disruption

Similarly to healthy animals, longitudinal biodistribution showed that all pDefs were found to primarily transit and reside throughout the liver and spleen over a 48 h period, achieving the brightest PET signal (Figures S16–S19, Supporting Information) and greatest degree of total uptake across all timepoints compared to the other organs (Figure S20, Supporting Information). Within the liver, MB composition had a clear influence on liver signal over time. C16 and C16nPA pDefs displayed significantly higher shell fragment liver accumulation compared to the longer chain length C18 and C18nPA analogues, suggesting that microbubbles of a shorter chain length are preferentially targeted to the liver (Figure 6C; Figure S21A, Supporting Information). This enhancement with shorter chain length was most pronounced for the anionic C16 pDef + FUS group, demonstrating a 1.6‐fold enhancement in AUC compared to its C18 variant. When integrated across the entire time series, C16 pDefs exhibited the highest liver retention, with a slightly higher AUC than their neutral C16nPA pDef counterparts (Figure 6C; Figures S22A, Supporting Information). However, there was no difference seen between C18PA and C18nPA AUCs, indicating only a partial impact of MB charge on liver accumulation. These differences between MB formulations held regardless of the presence or absence of applied FUS at the tumor site. When comparing liver signal in ultrasound‐treated and untreated mice, it is seen that ultrasound treatment has no significant impact on liver uptake (%ID·cc^‐1^ or AUC) for any microbubble formulation (Figure S23, Supporting Information).

All microbubbles display similar residual liver radioactivity by 48 h in both the PET and γ‐counting (Figure S24, Supporting Information) measurements, which predict statistically equivalent values to one another at endpoint for all formulations (Figure S25, Supporting Information). PET quantification was found to align 1:1 with γ‐counting (Figure S25, Supporting Information) across all microbubble formulations and organs. However, fluorescence activation in the liver as assessed with ex vivo hyperspectral fluorescence misrepresents the magnitude of this trend: while γ‐counting correctly demonstrates a maximum 1.5‐fold increase in liver accumulation with chain shortening from C18 to C16, fluorescence displays a 4–6‐fold higher intensity for C16 and C16nPA pDefs compared to C18 and C18nPA pDefs (Figures S26 and S27, Supporting Information) in the presence and absence of FUS. This highlights an important distinction: radiometric readouts depend solely on absolute shell accumulation, while fluorometric readouts are affected by both shell accumulation and degree of disaggregation of the supramolecular structure. Thus, fluoroscence imaging is unreliable as a means of quantifying microbubble shell deposition. Different microbubble formulations may disaggregate at different rates in vivo, as observable in Figure S28 (Supporting Information), wherein C16 chain length pDefs exhibit higher fluorescence signal per corrected radioactivity (i.e., higher fluorescence unquenching) in the liver than C18 chain length pDefs. This suggests that C16 and C16nPA pDefs underwent higher levels of particle disruption into monomeric lipids than C18 and C18nPA pDefs. This observation aligned with in vitro serum stability tests that showed faster disordering of porphyrins in the microbubble shell for C16 chain lengths than C18 chain lengths. Charge and FUS did not affect microbubble disruption in the liver.

The spleen displayed the most dramatic differences in quantitative uptake between microbubble formulations. C18 chain length pDefs displayed notably higher accumulation than C16 chain length pDefs at all imaged timepoints, as well as in integrated AUCs (Figure 6; Figure S21, Supporting Information). This C18 splenic uptake rivaled that of the liver, despite the ≈10‐fold difference in size of the two organs. The impact of microbubble lipid chain lengthening was such that the cumulative %ID in the spleen rose by over 500% into magnitudes associated with liver accumulation, simply by lengthening the microbubble chain length by two carbon units (Figure S20, Supporting Information). This increase in splenic accumulation with microbubble chain lengthening strengthened over the imaging timeframe, initiating with a 1.8–2.4‐fold enhancement at 1 h post‐injection and ending with a 3.3–4.8‐fold enhancement at 48 h post‐injection for C18 chain length microbubbles compared to C16 chain length microbubbles (Figure S21B, Supporting Information). This was due to differential uptake kinetics between the microbubble types. Whereas C16 chain length pDefs displayed a persistent or decreasing radioactive signal across 48 h (Figure 6; Figures S16 and S17, Supporting Information), the C18 chain length pDefs’ PET signal gradually increased over 24 to 48 h post‐injection (Figure 6; Figures S18 and S19, Supporting Information). This persistence in splenic accumulation between 24–48 h was not seen in any of the other analyzed organs, demonstrating relatively slower microbubble shell elimination from the spleen than from the liver, heart, lungs, kidneys, or tumor. Overall, chain length affected pDef splenic accumulation more strongly than charge or FUS. Incorporation of the anionic PA group into C18 chain length microbubbles slightly decreased splenic uptake (0.6‐fold change) at 48 h, but this effect was made insignificant with FUS application (Figure S22B, Supporting Information). Beyond this effect on C18 pDef splenic uptake, FUS had negligible impact on pDef uptake (Figure S23B Supporting Information).

These splenic findings are supported by ex vivo γ‐counting of dissected spleens, although their relative magnitudes differ, with γ‐counting predicting an even higher 6–10‐fold difference between longer and shorter chain‐length microbubbles (Figures S21, S24, and S25, Supporting Information). They are also supported by nanomedicine literature,^[^ ^75^, ^76^ ^]^ which showcases higher splenic accumulation of liposomes with larger particle size (like the larger nanostructures created from C18 chain length pDefs). Ex vivo fluorescence imaging showed ≈3‐fold (no FUS) and 2‐fold (with FUS) higher fluorescence intensities in the spleen from C18 chain length groups compared to C16 chain length groups, with no statistically significant change elicited with PA inclusion or FUS treatment (Figures S26 and S27, Supporting Information). Nevertheless, despite exhibiting absolute lower fluorescence intensities, C16 chain length microbubbles still underwent greater degrees of fluorescence unquenching in the spleen than C18 chain length microbubbles (Figure S28B, Supporting Information), with the presence of PA amplifying this chain length‐dependent unquenching. Thus, C16 lipid chain length microbubbles exhibit lower splenic accumulation but higher particle disruption in the spleen than C18 chain length microbubbles.

This splenic tropism may be attributable to the larger size of C18 chain length pDefs (∼200–300 nm) relative to C16 chain length pDefs (∼100 nm). While both C16 and C18 nanoprogeny can enter murine splenic sinusoids based on their pore size,^[^ ^80^, ^81^ ^]^ larger and less deformable particles are more likely to be retained within these structures or their resident mononuclear phagocytic system elements. Such size‐induced splenic tropism could be further strengthened by the more rapid disaggregation of C16 chain length pDef shells. Our in vitro serum studies as well as in vivo fluorescence imaging demonstrated C16 shells to undergo faster disordering and fluorescence unquenching. This would result in the more rapid formation of C16 monomeric lipid entities than C18 chain length shells, further widening the size gap between circulating C16 and C18 nano‐fragments. We encourage researchers to investigate this mechanism further using histological and cell‐specific techniques.

By combining total signal within the spleen and liver, we can approach a fuller representation of the primary microbubble processing centres for different formulations (Figure 6E, %ID of liver+spleen). This illustrates that, for all formulations, uptake across the liver and spleen occurs predominantly over the first 6 h following injection. This is complemented by the fast fluorescence activation observed in vivo over areas corresponding to the liver and spleen, as early as 1 h post‐injection (Figures S16–S19, Supporting Information). This rapid and sustained fluorescence activation suggests ready splenic and liver uptake and disruption of microbubble fragments. After 6 h, PET signal from C16 chain length microbubbles cleared more quickly than C18 microbubbles due to a greater fraction being cleared by the liver and its associated kinetic distribution profile, compared to the spleen. When quantified over the length of the time series, AUCs shows that the presence of PA appears to increase total residency across the liver‐spleen axis over 48 h for both the C16 and C18 chain length microbubbles, a trend which was not statistically resolvable when examining purely liver‐based uptake (Figure 6E, *p* < 0.05). This aligns with nanomedicine literature, which shows higher opsonization and liver/splenic uptake of charged liposomes compared to neutral liposomes.^[^ ^82^, ^83^, ^84^ ^]^ Together, these data firmly support the hypothesis that lipid‐shelled microbubbles are processed primarily by the liver and spleen, and that shell composition affects both the degree and dynamics of uptake within the liver and spleen.

#### 2.6.2 FUS, Chain Length, and Charge do not Consistently Impact Microbubble Shell Presence in the Heart, Lungs, or Kidneys

The heart, lungs, and kidneys of tumor‐bearing mice exhibited monotonic decreases in accumulation over time for all microbubble formulations akin to that observed in healthy mice (Figure 6F–H). C18nPA pDef displayed a higher initial signal in each of these organs compared to C16, C16nPA, and C18 pDefs, consistent with healthy animal data (Figure S13, Supporting Information), but all formulations fell to similar levels of quantified radioactivity within the heart and lungs by 48 h. Nevertheless, C18nPA pDef displayed higher overall uptake (largest AUCs) in the heart, lungs, and kidneys in the absence of FUS treatment. In considering the off‐target effects of tumor‐localized ultrasound treatment on these peripheral organs, FUS treatment decreased the lung and kidney accumulation of C18nPA pDef, but had no effect on the heart, lung, or kidney accumulation of any other microbubble (Figure S23, Supporting Information). Furthermore, FUS treatment did not change signal or accumulation profiles within the heart for any of the microbubble formulations (Figure 6F; Figures S16–S19, Supporting Information), and thus FUS also did not impact associated modeled blood clearance half‐lives of any microbubble shell (Figure S29, Supporting Information). Charge and chain length did not have any consistent impact on pDef accumulation in the heart, lungs, or kidneys (Figures S21 and S22, Supporting Information).

Ex vivo γ‐counting measures of shell accumulation in the heart, lungs, and kidneys were similar to those obtained by PET/CT at the final 48 h timepoint (Figures S24 and S25, Supporting Information). Ex vivo fluorescence signal in the heart and lungs was greater for C16 chain length microbubbles than C18 chain length microbubbles (Figures S26 and S27, Supporting Information), while only C16 pDef demonstrated any substantial fluorescence signal in the kidneys. FUS treatment did not affect fluorescence activation/particle unquenching in any of these organs. Collectively, these data indicated higher pDef fragment disruption in the lungs than in the kidneys or heart, whereby C16 chain length microbubbles were more strongly unquenched than C18 chain length microbubbles (Figure S28, Supporting Information).

#### 2.6.3 Microbubble Composition Influences Ultrasound‐Enabled Delivery to Solid Tumors

Given that tumor delivery is a primary goal of many MB‐FUS applications, a pivotal aim in this study was to assess the impact of microbubble composition and FUS treatment on gross tumor accumulation of lipid microbubble shells (Figure 6B; Figures S30 and S31, Supporting Information). All four explored microbubble formulations displayed an initial increase in shell uptake at the tumor site up to 24 h post‐injection, after which signal either plateaued or slightly decreased (Figure 6B). Three of the four formulations (C16PA, C16nPA, and C18PA) did not differ in overall accumulation relative to one another. In contrast, C18nPA pDef shells displayed higher tumor accumulation than other formulations both at individual imaging timepoints and overall tumor exposure, yielding an AUC 30–40% larger than the other formulations (Figure 6B). This resulted in a 40–60% higher end‐point tumor delivery of C18nPA pDef shells compared to other pDefs as assessed by ex vivo γ‐counting (Figures S24 and S30, Supporting Information). Ex vivo tumor γ‐counting also demonstrated C16nPA pDef shells to accumulate in tumors to a slightly (10%) higher degree than anionic C16 pDef shells.

The delivery response of C18nPA pDefs to FUS also differed from the other microbubbles. Interestingly, the C18nPA pDef shell was the only one that underwent greater deposition in FUS‐treated tumors, whereas C16, C16nPA, and C18 pDef tumor accumulation did not statistically change with FUS treatment (Figure S30, Supporting Information). The C18nPA pDef cumulative shell delivery was enhanced by 20% with FUS treatment, with a maximum FUS‐enabled delivery enhancement at 3.5 h post‐injection of 50%. This aligns with the 3–6 h timeframe generally associated with transient vasculature disruption by MB‐FUS.^[^ ^4^, ^85^ ^]^ Although exhibiting differences in overall drug exposure at the tumor site, no sustained difference in C18nPA pDef tumor accumulation with or without FUS treatment was observed 48 h post‐injection, in agreement with ex vivo γ‐counting.

Similar to fluorescence quantitation in other organs, C16 chain length pDefs yielded higher fluorescence signal (2–3‐fold higher) in the tumor 48 h post injection than C18 chain length pDefs. This suggests enhanced structural unquenching and activation of these smaller porphyrin‐containing structures in tumors (Figure S31A,B, Supporting Information). In fact, the increase in fluorescence relative to γ‐counting was strongest in the tumor (Figure S25, Supporting Information) compared to all other organs. Compared to either the ipsilateral or contralateral healthy mammary fat pads, both fluorescence and γ‐counting measures were over an order of magnitude higher at the tumor site, demonstrating strong targeting of the pDef shell delivery to the tumor versus healthy tissue. It is important to note that this targeted drug delivery was accomplishable in the 4T1 tumors without FUS treatment. Despite exhibiting lower absolute fluorescence signal in tumors ex vivo, C18 chain length pDefs exhibited higher fluorescence contrast at tumor sites in vivo compared to surrounding ipsilateral muscle (Figure S31E, Supporting Information), while C18nPA pDef exhibited the highest tumor fluorescence contrast relative to the contralateral healthy fat pad (Figure S31F, Supporting Information).

These data provided unexpected and compelling insights. First, despite exhibiting different blood clearance half‐lives, microbubble dissolution rates, hepatosplenic uptakes, and daughter nano‐fragment sizes, C18 pDef exhibited similar tumor accumulation as C16 and C16nPA pDefs with or without FUS application. This was unexpected based on inferences translated from the nanomedicine field. The lack of FUS enhancement in tumor delivery of C16, C16nPA, and C18 pDef shells was also surprising. Collectively, this reinforces the importance of tailoring microbubble composition to their intended application to maximize drug delivery, and it highlights the necessity of collecting pharmacokinetic data across microbubble formulations versus relying on unverified trends, suppositions, and generalizations.

### 2.7 Microbubble Fragments Transit Beyond the Vasculature in Tumors and Several Organ Systems

Given that PET signal quantification encapsulates intravascular and extravascular tumor components, it is possible that any FUS enhancement in pDef C16, C16nPA, and C18 shell delivery to extravascular tumor parenchyma was masked by shell fragments still circulating in the intravascular compartment. Perfusion studies were conducted to substantiate this possibility and to better resolve microbubble shell accumulation in the extravascular compartment (inclusive of endothelial uptake). Animals were treated with [^64^Cu]Cu‐pMBs of varying lipid compositions ± FUS and euthanized at 3.5 h post‐injection either through cervical dislocation (unperfused, quantification of intra+extravascular accumulation) or saline perfusion (perfused; removal of circulating shell components isolates quantification of extravascular delivery). This timepoint was selected as it corresponded to the maximum enhancement of C18nPA tumor shell delivery by FUS, already significant accumulation across all studied organs, and for which blood clearance measurements illustrated substantial retention of injected microbubble shells within circulation.

**Figure 7:**
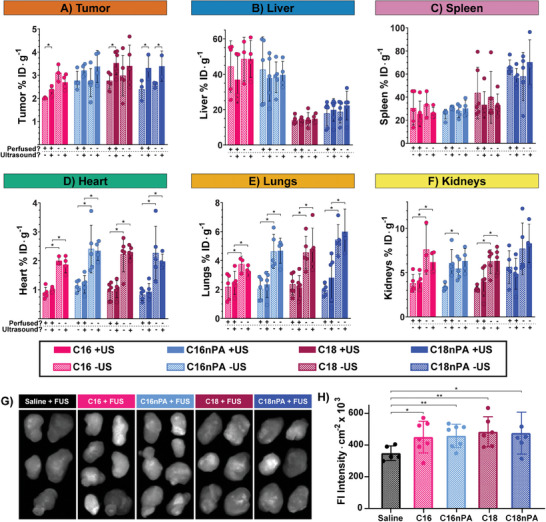
Revealing extravascular deposition of microbubble shell fragments / drug delivery using perfusion. Sacrifice at 3.5 h post‐injection followed by either whole body perfusion or simple dissection and end‐point γ‐counting illustrates formulation‐dependent differences in shell fragment uptake in the A) tumor, B) liver, C) spleen, D), heart, E) lungs, and F) kidneys. G) Persistent vasculature leakage into tumors following MB‐FUS treatment was assessed using Evans blue dye and hyperspectral fluorescence imaging, F) which showed increased uptake for all MB‐FUS treatment platforms. All values are displayed as mean ± SD. Statistics performed were one‐tailed t‐tests (*p < 0.05, adjusted for multiple comparisons using Benjamini‐Hochberg correction).

Removal of intravascular, circulating pDef shells did indeed unmask FUS amplification of shell delivery to tumor parenchyma (**Figure** **7**; Figures S32B and S33A, Supporting Information). Perfused mice receiving FUS treatment showed statistically enhanced tumor accumulation of C16 and C18 pDefs (1.1 fold and 1.4 fold enhancements, p = 0.019 and p = 0.041, respectively, one‐tailed t‐tests) and statistically insignificant enhancement of C16nPA pDef shell delivery (1.2‐fold enhancement, p = 0.061, one‐tailed t‐test). C18nPA pDef shell fragment delivery to tumors continued to be enhanced by FUS treatment in perfused animals (1.3‐fold enhancement, p = 0.0074, one‐tailed t‐test). Inclusion of the anionic PA group slightly but statistically decreased shell tumor uptake with FUS for C16 chain length microbubbles (30% decrease, p = 0.0015), but not for C18 chain length microbubbles (10% decrease, p = 0.602). There was no statistically significant impact of chain length on extravascular microbubble shell delivery at this early timepoint. Chain lengthening had a reduced impact on fluorescence activation in these tumors (Figures S34 and S35, Supporting Information) than at 48 h post‐injection, suggesting that a longer timeframe is required to observe differential particle disaggregation. Furthermore, 20–50% of this fluorescence signal was from intravascular porphyrin unquenching, suggesting that pDef fragment disruption occurred both in the blood stream (possibly from protein binding) and in tissue parenchyma (possibly from cell uptake). Meanwhile, comparing perfused to unperfused animals in either the ultrasound‐treated or untreated groups showed no difference in overall accumulation for all formulations except C16 pDef (Figure S33D, Supporting Information). Collectively, this confirms that microbubbles exhibit high levels of passive extravascular uptake in 4T1 tumors, which is enhanced by sonication.

Evans blue dye was used to determine if these compositional differences extended to differential vasculature permeabilization and delivery of co‐administered agents in tumors. Evans blue remains predominantly bound to circulating serum albumin, and thus its uptake into tissue can be viewed as a sensitive proxy of how small but typically vascularly‐confined loads may exit blood vessels following FUS disruption. All microbubble formulations in conjunction with applied FUS increased Evans blue uptake at the tumor site of perfused animals relative to ultrasound‐treated animals injected with a saline control (Figure 7G,H). There was no significant difference seen in the magnitude of increase between the different formulations. This suggests that that the observed compositional effects on microbubble shell delivery did not correspond to differences in bulk vasculature effects that would change the delivery of co‐administered agents.

Overall, this study demonstrated substantially lower enhancement of microbubble shell delivery to tumors with FUS (maximum 1.5‐fold enhancement) than previous reports of shell‐loaded microbubble delivery (ranging from 2–15‐fold enhancement with FUS in extracranial tumors^[^ ^86^, ^87^, ^88^, ^89^, ^90^, ^91^, ^92^, ^93^ ^]^). However, it is important to recognize that many of such studies make use of ex vivo fluorescence imaging to assess differential drug delivery (Figure S1, Supporting Information and references^[^ ^86^, ^87^, ^88^, ^92^, ^94^ ^]^). Fluorescence quenching, scattering, and depth limitations make fluorescence imaging largely qualitative and inaccurate in its ability to describe pharmacokinetics.^[^ ^60^, ^61^ ^]^ Within this study, we found several instances wherein fluorescence and nuclear imaging data not only varied in the predicted quantities of agent in different body regions (Figure S25, Supporting Information), but also relative tissue uptake trends between different formulations (e.g., overpredicting tumor uptake by 4–200‐fold, overpredicting FUS effects by increasing particle disruption, and exhibiting differential fluorescence unquenching of C16 and C18 chain length particles). Thus, use of fluorescence as the primary pharmacokinetic readout in our study would have yielded substantially different – and incorrect – conclusions about the impact of formulation on MB‐FUS delivery. Given that the tumor is often the target tissue of delivery studies, errors of this magnitude are a significant confounding factor that can exaggerate the degree of shell‐loaded agent delivery and FUS amplification of delivery when relying on in vivo or ex vivo organ fluorescence imaging characterization of drug delivery.

Other factors that could have contributed to relatively lower FUS enhancement in this study compared to other works include differential FUS pressures, tumor models, multi‐bolus MB‐FUS treatments, and potential of drug leakage. Tumor status, size, vascular architecture, and microenvironment influence nano supramolecular agent delivery and thus likely also affect microbubble shell biodistribution.^[^ ^2^, ^95^ ^]^ It is possible that alternative tumor models could yield higher shell delivery. For example, sites with privileged circulation (i.e., BBB intact intracranial tissue) often yield the greatest relative improvements to delivery, and this may contribute to why they are favored targets for drug‐loaded microbubble applications. In contrast, the 4T1 orthotopic tumor model elicited high passive Evans blue and microbubble shell accumulation. It is possible that microbubble composition plays a greater role in enabling differential FUS vessel response in models with more uniform vasculature architecture. However, as exemplified by Sierra et al.,^[^ ^96^ ^]^ even privileged intracranial sites can face limited FUS enhancement in microbubble shell delivery, requiring higher pressures (≥600 kPa) within inertial cavitation domains. We selected a peak negative pressure of 300 kPa to align with previous robust MB‐FUS co‐delivery and shell delivery studies and to avoid entering these inertial cavitation realms, often associated with vascular shutdown or hemorrhage.^[^ ^23^, ^97^, ^98^, ^99^, ^100^, ^101^ ^]^ It is possible that at higher pressures, like others,^[^ ^89^ ^]^ we too would observe greater FUS delivery enhancement of microbubble shells at tumor sites. Beyond FUS pressures, pulse interval can also affect the degree of reperfusion between pulses during sonication, and it is possible that the selection of a longer interval would have yielded more consistent overlap between the area exposed to ultrasound and populations of circulating microbubbles.^[^ ^23^ ^]^ Finally, while the presence of integrated feedback systems to control cavitation have become customary for cranial applications,^[^ ^102^, ^103^, ^104^ ^]^ their application for extracranial solid tumor delivery has the potential to greatly reduce variability and improve outcomes. Aside from adjusting FUS parameters, drug leakage from encapsulating/tethering microbubble shells may also contribute to these observed enhancements, which our study did not address. Beyond the limitations of our study, different starting tumor sizes, lack of appropriate controls, and unknown peak negative pressures in other studies could also contribute to observed differences.^[^ ^90^, ^91^, ^92^, ^93^, ^105^ ^]^

The perfusion studies also allowed for assessment of extravascular microbubble shell delivery and disruption in major organs. The hearts, lungs, and kidneys of perfused animals showed both lower accumulation of microbubble fragments (Figures S32 and S33D, Supporting Information) and lower fluorescence signal in the extravascular compartment (Figures S34 and S35D, Supporting Information) across almost all pDef treatment paradigms, supporting the idea from the PET analysis that signal within these organs is perfusion‐dominated. Accordingly, C18nPA pDef no longer exhibited higher heart accumulation in perfused mice, confirming that its higher PET signal was from higher blood concentrations. In contrast, the liver and spleen did not display any significant decrease in microbubble shell accumulation or fluorescence activation when comparing perfused to unperfused animals (Figures S32 and S33D, Supporting Information), suggesting that shell accumulation in these organs is more extravascular in nature. This allows for a more tailored interpretation of the hepatic and splenic trends gleaned from the radioimaging analysis: they are not only due to differences in perfusion between the different formulations, but rather differences in how these materials are taken up, retained, and processed by these organs. FUS did not significantly alter off‐target uptake of microbubble shells in any major organs (Figure S33, Supporting Information).

### 2.8 Summary of Findings and Recommendations

**Figure 8:**
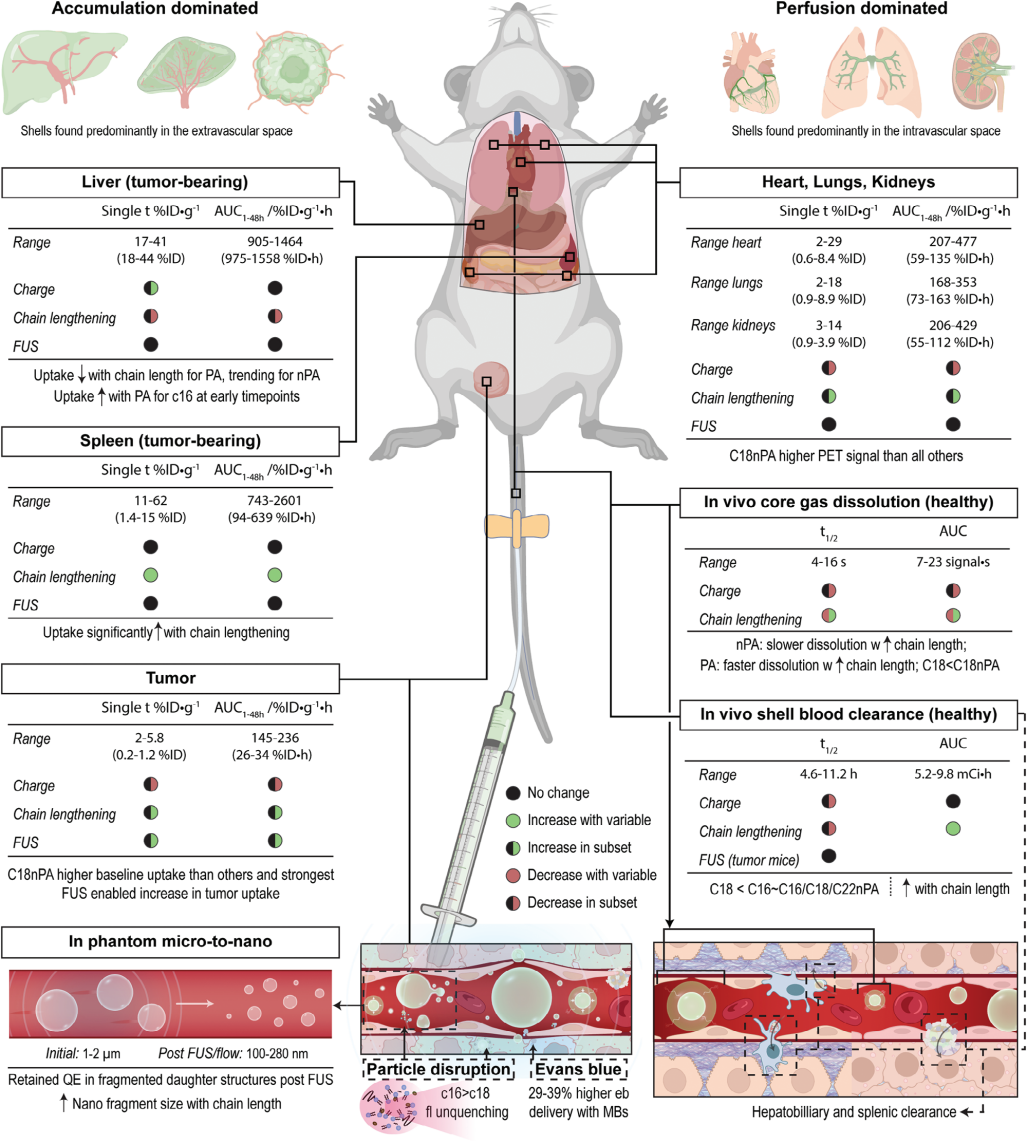
Summary of important pharmacokinetic principles about MB‐FUS platform design learned through quantitative, longitudinal evaluation using the pDef platform. While microbubbles exhibit ultrasound contrast only on the order of minutes, their shell components continue to circulate for hours to days. Microbubble shell chemistry affects multiple important pharmacokinetic parameters, including the size of circulating nano‐progeny following FUS exposure; degree of accumulation in retention‐dominated processing centers such as the liver and spleen; and overall extravascular deposition at a treated tumor site. Liver: Shorter chain lengths and negative charge increase uptake, particularly at early timepoints; Spleen: Longer chain lengths increase sustained uptake, charge has no effect; Tumor: C18nPA exhibits strongest FUS‐enabled increase in uptake; Heart/Lungs/Kidneys: C18nPA exhibits greatest PET signal, but pDef differences are equalized in perfused animals, showing that signal is perfusion‐dominated and extravascular deposition is low; In vivo core gas dissolution: Increasing chain length improves half‐life for neutral MBs, but lowers half‐life for negative MBs; In vivo shell blood clearance: Chain lengthening increases total blood pool exposure for neutral MBs; C18 had reduced half‐life compared to other pDefs; In phantom micro‐to‐nano: Nano fragment size post‐FUS increases with chain length while remaining fluorescently quenched; Evans blue: 29–39% higher extravascular delivery following MB‐FUS.

This study provides the first longitudinal, comprehensive analysis of microbubble core and shell pharmacokinetics across the entire microbubble shell circulation lifetime for four prevalently studied microbubble formulations. This was facilitated through the creation of a new, purification‐free, one‐pot microbubble radiolabeling method that allowed for efficient (>95% chelation efficiency) and stable (tested up to 1000 kPa sonication) chelation of [^64^Cu]Cu^2+^ to porphyrin‐lipid moieties included in the microbubble lipid shell. The chelation protocol did not perturb microbubble physicochemical properties and yet endowed the microbubbles with simultaneous PET and activatable fluorescence imaging capabilities previously unreported. The versatility of this radiolabeling protocol allowed us to accurately investigate the pharmacokinetics of 4 widely studied microbubble formulations analogous to the formulation of Definity, the most used commercial microbubble for drug delivery applications. This study consequently provides the first report of Definity analogue shell pharmacokinetics and is also the first study to systematically evaluate the effect of microbubble composition and FUS on shell fate. Such evaluation was conducted through complementary means for the first time: ultrasonography to quantify microbubble core dissolution, activatable fluorescence imaging to characterize particle disruption, and PET/γ‐ counting to quantify shell kinetic biodistribution and clearance. Collectively, this enabled the acquisition of new foundational knowledge pertinent to advancing MB‐FUS drug delivery paradigms. Below is a summary of these novel findings, which are summarized visually in **Figure** **8**.

Briefly, in vitro, C16 chain length microbubbles fragmented into smaller daughter fragments (100 nm) than C18 chain length microbubbles (200–280 nm) upon sonication. In vivo, we found that microbubble shells circulate for 24–48 h in healthy mice, yielding lipid composition‐dependent blood clearance half‐lives of 5 to 11 h, which were not significantly changed by tumor status or application of FUS. In healthy and 4T1 tumor‐bearing mice, microbubble shell fragments accumulated to the greatest extent in the liver and spleen (20–60% ID cc^−1^). Hepatosplenic uptake was rapid, yielding extravascular shell accumulation and disaggregation. In contrast, shell presence within the heart, lungs, and kidneys monotonically declined post‐injection, likely due to intravascular circulation of the shells in these organs rather than parenchymal deposition. Application of FUS did not affect the kinetic biodistribution of microbubble shells in these off‐target organs. Instead, microbubble charge and chain length played a great role in dictating off‐target shell biodistribution. Microbubbles composed of C16 chain length lipids yielded preferential uptake by the liver, while formulation with C18 chain length lipids caused preferential uptake by the spleen. The shorter chain length also facilitated stronger particle fluorescence unquenching (particle disruption) within these organs. Inclusion of an anionic charged PA moiety enhanced hepatosplenic uptake. Such clear trends were not observed for tumor delivery, whereby the C18 chain length neutral microbubble shell exhibited distinctly higher tumor shell delivery with or without FUS treatment than all other microbubbles. Microbubble shells of all compositions were found to passively accumulate to high degrees (up to 5% ID cc^−1^) within the 4T1 tumors even in the absence of FUS. This delivery was enhanced with FUS by a maximum of 50% at 3.5 h post‐injection and treatment. All microbubble shells showed extravascular deposition at the tumor site, with anionic C16 chain length microbubbles, the direct analogue of Definity, exhibiting the lowest extravascular delivery. At this early timepoint, neutral C16 and C18 chain length microbubbles exhibited similar levels of particle disruption, whereas at 48 h post‐injection, C16 chain length shells underwent 2–3‐fold higher disruption in the tumor than C18 chain length shells. The tumor featured the highest degree of fluorescence unquenching of all organs examined, suggesting that microbubble fragments undergo cellular uptake and disaggregation within the tumor.

#### 2.8.1 Addressing Common MB‐FUS (mis)Conceptions

The comprehensiveness of this study and its unique use of complementary in vitro and in vivo assays allowed us to verify some long‐held views on microbubble pharmacokinetics while challenging others. Consistent with literature, neutral microbubbles dissolved in circulation more rapidly with shorter lipid chain lengths.^[^ ^65^, ^66^ ^]^ A new finding in this study was that anionic microbubbles, like Definity, exhibit the opposite trend. Surprisingly, we could not find any systematic analysis of lipid chain length on anionic microbubble dissolution despite the inclusion of anionic PA groups in both FDA‐approved lipid microbubbles, Definity and Sonovue. A key finding in this study was that microbubble dissolution, typically presented as being synonymous with microbubble clearance,^[^ ^106^, ^107^, ^108^ ^]^ cannot exclusively be used to predict shell clearance, FUS activity, shell delivery, and compositional trends thereof. For example, although C18nPA pDef's longer acoustic stability could contribute to its stronger differential shell delivery post FUS, there was no corresponding decrease in FUS‐mediated delivery of C18 pDef, which exhibited the poorest acoustic stability. Thus, we urge the microbubble field to refrain from using microbubble gas imaging half‐lives to describe or infer overall microbubble circulation clearance and pharmacokinetics, particularly as applications involving shell‐mediated delivery increase in prevalence. Overall, it is unlikely that there exists a single causative property, such as acoustic stability, that is predictive of microbubble shell pharmacokinetics and FUS activity, necessitating the creation of a larger bank of structure‐activity relationships based on empirical acoustic and pharmacokinetic data.

With respect to kinetic biodistribution, our findings of hepatobiliary/fecal clearance of lipid microbubble shells in healthy and tumor‐bearing mice over 48 h align with prior studies conducted in healthy animals up to 1 h post‐injection.^[^ ^31^, ^109^ ^]^ However, unlike several studies, our work refutes the lungs and kidneys as primary sites of microbubble shell accumulation or clearance. Perhaps the most surprising and provocative literature biodistribution divergence in this study was for tumor shell delivery. Our phantom studies confirmed at a bulk level that C16 chain length microbubbles generate smaller nanofragments than C18 chain length microbubbles post FUS. However, this fragmentation difference did not lead to the postulated more rapid release of the shorter C16 fragments in vivo.^[^ ^73^ ^]^ Overall, FUS advantages for microbubble shell‐loaded drug delivery were lower than previously reported. This casts doubt regarding the universality of delivery advantages stipulated for drug conjugation to lipid microbubble shells^[^ ^110^ ^]^ across diverse targets. As a whole, FUS‐enabled drug delivery enhancements of lipid microbubble shell‐loaded agents is variable across the MB‐FUS literature. To this end, our results highlight the need for careful selection of tumor model, FUS parameters, and drug administration technique, while further advocating for the adoption of controlled, quantitative assays (see below) when designing and characterizing valuable MB‐FUS paradigms. This is particularly prudent given the growing interest in agent‐loaded microbubbles (Figure S1, Supporting Information).

#### 2.8.2 Strategic Design of All‐In‐One MBs

In addition to clarifying and expanding upon long‐assumed beliefs in the MB‐FUS delivery community, the findings of this study can directly yield structure‐activity relationships that enhance microbubble design and application, particularly for all‐in‐one, intrinsically theranostic MB‐FUS paradigms. For example, splenic targets may benefit from the use of C18 chain length microbubbles while C16 chain length microbubbles may be more suitable for hepatic applications; however, the ability of FUS to modulate delivery to these non‐tumor sites should be independently studied, as it may further enhance preferential delivery. For applications requiring higher blood exposure, neutral C18 microbubbles would be a superior choice to negatively charged and C16 chain length microbubbles. If stronger particle disruption is desired, C16 chain length microbubbles may be of interest. To augment tumor delivery efficacy, a direct Definity analogue may not be the best foundational composition to pursue. Our study suggests that in this regard, C18 neutral lipid microbubbles may be superior, but that shell‐loading of drug agents in general may not be as promising as previously thought for MB‐FUS delivery enhancement to extracranial solid tumors. The longitudinal characterization of off‐target shell accumulation can also inform more strategic design of drug‐microbubble selections. For example, the dose‐limiting cardiotoxicity of doxorubicin motivated the development of its liposomal formulation, Doxil, which reduced its myocardial accumulation.^[^ ^111^ ^]^ Analogously, our pharmacokinetic results demonstrate that shell conjugation of drugs with hepatosplenic toxicity should be avoided. If systemic blood exposure minimization is desired, C18 pDef‐like compositions may be more ideal.

As discussed above, there is a likely intersection of modulating FUS parameters, target tissue characteristics, and microbubble properties that we did not have resources to examine, but which would allow for more robust pharmacokinetic and pharmacodynamic libraries to be created and predictively used to purposefully enhance MB‐FUS therapies. This study provides adaptable techniques for obtaining such libraries. The versatile one‐pot microbubble radiolabeling technique advances microbubble pharmacokinetic data acquisition leaps beyond what was previously feasible. It can be applied efficiently across diverse formulations without major perturbations of the microbubble synthesis process and without concerns of chelation instability or associated free radioisotope release, all of which can cumulatively and individually skew biodistribution quantification.^[^ ^109^ ^]^ The study also demonstrates the limitations and utility of fluorescence imaging in pharmacokinetic studies while providing a validated PET workflow and rationalized timeframes to evaluate FUS enhancements of drug delivery, especially pertinent to tumors featuring high passive uptake. We hope this framework can be applied in future microbubble pharmacokinetic studies and facilitate more rapid expansion of structure‐activity relationships.

#### 2.8.3 Optimizing and Informing Conventional MB‐FUS

Our study demonstrates that FUS can cause a composition‐dependent excess microbubble lipid shell deposition on‐target of up to 1% ID g^−1^ in the extravascular space (3.5 h post treatment, 1.5‐fold over baseline), and an overall excess of 37% ID cc^−1^ h over 48 h post treatment. This on‐target extravascular deposition and off‐target shell residency profile is likely not inconsequential to the safety and efficacy of conventional co‐delivery MB‐FUS paradigms. Given increasing immunogenicity concerns associated with lipid supramolecular agents,^[^ ^38^, ^39^, ^40^ ^]^ this deposition may not be permissible for some patient populations. To this end, microbubble formulations with reduced on‐target extravascular delivery but retained FUS enhancement of co‐administered agent delivery, such as C16 pDef, may be needed. While systemic exposure may be minimized with C18 pDef‐like compositions, the high and prolonged hepatosplenic accumulation of all lipid shell fragments observed in this study may be contraindicated in some patients, and this should be a consideration among other inclusion/exclusion criteria when recruiting for MB‐FUS clinical trials.

On the other hand, some evidence suggests that on‐target shell deposition may have beneficial consequences. As MB‐FUS protocols attempt to deliver bulkier cargo (including CRISPR/Cas9 nanoparticles and cellular therapies^[^ ^112^, ^113^ ^]^), repurposing commercial microbubbles formulations, like Definity, may fail to achieve clinical efficacy while maintaining an adequate safety profile. For example, large payload delivery may require stronger and harsher paradigms (i.e., higher microbubble and FUS dosing),^[^ ^114^, ^115^ ^]^ which risks incursion of sterile inflammatory responses. Microbubble composition and pharmacokinetic modulation may be the key to realizing MB‐FUS improvements, an idea gaining traction following studies demonstrating that differences in shell stiffness, interaction with biological membranes, and deposition within target cells are thought to contribute to shell lipid composition‐dependent FUS effects.^[^ ^41^, ^42^, ^116^ ^]^ Cumulatively, this suggests that clinical microbubble formulations can be improved upon, a thought supported by select research groups that have attempted to re‐optimize microbubbles for evolving therapeutic needs.^[^ ^117^, ^118^, ^119^ ^]^ Interestingly, these pre‐clinical formulations reported to outperform Definity resembled C18nPA pDefs, the microbubble from our study which featured the highest on‐target deposition. Though our study was limited in this capacity, future complementary studies employing microscopic techniques (e.g., imaging mass cytometry or confocal imaging) could establish a clearer connection between pharmacokinetics, microbubble‐bio interactions, and FUS delivery efficacy, which could allow for more rapid, pharmacokinetically‐driven optimization of microbubble formulations.

#### 2.8.4 Nanomedicine Delivery Implications

A major driver of MB‐FUS development was to enhance the delivery of nanoparticle agents across physiological barriers. As large agents, nanomedicines would highly benefit from pharmacokinetic‐driven structure‐activity relationships that improve MB‐FUS for co‐delivery and microbubble shell‐loaded nanoparticle delivery paradigms. As discussed, microbubbles also present a nanoparticle delivery opportunity through in situ micro‐to‐nano conversions. This study provides new quantitative pharmacokinetic insight on this delivery utility. We show that microbubbles of all compositions fragment into nano‐sized species. These fragments were found to behave in alignment with known nanomedicine properties, including blood circulation lifetimes, hepatobiliary clearance, relation of fragment size to hepatosplenic accumulation, degree of passive tumor uptake, and the correlation of this uptake to higher retained doses in the blood pool.^[^ ^62^, ^76^, ^120^, ^121^ ^]^ Accordingly, the associated trends can be added to the existing, but still limited, repertoire of lipid nanoparticle structure‐pharmacokinetic activity knowledge. In particular, structure‐activity relationships surrounding passive tumor uptake and preferential hepatic versus splenic uptake are currently of intrigue in the nanomedicine field.^[^ ^122^, ^123^ ^]^ Given their pharmacokinetic similarities, lessons from the nanomedicine field may also be conversely applied to the MB‐FUS field. For example, cell population specific analyses have shown nanoparticles to accumulate in mononuclear phagocyte system (MPS) cells, such as Kupffer cells and splenic macrophages.^[^ ^124^ ^]^ The hepatosplenic accumulation observed in this study and prior literature precedent for MPS cell‐specific uptake^[^ ^31^, ^125^, ^126^, ^127^, ^128^, ^129^, ^130^ ^]^ opens remediating strategies already developed in the nanomedicine field to elevate MB‐FUS drug delivery efficacy, such as pre‐dosing and vasculature normalization.^[^ ^2^, ^131^ ^]^ Complement binding studies conducted in the nanomedicine literature could also further our failed attempts at correlating protein binding in a predictive manner to microbubble shell pharmacokinetics.^[^ ^82^, ^132^ ^]^ Overall, it is hoped that future, more mechanistic pharmacokinetic studies conceived at the intersection of micro and nano drug delivery fields will lead to improvements in both MB‐FUS delivery and nanomedicine therapeutic potential.

## 3 Conclusion

By introducing a simple and translatable radiolabeling strategy, this study was the first‐of‐its‐kind to explore the influence of lipid microbubble composition on the longitudinal pharmacokinetics and biodistribution of a microbubble‐mediated focused ultrasound platform in tumour‐bearing mice. By systematically evaluating the influence of both chain length and inclusion of negatively charged phospholipids, it clearly illustrated that material composition can dictate shell fate across several important organs of clearance, including the liver, spleen, heart, and lungs, in a manner not clearly explained by differences in shell fragment circulation. In tumours, effective drug delivery was closely tied to microbubble composition, highlighting that a better understanding of the biological behaviors faced by microbubbles in the context of focused ultrasound treatment is essential to their success as drug delivery systems. The study highlights the need for application‐driven microbubble composition optimization, appropriate target selection, and consideration of how these factors should impact FUS parameter selection. We hope the findings and framework of this study will serve as a step toward this ideal for the design of MB‐FUS delivery platforms and provide insights that allow for their smarter and more effective clinical implementation.

It is a crucial time for the field of MB‐FUS drug delivery to drive evidence‐based, rational agent design. The field is at a crossroads: will it plateau as the limitations of repurposing clinical microbubbles continue to surface, or will it move forward by embracing more careful, mechanism‐informed design? In this regard, lessons can be learned from its sister field of nanomedicine. Early systematic pharmacokinetic characterization allowed for successful translation of nanomedicines like Onpattro and Doxil. However, the field also suffered translational stagnation in the absence of continued robust biological characterization, lasting decades before this issue was coherently acknowledged by the community. This fate must be avoided for the MB‐FUS field. Momentum must be sustained by advancing the field in an informed manner to reach its immense potential in transforming the landscape of molecular and nanomedicine delivery. The current study is one cog on this wheel, and we hope it reinforces a framework that will keep the wheel turning and accelerating.

## Conflict of Interest

The authors declare no conflicts of interest.

## Author Contributions

M.A.R. and A.D. contributed equally to this work and shared first authorship. M.A.R., A.D., M.Z., V.C., M.O., J.W.L., and C.P. contributed to planning and conducting experimental work as well as reviewing the manuscript. Manuscript writing was conducted by M.A.R. and A.D. All work was guided under the supervision of D.G., J.C., and G.Z. All authors have given approval for the final version of the manuscript. All correspondence to G.Z.

## Supporting information

Supporting Information

## Data Availability

The data that support the findings of this study are available from the corresponding author upon reasonable request.
