# Supplementary material for: Quantitative Pharmacokinetics Reveal Impact of Lipid Composition on Microbubble and Nanoprogeny Shell Fate
Source: Adv Sci (Weinh). 2023 Nov 30;11(4):2304453. doi: 10.1002/advs.202304453 (PMC10811482; doi:10.1002/advs.202304453)
Supplement: Supplementary file 1 — Supporting Information [file ADVS-11-2304453-s001.pdf]

ADVANCED SCIENCE
Open Access

# Supporting Information

for *Adv. Sci.*, DOI 10.1002/advs.202304453

Quantitative Pharmacokinetics Reveal Impact of Lipid Composition on Microbubble and Nanoprogeny Shell Fate

*Maneesha A. Rajora, Alexander Dhaliwal, Mark Zheng, Victor Choi, Marta Overchuk, Jenny W. H. Lou, Carly Pellow, David Goertz, Juan Chen and Gang Zheng**

# Supporting Information

Quantitative Pharmacokinetics Reveal Impact of Lipid Composition on Microbubble and Nanoprogeny Shell Fate

*Maneesha A. Rajora#, Alexander Dhaliwal#, Mark Zheng, Victor Choi, Marta Overchuk, Jenny W. Lou, Carly Pellow, David Goertz, Juan Chen, and Gang Zheng**

## S1. Materials and methods

### S1.1 Materials

All lipids were purchased from Sigma-Aldrich Canada, with exception of pyropheophorbide-a-lipid, which was synthesized in-house as previously described.[1] Phosphate buffered saline (PBS), glycerol, propylene glycol, ammonium acetate, sodium hydroxide, copper (II) chloride, and trypsin-EDTA (2.5 g porcine trypsin and 0.2 g EDTA 4Na) were purchased from Sigma Aldrich. Perfluoropropane gas (PFP) was purchased from FluoroMed (Texas, USA). The **[**$^{64}$Cu**]**$CuCl_2$ radioisotope was generated at the Washington University School of Medicine Cyclotron Facility Syringe filters were purchased from Millipore (Billerica, MA). Hydrochloric acid and nitric acid for ICP-MS was purchased from VWR Chemicals BDH®. Methanol was purchased from Fisher Chemical (Toronto, CA). EDTA was purchased from Bioshop Burlington, Canada). Fluorescence-reduced rodent diet was purchased from Inotiv (Teklad® Custom Diets, TD.97184, Wisconsin, USA).

### S1.2 Literature review

Scopus was used to identify and categorize literature specific to lipid microbubbles. Three searches were combined with the following terms: 1) ( ( TITLE-ABS-KEY ( microbubble* ) OR TITLE-ABS-KEY ( ultrasound OR ultrason* AND contrast ) ) AND TITLE-ABS-KEY ( lipid* OR phospholipid* OR lecithin* OR ( fatty PRE/0 acid* ) OR phosphatidyl* OR acyl ) ) OR TITLE-ABS-KEY ( definity OR luminity OR sonovue OR lumason OR sonazoid OR imagent OR perflexane OR imavist OR br14 OR br38 OR ( mrx-408 ) OR micromarker ) AND ( EXCLUDE ( DOCTYPE , "re" ) ) AND ( LIMIT-TO ( LANGUAGE , "English" ) ); 2) TITLE-ABS-KEY ( focus* PRE/0 ultrasound ) AND TITLE-ABS-KEY ( microbubbl* ) AND ( EXCLUDE ( DOCTYPE , "re" ) ) AND ( LIMIT-TO ( LANGUAGE , "English" ) ); 3) TITLE-ABS-KEY ( pharmacokinetic* OR biodistribut* OR ( shell PRE/0 fate ) OR radiolabel* ) AND TITLE-ABS-KEY ( microbubbl* ) AND ( EXCLUDE ( DOCTYPE , "re" ) ) AND ( LIMIT-TO ( LANGUAGE , "English" ) ). This yielded a total of 8449 search results until the year 2021, of which 5842 were included in Figure S1 and categorized. The remaining 2607 results comprised review articles, inaccessible articles, duplicates, or articles not relevant to lipid microbubbles. MB-FUS clinical trials were identified on clinicaltrails.gov (most recent search on June 27, 2023) and categorized by microbubble type.

### S1.3 Microbubble Fabrication

A library of microbubble formulations inspired by the formulation strategy of Definity®[2] was developed using the protocol described by Huynh et al.[3]. The baseline Definity® formulation

was constructed by dissolving1,2-dipalmitoyl-sn-glycero-3-phosphocholine (DPPC) and 1,2-dipalmitoyl-sn-glycero-3-phosphoethanolamine-N-[methoxy(polyethylene glycol)-5000] (DPPEmPEG5000) in a 9:1 chloroform : methanol (v/v) solution with 1,2-dipalmitoyl-sn-glycero-3-phosphate (DPPA) dissolved in a 65:35:8 solution of chloroform : methanol : water (v/v) in a glass vial, generating lipid solutions containing a 82:8:10 molar ratio of DPPC:DPPE-mPEG5000:DPPA and a total lipid amount of 666 nmol. Porphyrin-lipid optimization was conducted by replacing the DPPC with increasing molar quantities of c16 pyropheophorbide-a-lipid (0, 1, 5, 10, 20, 25, 30, 40, or 50 mol% of total lipid). The same was conducted with c18 chain length Definity analogues, whose base formulation consisted of 1,2-distearoyl-sn-glycero-3-phosphocholine (DSPC), 1,2-distearoyl-sn-glycero-3-phosphoethanolamine-N-[methoxy(polyethylene glycol)-5000] (ammonium salt) (DSPE-mPEG5000), and 1,2-distearoyl-sn-glycero-3-phosphate (sodium salt) (DSPA) in a 82:8:10 molar ratio. A final 30 mol% total lipid was selected for the porphyrin-lipid quantity for subsequent experiments. The molar ratios within each of the pDefs are summarized in Table S1, and the quantities used to make their respective lipid mixtures were as follows:

<u>C16 pDef:</u> 0.254 mg (346 nmol) DPPC in 9:1 chloroform : methanol (v/v), 0.202 mg (200 nmol) c16 pyropheophorbide-a-lipid in chloroform, 0.304 mg (52.9 nmol) DPPE-mPEG 5000 in 9:1 chloroform : methanol (v/v), and 0.045 mg (67.1 nmol) DPPA in 65:35:8 chloroform : methanol : water (v/v). This latter solution is best made by adding methanol to water dropwise, followed by dropwise addition of chloroform. C16 pDef formulations made with 1 and 10 mol% porphyrin-lipid had the same DPPA and DPPE-mPEG 5000 compositions but altered DPPC and c16 phyropheophorbide-a-lipid compositions: 1 mol% porphyrin C16 pDef contained 0.396 mg (540 nmol) DPPC and 7 µg (6.66 nmol) c16 phyropheophorbide-a-lipid while 10 mol% porphyrin C16 pDef contained 0.352 mg (480 nmol) DPPC and 0.067 mg (66.6 nmol) c16 phyropheophorbide-a-lipid.

<u>C16nPA pDef</u>: 0.304 mg (413 nmol) DPPC in chloroform : methanol (v/v), 0.202 mg (200 nmol) c16 pyropheophorbide-a-lipid in chloroform, and 0.304 mg (52.9 nmol) DPPE-mPEG 5000 in 9:1 chloroform : methanol (v/v).

<u>C18 pDef</u>: 0.274 mg (346 nmol) DSPC in 9:1 chloroform : methanol (v/v), 0.208 mg (200 nmol) c18 pyropheophorbide-a-lipid in chloroform, 0.307 mg (52.9 nmol) DSPE-mPEG 5000 in 9:1 chloroform : methanol (v/v), and 0.049 mg (67.1 nmol) DSPA in 65:35:8 chloroform : methanol : water (v/v).

<u>C18nPA pDef</u>: 0.327 mg (413 nmol) DSPC in 9:1 chloroform : methanol (v/v), 0.208 mg (200 nmol) c18 pyropheophorbide-a-lipid in chloroform, and 0.307 mg (52.9 nmol) DSPE-mPEG 5000 in 9:1 chloroform : methanol (v/v).

<u>C22 nPA pDef</u>: 0.373 mg (413 nmol) 1,2-dibehenoyl-sn-glycero-3-phosphocholine (DBPC) in chloroform, 0.219 mg (200 nmol) c22 pyropheophorbide-a-lipid in chloroform, and 0.307 mg (52.9 nmol) DSPE-mPEG 5000 in 9:1 chloroform : methanol (v/v).

The solutions were evaporated under nitrogen gas flow followed by storage under vacuum for a minimum of 12 hours. The dried films were rehydrated with 1 mL of excipient (80:10:10 v/v/v of phosphate buffered saline (PBS), glycerol, and propylene glycol, referred to as PGG) and the headspace was replaced with perfluoropropane gas (PFP). The hydrated films were heated to 70ºC and sonicated using a bath sonicator (Branson Ultrasonics, Rochester, USA) to disperse the lipids and create a homogeneous suspension. This was followed by an additional purging with PFP before final sealing with parafilm. Suspensions were stored at 4ºC for up to

two months. Before usage, solutions were brought to room temperature passively before mechanical activation for 45 seconds using a VialMix® (Lantheus Medical Imaging Inc.). The activated solutions were allowed to cool to room temperature for 10 minutes prior to a gentle re-suspension via hand inversion and reversion of the vial. After waiting 2 minutes to allow for passive buoyancy-based size selection of microbubbles, a maximum of 550 µL of solution was extracted from the bottom of the vial using a 1mL syringe with an 18 G needle and transferred to a sterile 1.5 mL Eppendorf tube after the needle was wiped to remove any coated microbubble foam. Samples were analyzed and utilized within 90 minutes of activation to minimize variability from changes in microbubble populations over time.

S1.4 Microbubble copper chelation

A hybrid post labeling chelation[4] protocol was developed. Lipid films were created and rehydrated as described above, but the excipient was substituted with 1 mL of syringe filtered (0.22 µm cutoff) 5:3:1:1 0.1 M $NH_4OAc$ (pH = 5.5) : PBS : propylene glycol : glycerol (termed AA-PGG). For 1% and 10% porphyrin-lipid formulations, the films were hydrated with 0.15 mL AA-PGG and were topped to a final volume of 1 mL post chelation. After creating the lipid suspension, “cold” $CuCl_2$ (2 nmol, 2.69 µL of a 0.1 mg·mL$^{-1}$ $CuCl_2$ solution in 0.1 N hydrochloric acid) or [$^{64}$Cu]$CuCl_2$ (5-6 mCi in 0.1 N hydrochloric acid, variable concentration provided but less than 10 µL volume maximum addition) was added. The vial was topped with PFP, capped, and sealed with parafilm before heating at 60ºC for 1 hour. The reaction mixture was sampled by instant thin layer chromatography (iTLC) to confirm successful (>95%) chelation purity. The vial was allowed to cool to room temperature during this time. Once successful chelation was confirmed, the lipid suspension was neutralized with 1 N sodium hydroxide (8.89 µL). The vial was inverted/reverted to mix the contents gently without creating any bubbles. At all steps, care was taken to ensure the vial remained capped to prevent PFP de-saturation. The vial was then topped with PFP one last time, capped, and sealed with parafilm before activation with a VialMix® to generate microbubbles. After 10 minutes, the resulting microbubble suspension was decanted as described above. Chelated microbubbles were used within 90 minutes of activation.

Copper chelation efficiency was evaluated through a validated centrifugation protocol. Microbubble samples (200 µL) were added to 30,000 MWCO 0.5 Amicon® centrifugal filter units. The unit was spun at 12,000 rcf for 10 min. It was transferred to a new tube and two additional washes were conducted by replenishing the supernatant with 200 µL dd$H_2O$ and centrifuging at 12,000 rcf for 10 min (unit was changed to a fresh tube for each wash). The resulting supernatant (containing porphyrin-chelated copper) in the unit and the infranatant tubes (containing free copper) were subjected to γ-counting (Wizard 1480, PerkinElmer Inc). Counts-per-minute (cpm) were baseline corrected with an empty tube and chelation efficiency was calculated as follows:

$$efficiency\ \% = \left(\frac{Corrected\ cpm\ supranatant\ unit}{Corrected\ cpm\ supranatant\ unit + infranatant\ tubes}\right) \times 100\% \qquad (1)$$

This method for separating chelated and free copper was validated using an aqueous AA-PGG solution of “cold” $CuCl_2$, which was processed in the Amicon® unit as described. It was found to completely remove free copper from the supernatant unit and recovered the copper in full in the infranatant (Figure S8) following centrifugal washing as quantified by inductively coupled plasma mass spectrometry (ICP-MS, Perkin Elmer NexION 300Q) following digestion in concentrated nitric acid and dilution in 2% nitric acid mobile. Samples were run against a standard curve prepared from a known atomic microscopy standard of Cu in 2%

nitric acid (PerkinElmer Pure Plus). Radiochemical purity was determined from iTLC and radio-HPLC as previously described.[5-6] Briefly, the chelation reaction mixture was spotted 1 cm from the bottom of a strip of heat-activated glass microfiber chromatography paper (Agilent Technologies). The strip was eluted with an aqueous mobile phase composed of 2 v/v% EDTA, 10 v/v% 0.1 M $NH_4OAc$. Following elution, the developed strip was sectioned into thirds: 1) bottom spot containing supramolecular chelated copper, 2) middle section containing free [$^{64}$Cu]Cu-porphyrin-lipid and free copper streak, and 3) free Copper-64. The strip sections were γ-counted and purity was calculated:

$$purity\ \% = \left(\frac{Corrected\ cpm\ bottom\ strip}{Corrected\ cpm\ all\ strip\ sections}\right) \times 100\% \quad (2)$$

Radio-HPLC was conducted on a Waters ACQUITY UPLC® BEH C18 column (130 Å, 1.7 µm, 2.1 mm × 50 mm) with a flow rate of 0.6 $mL{\cdot}min^{-1}$ at 60 °C. A gradual solvent gradient was used, starting from acetonitrile/0.1% trifluoroacetic acid in a ratio of 20:80 to 0:100 over 7 minutes. The ratio was then held steady for 2 minutes before sharply decreasing back to 20:80 and held for another 2 minutes. The elution of $^{64}$Cu-labeled porphyrin-lipid (particles were disrupted during HPLC running) occurred around 7.0-7.6 minutes, while free Copper-64 eluted at 0.6 minutes. The corresponding absoption peaks of porphyrin-lipid around 7.0-7.6 minutes were detected using a 400 nm absorption channel.

S1.5 Microbubble physicochemical characterization

Microbubble size distributions were measured with a Coulter Counter (Multisizer 4e, Beckman Coulter). Microbubbles (2-5 µL) were diluted into 10 mL of Isoton II electrolyte solution (Beckman Coulter) pre-filtered through a 0.2 µm Nalgene filter unit (Thermo Scientific) and measured through a 30 µm aperture (0.6–18 µm size range). Raw counts were processed as both number-weighted and volume-weighted size distributions, and population statistics such as microbubble concentration and mean size were calculated in the Multisizer 4.03 software. Additional metrics of interest, such as population fractions above 8 µm, position of volume distribution peaks, and integrated gas volumes, were calculated in Matlab®. Microbubble morphology and porphyrin-lipid distribution in the shell were evaluated with confocal microscopy (SP8, DM18-CS Leica). A 63X HC PL APO CS2 Oil Immersion objective (NA=1.4), an OPSL 552 laser for variable wavelength excitation, and a PMT Trans detector were used. Spectral fluorescence measures were conducted using a FluoroMax®-4 Fluorescence Spectrometer for both intact microbubbles (1:2000 dilution into PBS) and fully disrupted microbubbles (1:2000 dilution in 1% v/v TritonX-100/PBS) using an excitation wavelength of 410 nm and emission range of 600 – 800 nm. All measures were normalized by voltage fluctuations in the instrument. Quenching efficiency of the samples was determined by comparing integrated emission data of intact versus disrupted microbubbles using the following relationship:

$$QE = (1 - \left(\frac{\int_{600\ nm}^{800\ nm} F_{PBS}}{\int_{600\ nm}^{800\ nm} F_{Tx}}\right)) \times 100\% \quad (3)$$

UV-Vis spectra (Cary 60 UV-Vis-NIR Spectrophotometer, Agilent) were acquired from 200-800 nm at a scanning interval of 0.25 nm on disrupted samples (10x dilution in methanol) and intact samples (10x dilution of bath sonicated microbubble suspensions to remove scattering gas in PBS). Molar emissivity was calculated as previously described.[7] Circular dichroism

spectroscopy (Jasco J-185) was conducted on the sonicated intact samples at 20x dilution in PBS at 25ºC.

The effective surface charge of microbubbles was estimated through electrophoretic light scattering measurements of processed microbubbles (Zetasizer Nano, Malvern Instuments, UK). To eliminate the confounding buoyancy of microbubbles, activated and decanted microbubbles were bath sonicated (Branson) until transparent. To remove the ionic excipient (PGG), the samples were spun at 4000 g for 30 min through a 10,000 MWCO filter (Amicon-4) a total of 3 times, interspersed with 3 mL washing steps using dd$H_2O$, analyzing the intranatant after each centrifugation via UV-Vis spectroscopy to ensure no loss of lipid. Samples were further diluted in dd$H_2O$ and at 25°C. Five measurements were conducted per sample, averaged to represent a single experimental replicate.

S1.6 In-phantom Cu-pDef sonication

Activated "cold" Cu-pDef suspensions were diluted 14x into PBS to represent the approximate concentration in murine blood. This diluted suspension was flowed through a vessel phantom made of 2% agar at a flow rate of 2 $mm \cdot s^{-1}$ (12.6 $mL \cdot h^{-1}$ in a 1.5mm cylindrical flow tube). The phantom was exposed to focused ultrasound (Videoscan immersion transducer, Olympus; 1 MHz, 1 s pulse length, 1% duty cycle, effective pulse length of 10 ms) at peak negative pressures of 0, 300 kPa, or 1000 kPa. Following flow ± FUS, samples were collected and aliquoted to evaluate copper chelation stability, size, and fluorescence. All runs and subsequent characterization were conducted in triplicate.

**Copper chelation stability**: 400 µL was serially centrifuged in 0.5 Amicon® centrifugation units at 12,000 rcf, including two washes. The resulting supernatant was collected and the unit was washed into an Eppendorf tube, which underwent vacuum centrifugation overnight (Savant SPD131DDA and RVT5105). The following day, the resulting cake was dissolved in a known quantity of methanol, and an aliquot was removed and analyzed by UV-Vis spectroscopy to assess porphyrin-lipid concentration. The remaining solution was dried by speed vacuum again overnight. It was digested in concentrated nitric acid at 60ºC for two hours, followed by dilution into 3 mL of 2% nitric acid. These samples were analyzed using ICP-MS (Perkin Elmer NexION 300Q) against a standard curve prepared from a known atomic microscopy standard of Cu in 2% nitric acid (PerkinElmer Pure Plus). A 500 µL injection loop was used in which the sample solution was mixed with a carrier (2% nitric acid) and an internal standard (Ir, 20 ppb) during automated sample aspiration. A minimum of two rinses were conducted between sample analysis to minimize cross-contamination.

**Fragment sizing**: Dynamic light scattering (DLS) (Zetasizer Nano SZ Particle Size Analyzer, Malvern) was performed to analyze daughter structures following flow and sonication. The diluted pre-flow microbubble suspensions or post FUS/flow samples were analyzed in a small volume (50 µL) ZEN0118 disposable cuvette. Six acquisitions were conducted on each sample with pre and post equilibrations of 3 and 5 s respectively. The suspensions were mixed by pipetting gently to redistribute buoyant particles in between each acquisition. An average of these six acquisitions represented one experimental replicate.

**Quenching efficiency**: Samples (0.5 µL) were diluted into 2 mL of either PBS or 1% v/v Triton X-100/PBS, after which 200 µL was added to black 96-well plates (Corning™ Costar™). Fluorescence spectra (excitation 410 nm, emission 600–800 nm) of all samples were acquired on a CLARIOstar microplate reader (BMG LABTECH) at room temperature.

Following blank subtraction, spectral intensities were integrated, and quenching efficiency was calculated according to Equation 3.

S1.7 In solution microbubble serum interactions

**J-aggregate assay**: Microbubble 702 nm absorbance Q-band (associated with stable ordered J-aggregation of porphyrin-lipid in supramolecular structures) was monitored ratiometrically alongside the 674 nm absorbance band (associated with loss of this ordered aggregation) as a readout for serum binding and supramolecular instability. The pre-flow 14x dilute samples from Section 5.6 were used. 100 µL of sample was combined with either 100 µL sterile PBS or fetal bovine serum (FBS, Wisent Bio Products, Canada) in 96-well plates (Sarstedt). The plates were scanned by CLARIOstar microplate reader continuously at 2-minute increments to acquire kinetic absorbance spectra from 600–800 nm over 12 hours at 37°C. The plate was orbitally shaken at 500 rpm for 10 s before each cycle. Spectra were baseline subtracted using PBS or 50% v/v FBS/PBS within the well plates. Experiments were conducted in triplicate.

**BCA assay**: Differential non-specific binding of various microbubble formulations to proteins was assessed using a Pierce™ Bicinchoninic Acid (BCA) Protein Assay Kit (ThermoScientific, USA). Microbubbles were incubated with FBS at a 2:3 ratio for 5 minutes at 37ºC with 200 rpm agitation (Multitherm Shaker, Benchmark Scientific). Microbubbles were isolated from unbound protein via centrifugation (100G, 2 minutes) twice, discarding the infranatant and reconstituting the microbubble "cake" each run in sterile PGG. The retained protein-bound microbubbles were destroyed using a bath sonicator to prevent scatter during absorbance-based measurements. These samples were diluted by a factor of 4 and processed using the BCA Assay according to the provided protocol.[8] In brief, the samples were mixed with freshly prepared Working Reagent at a ratio of 1:20 and incubated for 30 minutes at 37ºC. After cooling for 15 minutes, samples were analyzed using UV-Vis spectrophotometry. The signal at 562 nm was blank-subtracted and analyzed in reference to a concentration curve constructed using the Working Reagent and an albumin standard to calculate the amount of retained protein in the isolated microbubble fraction.

S1.8 Animal models

All animal experiments were conducted in compliance with the University Health Network Animal Care Committee guidelines and requirements (AUP 4299). Healthy, female BALB/c mice (6-7 weeks of age, 20 g) were used for acoustic kinetic, blood clearance, and PET validation studies. A 4T1 orthotopic tumor model was used for all other in vivo studies. The 4T1 murine mammary carcinoma cell line (provided in kind by Dr. Warren Chan, University of Toronto) was cultured in RPMI1640 media (Thermo Fisher Scientific, Waltham, USA) supplemented with 10% v/v FBS (Invitrogen, Carlsbad, US) and 1% v/v penicillin/streptomycin (Biofluids, Camarillo, USA)) that was exchanged every 48 hours. Cell culture conditions were maintained at 37ºC under 5% $CO_2$. Cells were harvested at 50% confluence using 0.25% Trypsin-EDTA for 2 minutes at 37ºC to dissociate cells from their plate. The trypsinized mixture was deactivated through 4-fold dilution into fresh RPMI1640 medium, and cell concentration was measured through counting on a hemocytometer. Cells were temporarily stored at a concentration of $1x10^6$ cells·$mL^{-1}$ prior to inoculation. Mice were placed under gaseous anesthesia (2% v/v isoflurane in oxygen) and injected with $1x10^5$ 4T1 cells in 100 µL serum-free media orthotopically at the right inguinal mammary gland using a 1 mL syringe fitted with a 25 G needle.[9] After inoculation, tumor growth was monitored every two days using digital calipers. Experiments were performed on day 12–14 after tumor inoculation when tumors reached approximately 8 ± 2 mm in the longest dimension. Tumor-

bearing mice were then separated into cohorts with the aim of size matching tumors across experimental arms for each session. All tumor dimensions (width, length, volume) and weights across all experimental arms are shown in Figure S36, while white light images of excised tumors (taken via Maestro II, CRi) are provided with scale bars in Figure S37.

S1.9 Microbubble acoustic kinetics

In vivo acoustic persistence of circulating microbubbles of varying formulations was measured using diagnostic ultrasound in non-linear contrast mode (EPIQ7G, Philips). Healthy BALB/c mice were anesthetized via injection of ketamine/xylazine and 26 G tail vein catheters (Abbocath-T) were inserted. They were positioned in a tank of degassed water held at 37ºC. An L12-5 array transducer was oriented such that the left kidney and renal artery were in the imaging field of view, capturing a coronal bisection of the kidney. Baseline images were taken in both B-mode and non-linear contrast mode. A 50 µL bolus of microbubbles (diluted to achieve an in vivo concentration of $5x10^6$ MB/mL) was injected, followed by a 100 µL saline flush, and images were recorded at a frame rate of 6 fps for 5 minutes. Images were acquired at identical settings for all formulations (MI: 0.07, 2D gain: 42%, zoom: 3.4, frequency: 12 MHz), and the system's log compression was removed for data export such that videos could be processed in linear intensity and appropriately quantified.

Regions of interest around the renal cortex, full kidney, and the renal artery were defined, and signal intensity was extracted over time across the collected frames to calculate mean transit time (elapsed time for which signal is above 50% of the signal maximum), total transit time (elapsed time for which signal is above 10% of signal maximum), rising time (elapsed time between noise floor and signal maximum), falling time (elapsed time between signal maximum and return to noise floor). Perfusion curves were modeled using a one-phase decay model in GraphPad Prism, normalized to the highest signal intensity and plateauing to background, to yield microbubble dissolution rates and half-lives. Perfusion curves were also modeled by a gamma-variate function and a non-linear least squares method in Matlab® using Equation (4).

$$I_{max}\left(\left(\frac{t}{t_{max}}\right)^{t_{max}*\beta} * e^{-\beta(t-t_{max})}\right) \tag{4}$$

Here, t is time, $I_{max}$ is the maximum intensity of the curve, $t_{max}$ is the time post-injection at which the maximum intensity is reached, and ß describes the filling and emptying kinetics of the contrast agent.[10-12]

S1.10 Blood clearance pharmacokinetics

Shell fragment circulation was assessed via serial blood sampling over 48 hours following injection. Healthy, female BALB/c mice were administered 200 µL of diluted $^{64}$Cu-chelated pDefs (1:1 [$^{64}$Cu]Cu-pDef:saline v/v) through the tail vein using 1 mL Norm-Ject syringes equipped with 26 G needles. Approximately 20 uL of blood from the femoral vein was collected at each timepoint (5 min, 30 min, 1 h, 2 h, 4 h, 8 h, 24 h and 48 h) using pre-weighed, heparinized microhematocrit capillary tubes (Fisher Scientific) and placed in 20 mL scintillation vials. The vials containing the tubes were capped, weighed, and subjected to γ-counting (Wizard 1480, PerkinElmer Inc, linear detection limit for Copper-64 of 1.88 nCi and a 60 second integration time). The cpm were baseline subtracted and normalized to the 5 min injection time point. The resulting clearance profiles were fitted in GraphPad Prism to a two

phase decay model (t-test determined that two-phase exponential fits outperformed a one-phase exponential fit, $p < 0.05$) to obtain half-lives and associated 95% confidence intervals. Area under the curve (AUC) were also obtained through GraphPad Prism.

S1.11 Focused ultrasound delivery system

A custom focused ultrasound exposure system was constructed using design principles from the Focused Ultrasound Group at Sunnybrook Research Institute,[13] producing a system capable of precise control of pressure, frequency, pulse length, duty cycle, interburst spacing, and other pertinent ultrasound parameters. In brief, signals are generated and controlled by two arbitrary function generators (Tektronix AFG 3021C and 3022C), amplified by 52 dB (Electronics & Innovation 2100L, 10 KHz – 12MHz, 100 W, Gating Option), and attenuated by 20 dB (BW -20N100W+ Fixed attenuator) before being sent to a transmit transducer (for in vivo experiments: Videoscan immersion transducer, 1 MHz, 3.81 cm element diameter, Olympus). Passive acoustic detection was performed using a receive transducer (Centrascan immersion transducer, 1 MHz, 2.54 cm element diameter, Olympus) oriented perpendicular to the transmit axis before being amplified by a 23 dB pre-amplifier (ABL0015-01-2317 low noise amplifier, Wenteq Microwave Corp) and digitized using an oscilloscope (PicoScope 5242D, 2-channel). Transducers were mounted on micropositioners (M-460P-XYZ, Newport) for precision control on three axes and fastened into an acrylic tank. Pulse triggering and recording was performed using custom scripts (Matlab®). Transducers were calibrated in this system using a 200 µm aperture needle hydrophone (HGL-0200, Onda, Sunnyvale, USA) to establish the driving voltages necessary to achieve the specific peak negative pressures explored in this study. A schematic of this setup is presented in Figure S38.

Mice were fastened to a custom holder that vertically secured the animal with the ventral side exposed. Animals were transferred into the acrylic tank containing the focused ultrasound delivery system that was filled with water which had been allowed to passively degas for 24 hours prior to experiments. Water temperature was maintained at 37ºC using a circulating water heater. The tumour of the mouse was co-localized to the focal volumes of both the transmit transducer and the receiver transducer using stereotactic fiducial markers corresponding to placement of a ball bearing reflector used during acoustic alignment of the transducers. Only tumors which would fit within the -6dB focal volume of the transmit transducer (9.6 ± 0.2 mm, as measured during hydrophone calibration for the aforementioned 1 MHz, 3.81 cm element diameter transmit transducer) were used to ensure complete tumor exposure to ultrasound. A single ultrasound pulse was delivered prior to injection to confirm orientation of the tumour with respect to the transducers and for normalization of passive acoustic detection data. Treatment was conducted at 1 MHz, 300 kPa, 1 s pulse length, 1% duty cycle (thus, the effective "on" pulse length was 10 ms), 2 minute total sonication time. These parameters align with studies demonstrating successful extravascular deposition of lipid microbubble shells following FUS [14-15] and are similar to low-intensity FUS studies conducted by other groups. [16-17] Additionally, this choice attempts to avoid any potential sonodynamic effects of porphyrin-lipid which may increase in likelihood with increasing pressure, and which could influence pharmacokinetics and biodistribution.

Acquired passive acoustic detection data was processed in MATLAB® (MathWorks, Natick, MA). In brief, received signals were digitally filtered (0.5 MHz high-pass and 5 MHz low-pass; $5^{th}$ order bandpass Butterworth filter), multiplied by a 100 µs Hanning window, centered on the channel axis, and Fourier transformed. The magnitudes of the frequency spectra were squared to obtain power spectra, which were averaged across successive pulses and compared to pre-injection baseline acoustic signals on log-transformed plots. The presence of

microbubbles may be inferred from the emergence of subharmonic peaks up to the 3rd order (stable cavitation) in these power spectra, in addition to an increase in broadband frequency content primarily around the fundamental frequency. Quantitative analyses of these power spectra are inappropriate given the imprecise geometry of the treated tumors, leading to variable acoustic reflections which impact the absolute magnitude of received acoustic signal between animals.

S1.12 Biodistribution

Animal preparation: Studies using healthy BALB/c mice did not require mouse pre-preparation prior to experiments. All 4T1 tumor-bearing mice were placed on a fluorescence-reduced diet two days prior to experiments. Tumor-bearing mice used for PET/fluorescence studies were shaved with electronic trimmers followed by depilatory cream to remove fur from their backs and torsos for ease of viewing of tumors, clearer fields of view for hyperspectral fluorescence imaging, and improved transmission of focused ultrasound to the tumor target. Fur was removed only around the tumor region for tumor-bearing mice used for perfusion studies.

Injections and treatments: Anesthesia was induced using 5% v/v isoflurane and maintained at 2% v/v isoflurane for the duration of the setup and treatment procedure. Catheters (Terumo Surflo Winger Infusion Set, 27 G, Global Medical Solutions) were inserted into the tail vein and flushed with heparinized saline. The radioactivity of $^{64}$Cu-chelated pDefs was measured on a CRC®-15R Dose Calibrator (Capintec) prior to injection. Subsequently, the agent was injected through the implanted catheter at a rate of approximately 10 µL/second (105 µL [$^{64}$Cu]Cu-pDef diluted into a total of 200 µL saline, 0.3–0.6 mCi per mouse for all PET/fluorescence studies, 0.1-0.3 mCi per mouse for perfusion studies). Assuming a murine total blood volume of 1.7 mL,[18] this approximated an injected in vivo concentration of $5\text{x}10^6$ microbubbles·mL$^{-1}$. For 4T1 tumor-bearing animals exposed to FUS, sonication began immediately following the start of injection. Tumor-bearing animals not exposed to ultrasound underwent a sham procedure in which they remained in the heated tank following injection for the same length of time as ultrasound-treated mice (2 minutes). Animals were then removed from the tank, dried, and allowed to recover from anesthesia on a heating pad. Residual radioactivity in the syringes/catheters was measured on the dose calibrator to accurately account for total administered dose. All treatment groups comprised of at least 5 animals.

Imaging: Whole-body microCT/PET (either a combination of Locus Ultra, General Electric for CT & Inveon, Siemens for PET; or NanoScan, Mediso for combined PET/CT) was performed at 1 h, 3.5 h, 6 h, 24 h, and 48 h post-injection. Animals were induced with 5% isoflurane anaesthesia and fastened securely in a transferrable bed that was fitted to both systems to allow for optimal data volume co-registration. PET acquisitions were acquired under 1–2% isoflurane anaesthesia at 1, 3.5, and 6 h used a PET imaging duration of 15 min; 24 h used a duration of 30 min; and 48 h used a duration of 60 min. Slices were reconstructed into three-dimensional whole-animal volumes with attenuation compensation from the paired microCT acquisition.

Hyperspectral fluorescence imaging (Maestro II, CRi) was performed on 4T1 tumor-bearing mice under 1–2% isoflurane anaesthesia prior to injection (pre-scan) and at 1 h, 3.5 h, 6 h, 24 h, and 48 h post-injection either directly before or directly after PET/CT imaging. Image cubes were acquired under both white light (100 ms exposure) and using a red filter set (635 nm (616-661 nm) excitation. Red light exposures of 250, 500, and 750 ms were trialed, and

500 ms was selected for in vivo imaging for its optimal dynamic range without any overexposure.

Euthanasia: Animals used for blood clearance and PET studies were euthanized at 48 h post-injection after the final blood collection or imaging via cervical dislocation under surgical plane anaesthesia (5% isoflurane). Animals used for perfusion studies were euthanized 3.5 h post-injection through: 1) cervical dislocation under surgical plane anaesthesia (5% isoflurane) for the unperfused animals, or 2) full body perfusion to clear residual blood for perfused animals. In this latter subset, animals were kept under 5% isoflurane. The chest wall was removed to expose the heart. A 27 G needle hooked to a peristaltic pump was inserted into the left ventricle, and a cut was made in the right atrium. PBS was perfused through the pump at a flow rate of 2.7 $mL \cdot min^{-1}$ for 7.4 min (total of 20mL), followed by 5.4 $mL \cdot min^{-1}$ for 2.53 min (total of 20mL), and finally by 11.8 $mL \cdot min^{-1}$ for 50 s (total of 10 mL). Indications of a successful perfusion included a blanched heart, brain, lungs, and liver, as well as clear flow of saline by the end of the perfusion.

Ex vivo analysis: Following euthanasia, the following tissues were dissected across all animals: liver, spleen, kidneys, heart, lungs, brain, small intestine, large intestine, adrenal glands, gall bladder, feces, skin, and muscle. The tumor, ipsilateral inguinal mammary fat pad, and contralateral inguinal mammary fat pad were collected for tumor-bearing mice. Within 2 hours of extraction to prevent drying, organs were weighed in tared scintillation vials and sealed. All experimental group tissue (healthy and tumor-bearing) underwent end-point quantification of Copper-64 activity in dissected organs through γ-counting (Wizard 1480, PerkinElmer Inc, linear detection limit for Copper-64  of 1.88 nCi and a 60 second integration time). Decay correction was performed relative to the time of injection using a calibration curve generated by measuring linear ranges of Copper-64 solutions on the dose calibrator and γ-counter. For tumor-bearing animals, tissues were also subjected to ex vivo hyperspectral fluorescence imaging as per above but using a 250 ms red light exposure.

S1.13 Quantitative processing of imaging data

Whole-body microCT/PET data co-registration and organ contouring was performed in Inveon Research Workplace. Co-registration estimations provided by the program were manually adjusted to account for minor rotational differences between the PET and CT acquisition hardware. Full organ three-dimensional contours of the liver, spleen, kidneys, heart, lungs, and tumor were manually constructed for all animals and all timepoints, after which the PET-based contouring data for all organs was exported and processed in Matlab®.[19] PET signal intensity within organ contours was individually converted to %ID and $\%ID \cdot cc^{-1}$ using the measured administered radioactive dose and decay-corrected to the time of acquisition. Total organ exposure over 48 hours was estimated through area-under-curve (AUC) calculations of time series PET data using a trapezoidal integration method.

Fluorescence data underwent spectral unmixing using spectra generated from porphyrin-lipid solutions and autofluorescence spectra from untreated tissue. This allowed for better isolation of the fluorescence deriving from the porphyrin-lipid within the mouse from the remaining autofluorescence that could not be eliminated using a fluorescence-reduced diet. Unmixed spectral data was then thresholded and scaled for consistency between animals, and fluorescence images were overlaid onto white light images for visualization. Fluorescence signal was quantified in ImageJ using ROI analysis.

S1.14 Evans Blue assay

Evans Blue dye (Sigma-Aldrich) was diluted in heparinized saline at 0.5% w/v and filtered through a 0.2 µm filter. Microbubble injection and focused ultrasound sonication procedures described in 5.11 and 5.12 were performed in tumor-bearing mice, after which 100 µL of the Evans blue solution was injected via the same indwelling tail vein catheter followed by a saline flush. After 2 minutes, animals were removed from the tank and allowed to recover. 3.5 hours after injection, mice were sacrificed via transcardial perfusion and dissected. Tumors were imaged using hyperspectral fluorescence imaging (Maestro II, CRi, yellow filter 595 nm (576–621 nm), 635 nm long-pass emission, 25 ms exposure). Unmixing was conducted using spectra obtained for untreated ex vivo tissue, Evans blue at 50x and 500x dilution, and porphyrin-lipid.

S1.15 Statistical analysis

All data is presented as the mean ± standard deviation, unless otherwise stated. For calculated ratios demonstrating impact of charge, chain length, FUS, and perfusion, error bars represent standard deviations generated through propagation of error associated with the relevant groups. Statistical analyses were performed in R Statistical Software (v4.1.2; R Core Team 2021), GraphPad Prism 8, and Matlab® (MathWorks, Natick, MA). Non-parametric tests were conducted, unless otherwise stated. Comparison of two or more groups was conducted using the Kruskal-Wallis test with post-hoc Dunnet’s test to denote corrected significant differences. Comparison of two groups was assessed with Wilcoxon rank-sum tests and a significance threshold of $\alpha = 0.05$. For multiple comparisons, significance was found following Benjamini-Hochberg correction based on the number of tests performed to reduce the false discovery rate (set at 5 or 10%). Here, p values less than the critical value defined as (i/m)Q were considered significant, where i=p value rank, m=number of tests, and Q=false discovery rate (as indicated in appropriate figures). Statistical difference in tumor shell accumulation following FUS was assessed with one-tailed t-tests, as accumulation was expected to increase in a single direction with FUS.

## S2. Supplementary Tables

**Table S1.** pDef compositions provided in molar%**.** Pyro-lipid = pyropheophorbide-a-lipid, DPPC = dipalmitoylphosphatidylcholine, DSPC = 1,2-distearoyl-sn-glycero-3-phosphocholine, DBPC 1,2-dibehenoyl-sn-glycero-3-phosphocholine, DPPA = 1,2-dipalmitoyl-sn-glycero-3-phosphate (sodium salt), DSPA = 1,2-distearoyl-sn-glycero-3-phosphate (sodium salt), DPPE-mPEG 500 = 1,2-dipalmitoyl-sn-glycero-3-phosphoethanolamine-N-[methoxy(polyethylene glycol)-5000] (ammonium salt), DSPE-mPEG 5000 = 1,2-distearoyl-sn-glycero-3-phosphoethanolamine-N-[methoxy(polyethylene glycol)-5000] (ammonium salt). For reference, the composition of Definity® is also provided.

| pDef | Porphryin-lipid (zwitterionic) | | Host PC lipid (zwitterionic) | | PA anionic group | | PEG lipid (zwitterionic) | |
|---|---|---|---|---|---|---|---|---|
| C16 | C16 pyro-lipid | 30 mol% | DPPC | 52 mol% | DPPA | 10 mol% | DPPE-mPEG 5000 | 8 mol% |
| C16 1% | C16 pyro-lipid | 1 mol% | DPPC | 81 mol% | DPPA | 10 mol% | DPPE-mPEG 5000 | 8 mol% |
| C16 10% | C16 pyro-lipid | 10 mol% | DPPC | 62 mol% | DPPA | 10 mol% | DPPE-mPEG 5000 | 8 mol% |
| C18 | C18 pyro-lipid | 30 mol% | DSPC | 52 mol% | DSPA | 10 mol% | DSPE-mPEG 5000 | 8 mol% |
| C16nPA | C16 pyro-lipid | 30 mol% | DPPC | 62 mol% | - | - | DPPE-mPEG 5000 | 8 mol% |
| C18nPA | C18 pyro-lipid | 30 mol% | DSPC | 62 mol% | - | - | DSPE-mPEG 5000 | 8 mol% |
| C22nPA | C22 pyro-lipid | 30 mol% | DBPC | 62 mol% | - | - | DSPE-mPEG 5000 | 8 mol% |
| Definity® | - | - | DPPC | 82 mol% | DPPA | 10 mol% | DPPE-mPEG 5000 | 8 mol% |

**Table S2.** Physicochemical properties of unlabeled and Cu-chelated pDefs. All measurements represent an average ± standard deviation (n=3-7)

| pDef variant | Yield [x10$^9$ MB▪mL$^{-1}$] | Gas volume [x10$^{10}$ µm$^3$ ▪mL$^{-1}$] | Surface area [x10$^{10}$ µm$^2$ ▪mL$^{-1}$] | Mean size by number distribution [µm] | Mean size by volume distribution [µm] | Mean size by surface area distribution [µm] | $\varepsilon_{702nm}$ [x10$^4$ M$^{-1}$•cm$^{-1}$] | $Q_{702nm}$:$Q_{674nm}$ | Fl quenching efficiency [%] | Zeta potential [mV] |
|---|---|---|---|---|---|---|---|---|---|---|
| In-house Definity | 10 ± 4 | 1.8 ± 0.2 | 5.0 ± 0.9 | 1.07 ± 0.03 | 3.64 ± 0.09 | 2.23 ± 0.06 | N/A | N/A | N/A | -44 ± 3 |
| C16 | 3 ± 2 | 6 ± 3 | 3 ± 4 | 1.7 ± 0.2 | 6.0 ± 0.8 | 4.0 ± 0.5 | 5.6 ± 0.3 | 2.5 ± 0.1 | 97.5 ± 0.8 | -39 ± 3 |
| Cu-C16 | 3 ± 2 | 4 ± 2 | 5 ± 3 | 1.8 ± 0.2 | 6.6 ± 0.8 | 4.7 ± 0.4 | 5.0 ± 0.5 | 2.3 ± 0.2 | 98 ± 2 | -42 ± 2 |
| C16nPA | 8.4 ± 0.4 | 2.3 ± 0.8 | 5 ± 1 | 1.1 ± 0.2 | 4.6 ± 0.1 | 2.8 ± 0.3 | 4.7 ± 0.7 | 2.1 ± 0.5 | 98.2 ± 0.3 | -13.2 ± 0.7 |
| Cu-C16nPA | 6.2 ± 0.5 | 2.9 ± 0.9 | 5 ± 1 | 1.3 ± 0.2 | 5.1 ± 0.3 | 3.4 ± 0.1 | 4.3 ± 0.5 | 1.8 ± 0.6 | 98.7 ± 0.2 | -12.9 ± 0.3 |
| C18 | 8 ± 1 | 3.9 ± 0.9 | 8 ± 1 | 1.5 ± 0.1 | 4.0 ± 0.5 | 3.2 ± 0.5 | 5.3 ± 0.3 | 2.5 ± 0.3 | 97 ± 1 | -44 ± 2 |
| Cu-C18 | 7 ± 2 | 4 ± 1 | 7 ± 1 | 1.5 ± 0.2 | 4.9 ± 0.9 | 3.4 ± 0.6 | 4.9 ± 0.8 | 2.2 ± 0.5 | 98.5 ± 0.6 | -42 ± 2 |
| C18nPA | 8 ± 1 | 3 ± 1 | 7 ± 1 | 1.5 ± 0.1 | 4.0 ± 0.8 | 2.9 ± 0.6 | 4 ± 1 | 1.8 ± 0.7 | 98.6 ± 0.5 | -11 ± 1 |
| Cu-C18nPA | 7.8 ± 0.2 | 3.9 ± 0.2 | 7.3 ± 0.4 | 1.42 ± 0.04 | 4.8 ± 0.1 | 3.22 ± 0.09 | 4 ± 1 | 1.8 ± 0.7 | 98.7 ± 0.3 | -14 ± 2 |

*In-house Definity® is C16 pDef with 0 mol% porphyrin

**Table S3.** Microbubbles kinetic parameters from acoustic renal imaging (0.2 as sig. Fig. b/c frame rate is ~5/sec). Values are average +/- SD with the exception of *Dissolution half-life, showing modeled value with 95% confidence interval

| pDef | Mean transit time [s] | Total transit time [s] | Rising time [s] | Falling time [s] | Dissolution half-life [s]* | Linear rate of wash-out [s$^{-1}$] | Gamma-Variate Kinetic [1/ß] |
|---|---|---|---|---|---|---|---|
| C16 | 12 ± 2 | 40 ± 14 | 4.4 ± 0.6 | 36 ± 14 | 7.47 (7.38 to 7.57) | -5 ± 1 | 1.4 ± 0.6 |
| C18 | 6 ± 3 | 24 ± 11 | 2.8 ± 0.8 | 21 ± 10 | 3.93 (3.85 to 4.01) | -12 ± 3 | 0.4 ± 0.2 |
| C16nPA | 12 ± 4 | 38 ± 13 | 4 ± 2 | 33 ± 13 | 6.84 (6.73 to 6.96) | -6 ± 2 | 1.0 ± 0.8 |
| C18nPA | 15 ± 4 | 114 ± 31 | 4 ± 2 | 110 ± 31 | 13.2 (13.1 to 13.3) | -3 ± 2 | 4 ± 3 |
| C22nPA | 31 ± 6 | 63 ± 14 | 4 ± 2 | 59 ± 16 | 16.2 (16.1 to 16.4) | -1.7 ± 0.4 | 13 ± 7 |
| In-house Definity® | 99 ± 40 | 213 ± 69 | 14 ± 12 | 199 ± 63 | 67.1 (66.4 to 67.9) | -0.6 ± 0.2 | 41 ± 28 |

**Table S4.** Microbubble shell blood clearance kinetic parameters. 95% Confidence intervals are supplied in the brackets.

| pDef | Porphyrin concentration [µM] | Rate constant $K_{slow}$ [h-1] | Rate constant $K_{fast}$ [h-1] | $t_{1/2}$ (slow) [h] | $t_{1/2}$ (fast) [h] | % Fast phase |
|---|---|---|---|---|---|---|
| C16 | 200 | 0.062<br>(0.040 to 0.097) | 2.20<br>(1.83 to 2.72) | 11.2<br>(7.13 to 17.2) | 0.31<br>(0.26 to 0.38) | 74.8<br>(70.2 to 78.7) |
| C16 10% | 66.7 | 0.073<br>(0.044 to 0.12) | 2.73<br>(2.00 to 4.00) | 9.56<br>(5.73 to 15.9) | 0.25<br>(0.17 to 0.35) | 68.7<br>(62.5 to 74.0) |
| C16 1% | 6.67 | 0.063<br>(0.041 to 0.096) | 1.40<br>(0.98 to 2.08) | 11.0<br>(7.20 to 16.73) | 0.49<br>(0.33 to 0.71) | 57.9<br>(49.1 to 65.5) |
| C18 | 200 | 0.15<br>(0.12 to 0.18) | 2.97<br>(2.03 to 4.72) | 4.56<br>(3.84 to 5.64) | 0.23<br>(0.15 to 0.34) | 43.9<br>(39.2 to 49.2) |
| C16nPA | 200 | 0.079<br>(0.053 to 0.12) | 3.33<br>(2.66 to 4.44) | 8.75<br>(5.81 to 13.13) | 0.21<br>(0.16 to 0.26) | 71.3<br>(67.4 to 74.7) |
| C18nPA | 200 | 0.082<br>(0.051 to 0.13) | 2.13<br>(1.37 to 3.77) | 8.43<br>(5.33 to 13.63) | 0.32<br>(0.18 to 0.51) | 56.4<br>(47.5 to 64.3) |
| C22nPA | 200 | 0.089<br>(0.072 to 0.11) | 2.19<br>(0.97 to 9.50) | 7.80<br>(6.46 to 9.66) | 0.31<br>(0.073 to 0.72) | 24.4<br>(17.0 to 31.8) |

**Table S5.** AUCs for pDef contrast imaging and shell circulation profiles

| pDef | AUC contrast [normalized contrast•s] | AUC shell circulation [normalized mCi•h] | AUC shell circulation/ AUC core contrast circulation |
|---|---|---|---|
| C16 | 10.7 (10.4 to 11.0) | 5.19 (4.13 to 6.25) | 1746 |
| C18 | 7.31 (6.87 to 7.75) | 5.93 (4.73 to 7.13) | 2920 |
| C16nPA | 10.6 (10.1 to 11.1) | 5.64 (5.04 to 6.23) | 1915 |
| C18nPA | 20.9 (20.6 to 21.2) | 7.05 (5.73 to 8.37) | 1214 |
| C22nPA | 23.6 (23.5 to 23.8) | 9.80 (8.43 to 11.2) | 1495 |

## S3. Supplementary Figures

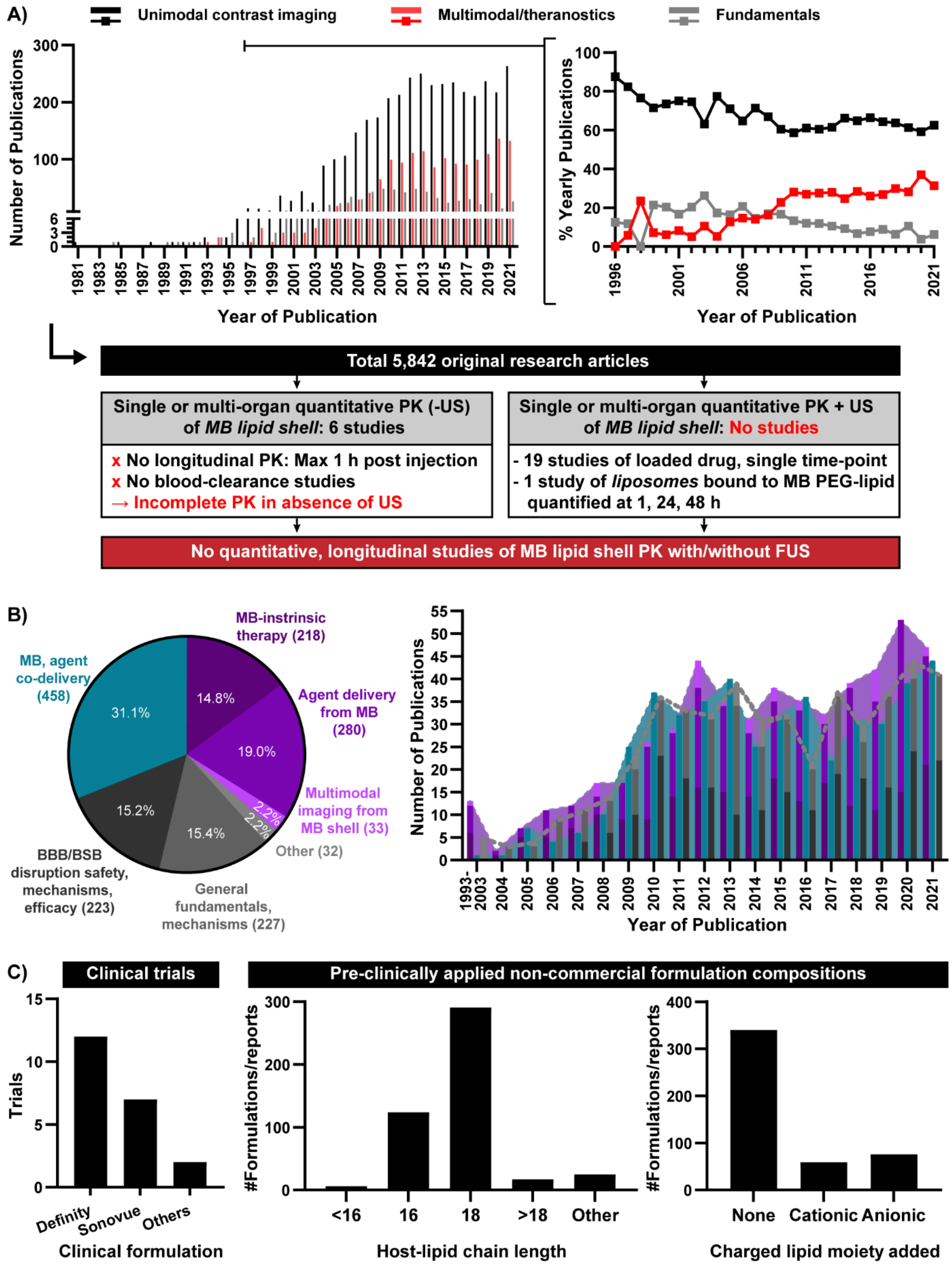


**Figure S1**: Literature review of microbubble-mediated focused ultrasound (MB-FUS) platforms for drug delivery. **A)** Number of publications since 1981 on the use of microbubbles for unimodal contrast imaging (black), multimodal/theranostic applications including drug delivery (red), and fundamental physics studies (grey), highlighting the recent increase in theranostic applications in recent years. **B)** Categorized theranostic uses of MB-FUS over time, highlighting that shell-loading (purple) and co-injection (teal) represent the two primary forms of use. **C)** Clinical trials using commercial microbubbles most commonly use Definity® and Sonovue®. Overviewing pre-clinical formulations shows that most bubbles are constructed using either C16 or C18 chain length lipids, and that uncharged followed by anionic lipid constituents are most widely used.

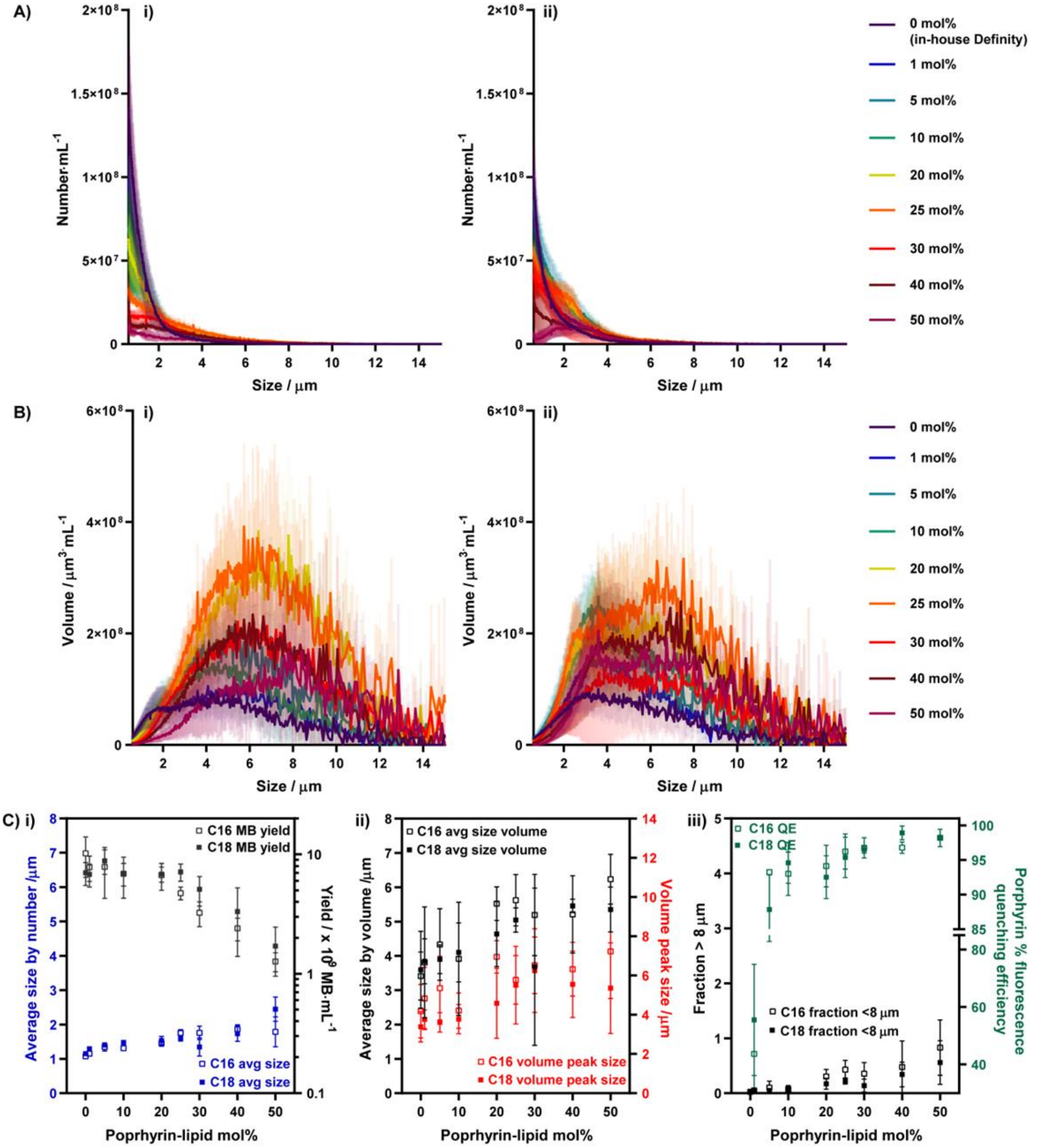


**Figure S2**: Optimization of porphyrin-lipid loading into C16 and C18 pDef formulations from 0–50 mol%. Note: C16 0 mol% formulation represents in-house fabricated Definity(R). **A)** Number-weighted and **B)** volume-weighted size distributions from 0.6–15 µm for (i) C16 and (ii) C18 pDefs. **C)** (i) Mean microbubble size (evaluated on number-weighted size distribution) increases with increasing fractional porphyrin-lipid, while overall microbubble yield decreases. (ii) Mean microbubble size and peak position (evaluated on volume-weighted size distribution) increases with increasing fractional porphyrin-lipid. (iii) The fraction of microbubbles over 8 µm remains below 1% up to 50 mol% porphyrin-lipid inclusion. Porphyrin fluorescence quenching efficiency exceeds 95% for all porphyrin-lipid molar inclusion fractions above 10%. All work henceforth utilizes a 30 mol% of porphyrin-lipid to balance porphyrin maximization while conserving average microbubble size, concentration and sub 8 µm fraction.

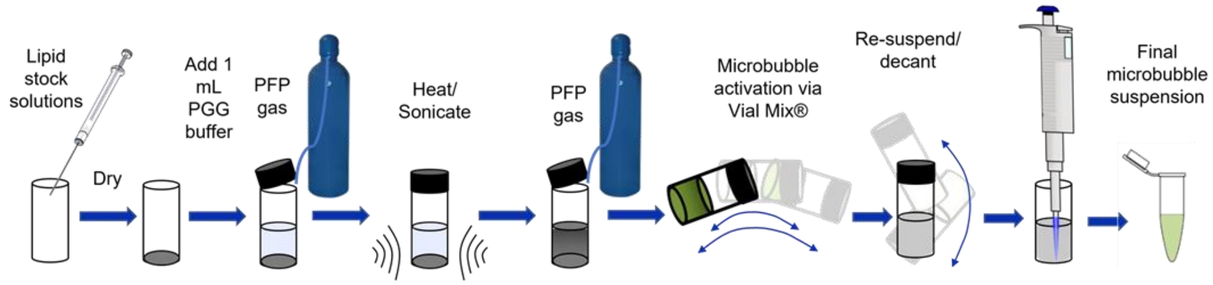


**Figure S3**: Lipid hydration protocol used to generate lipid microbubbles, including all pDef formulations.

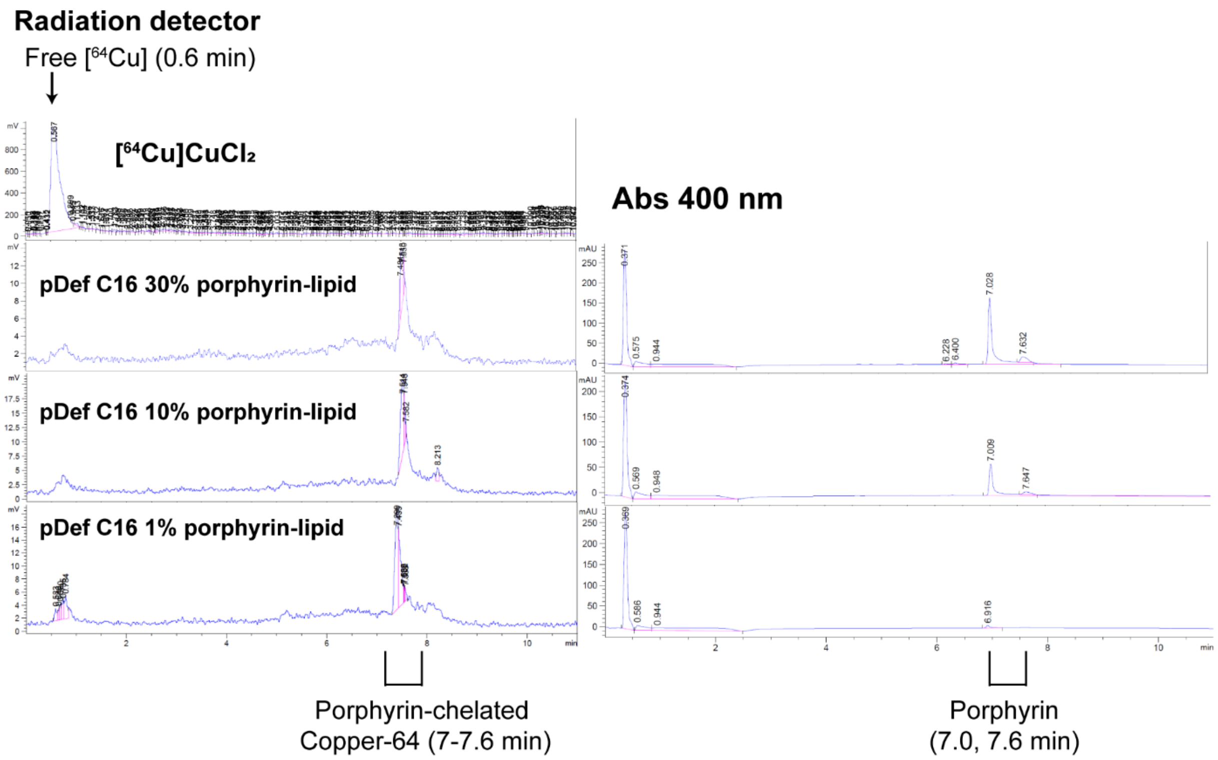


**Figure S4**: Radio-UPLC characterization of lipid suspensions with 1, 10, and 30 mol% porphyrin-lipid after [$^{64}$Cu]$Cu^{2+}$ chelation and prior to pDef activation. The broad peak within the radiation spectra centred at 7.3 min represents $^{64}$Cu-chelated porphyrin-lipid. Comparison of this peak to that of free Copper-64 (0.6 min) demonstrates that the chelation reaction yielded predominantly porphyrin-lipid chelated [$^{64}$Cu]$Cu^{2+}$. As can be seen by absorbance spectra, signal, possibly associated with porphyrin or contaminants was also observed at retention times associated with free Copper-64, making it difficult to use radio-UPLC to quantify radiochemical purity or chelation efficiency. As such, validated alternatives (iTLC and gamma counting post centrifugal separation of free and chelated copper) were used for such quantifications.

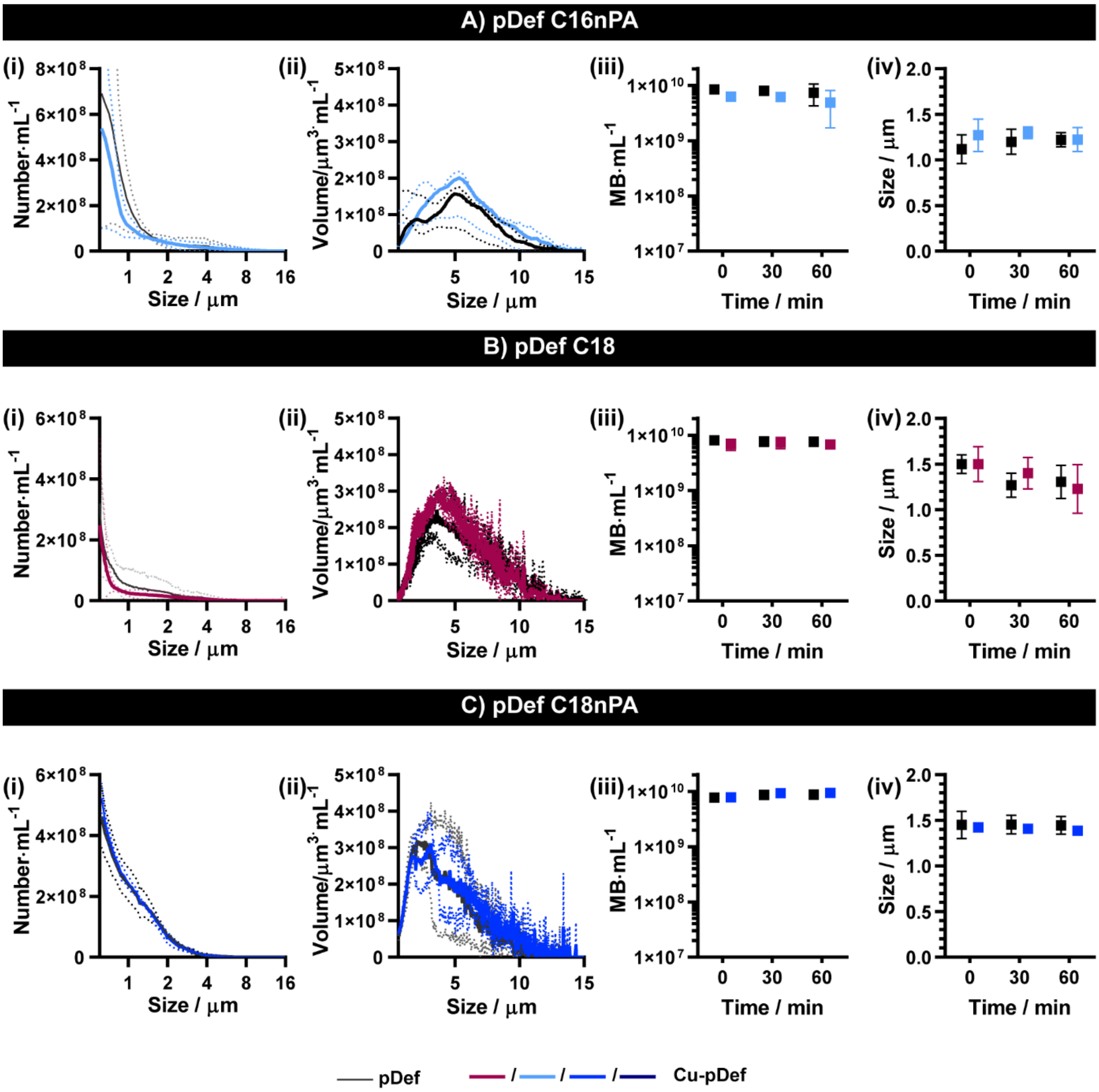


**Figure S5**: Effect of copper-labelling on microbubble sizing and stability for **A)** C16nPA, **B)** C18, and **C)** C18nPA pDefs, including (i) number-weighed size distributions and (ii) volume-weighted size distributions. Colored lines represent copper-labelled microbubbles, while black lines represent unlabeled microbubbles, showing no apparent changes. (iii) Overall microbubble yield (solid black: copper-labelled, empty black: unlabeled)) and number-weighted mean microbubble size (solid teal: copper-labelled, empty teal: unlabeled) are also unchanged following the post-insertion, pre-activation chelation procedure. Plots are presented as averages of n=3-5 replicates ± standard deviation presented as dotted lines in (i) and error bars in (iii) and (iv). The number distribution for C18nPA pDef and Cu-pDef overlap completely.

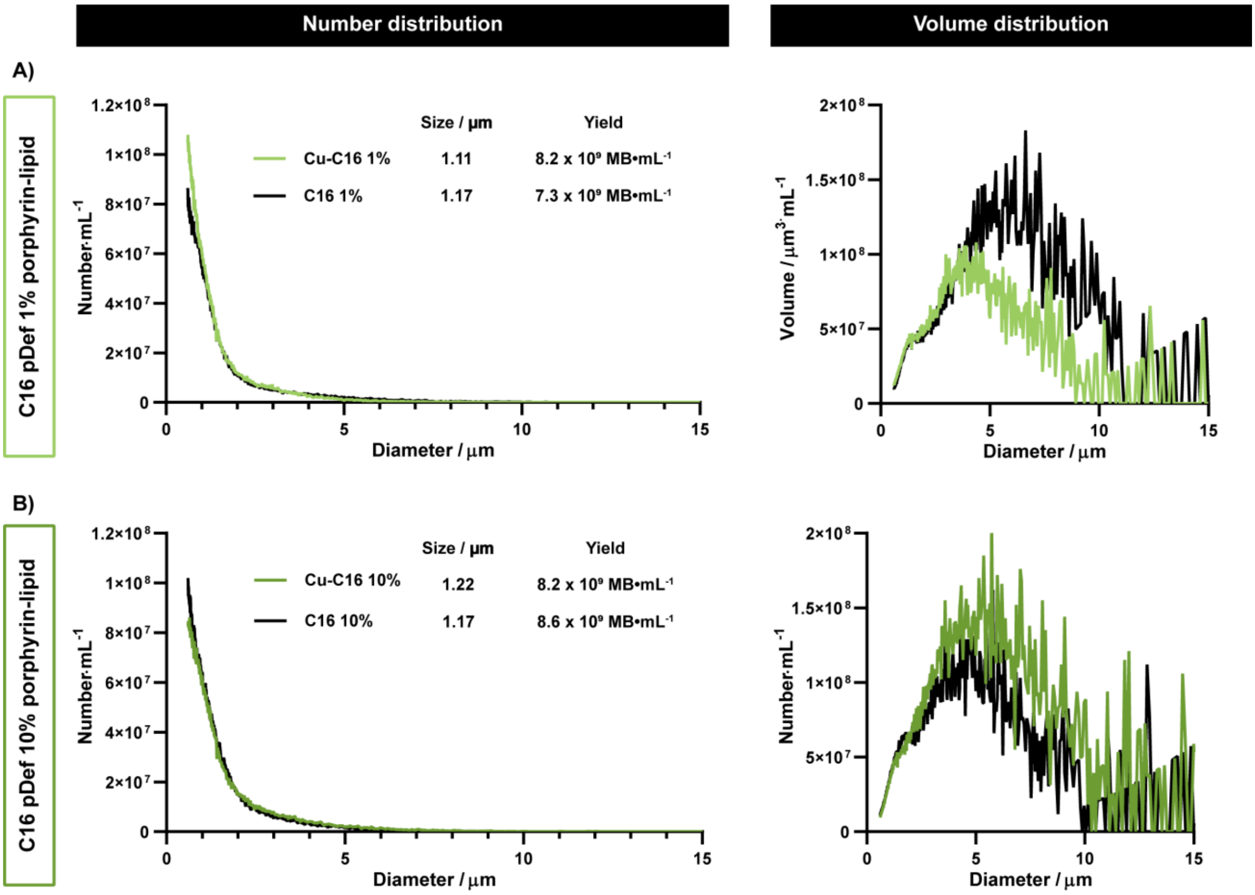


**Figure S6**: Cu-labelled (green) and unlabeled (black) number-weighted and volume-weighted microbubble size distributions at low porphyrin lipid inclusion percentages, including **A)** 1 mol% and **B)** 10 mol%. Overall microbubble yield and mean size from the number-weighted distribution are provided as inset values.

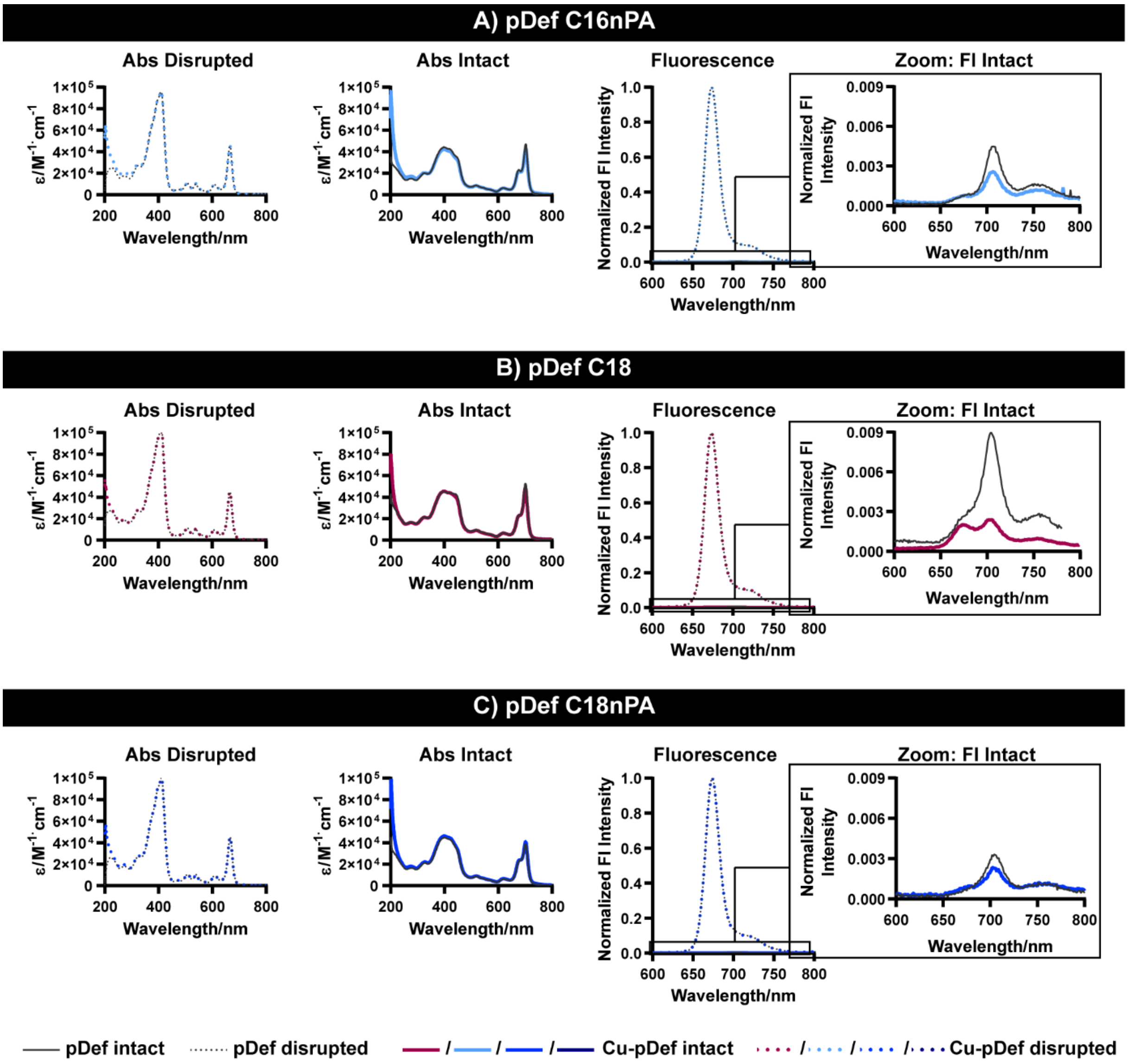


**Figure S7**: Effect of copper-labelling on absorbance and fluorescence of **A)** C16nPA, **B)** C18, and **C)** C18nPA pDefs. Colored lines represent copper-labelled microbubbles, black lines represent unlabeled microbubbles, solid lines represent intact microbubbles, and dashed lines represent surfactant or methanol-treated disrupted microbubbles. All absorbance spectra illustrate that intact structures exhibit a red-shifted Q band indicative of ordered porphyrin aggregation. All fluorescent spectra show a high degree of quenching for intact structures relative to disrupted structures, with copper-chelated structures showing an even stronger quenching effect than unlabeled microbubbles. Beyond this, Cu labeling did not change absorbance and fluorescence profiles of pDefs. All plots represent averages of n=3-8 replicates. The labeled and unlabeled pDef absorbance profiles (intact and disrupted) as well as disrupted particle fluorescence spectra completely overlap with one another.

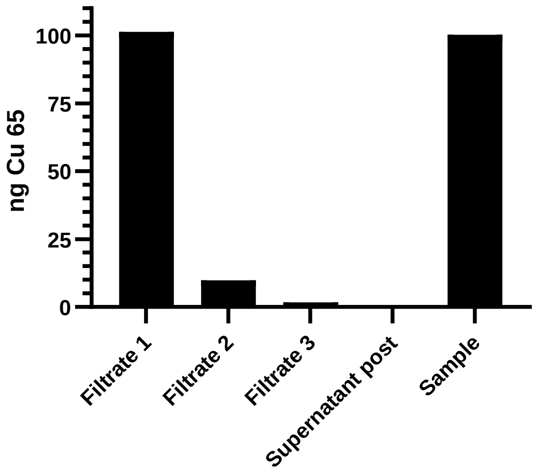


**Figure S8**: Validation of centrifugation method to evaluate Copper-64 chelation efficiency. Free copper was added to the filter unit at quantities and concentrations representative of that added to microbubble mixtures. Supernatants and filtrates were collected from three successive centrifugation cycles, demonstrating that this process was able to remove all free copper from the unit supernatant, which was recovered completely in the filtrates.

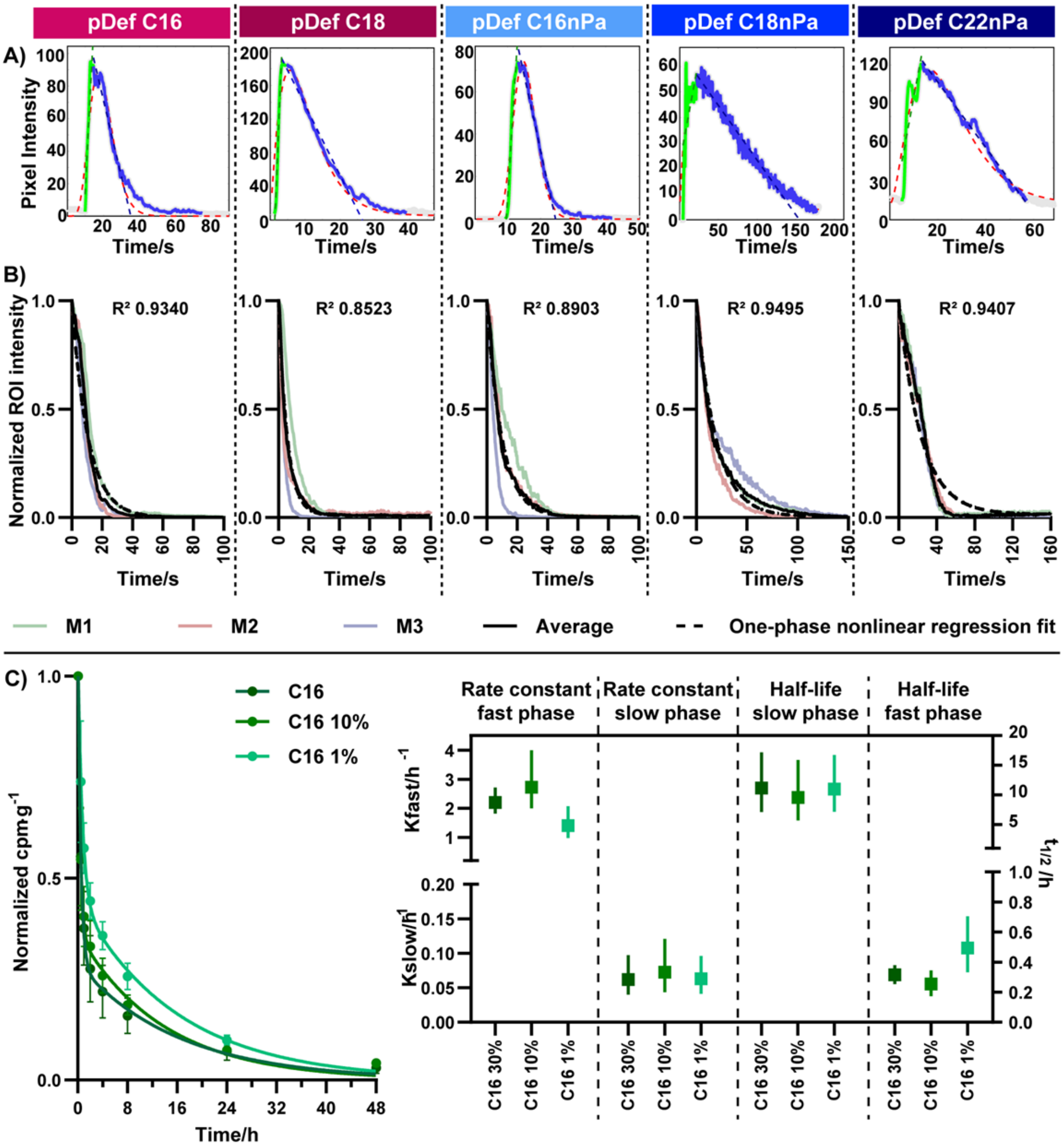


**Figure S9**: Microbubble wash-in/wash-out curve modeling. **A)** Sample time series contrast data for each microbubble type (gray) showcasing total curve modeling using a gamma-variate function (dashed red), as well as the portions of the data selected for modeling wash-in (solid green) and wash-out (solid blue) and their associated linear fits (dashed dark green and dashed dark blue, respectively). Coefficients are determination are inset in each graph for each of the three models, and key fitting parameters are listed in Table S3. **B)** Peak-to-wash-out portion of all contrast curves (n=3 mice) and average wash-out (black) overlaid with one-phase exponential (t-test vs two-phase exponential decay, $p < 0.05$) and inset with coefficient of determination. **C)** Quantitative shell fragment clearance data from C16 pDefs with 30 mol%, 10 mol%, and 1 mol% porphyrin-lipid, measures via γ-counting of blood samples over 48 hours. All curves were best fit by two-phase exponential decay (t-test vs one-phase exponential decay, $p < 0.05$). Rate constants and the inversely related half-lives for these slow and fast rates of clearance are provided compared, showing no major changes at lower porphyrin-lipid inclusion fractions.

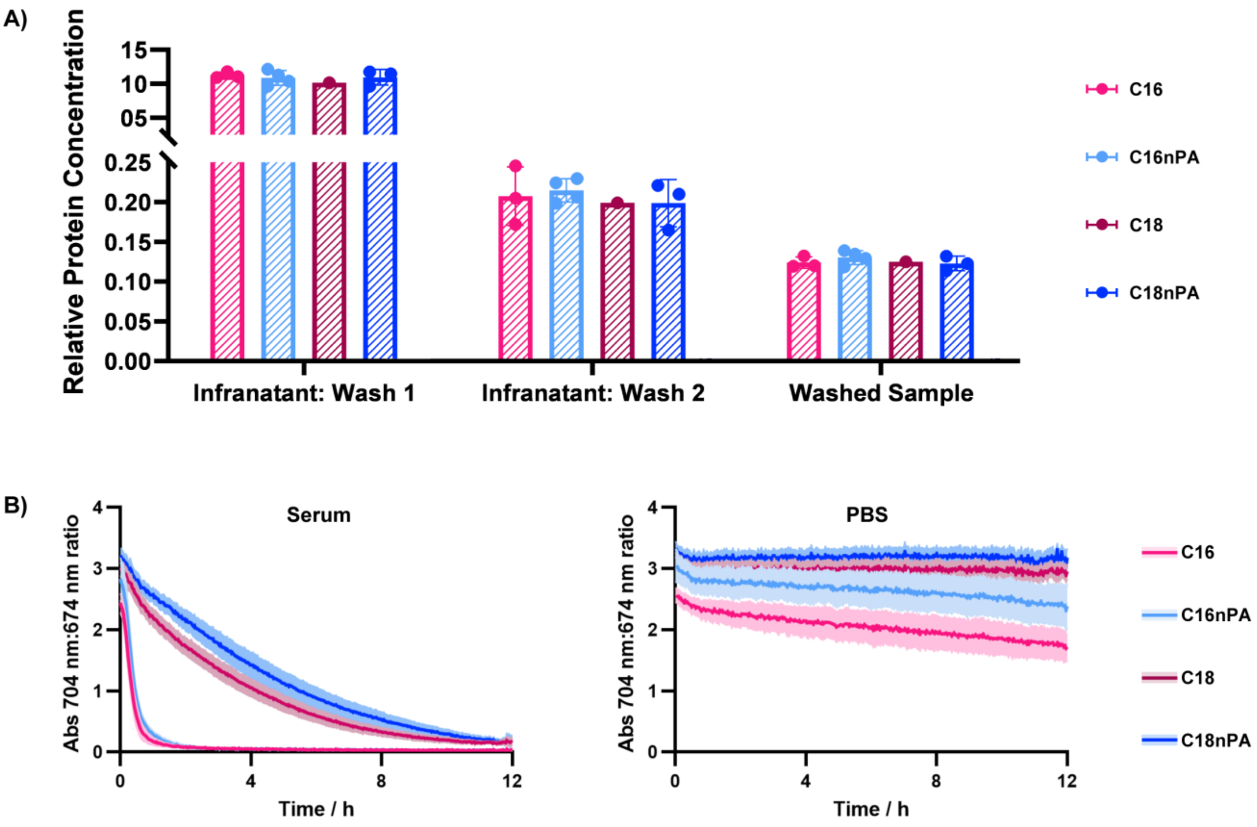


**Figure S10**: Microbubble structural changes and destabilization induced by interaction with serum proteins. **A)** Non-specific protein binding (relative protein concentration in final washed sample) as evaluated using a BCA protein assay shows no differential binding profile between pDefs following brief serum incubation (5 minutes). **B)** Ratio of pDef absorbance at 704 nm (red-shifted Q-band indicative of ordered porphyrin aggregation) and 674 nm (unordered porphyrin Q-band) following 12-hour incubation in 50:50 FBS:PBS (Serum) or PBS. This ratio rapidly decreases for C16 and C16nPA pDefs, indicative of reduced ordered aggregation in serum relative to C18 and C18nPA, respectively.

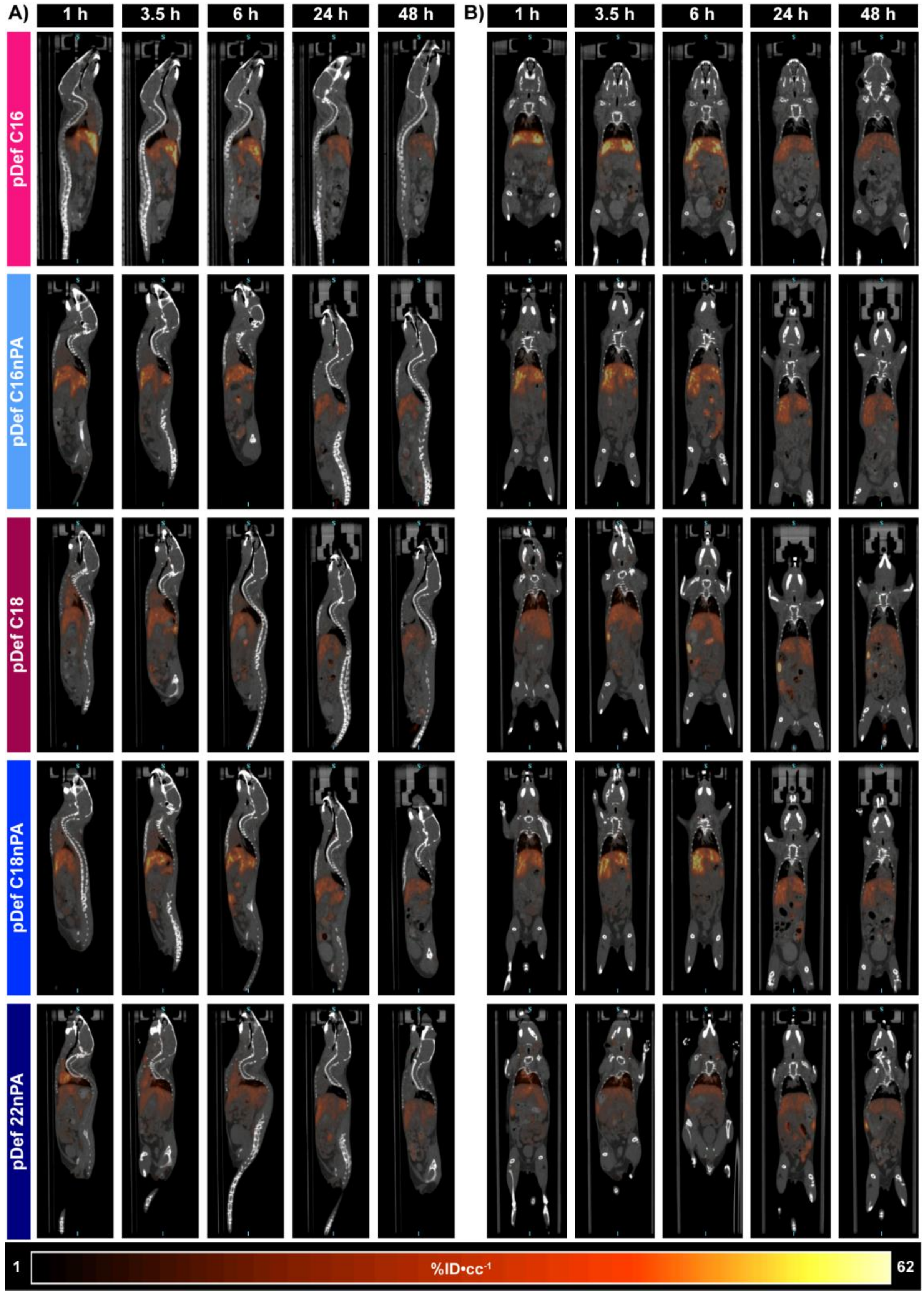


**Figure S11**: **A)** Sagittal and **B)** coronal sample PET/CT images for pDefs at 5 timepoints over 48 hours in healthy, BALB/c mice. Dynamic range of PET is 1–62 %ID·cc$^{-1}$. n=5–8 for each formulation in healthy animals.

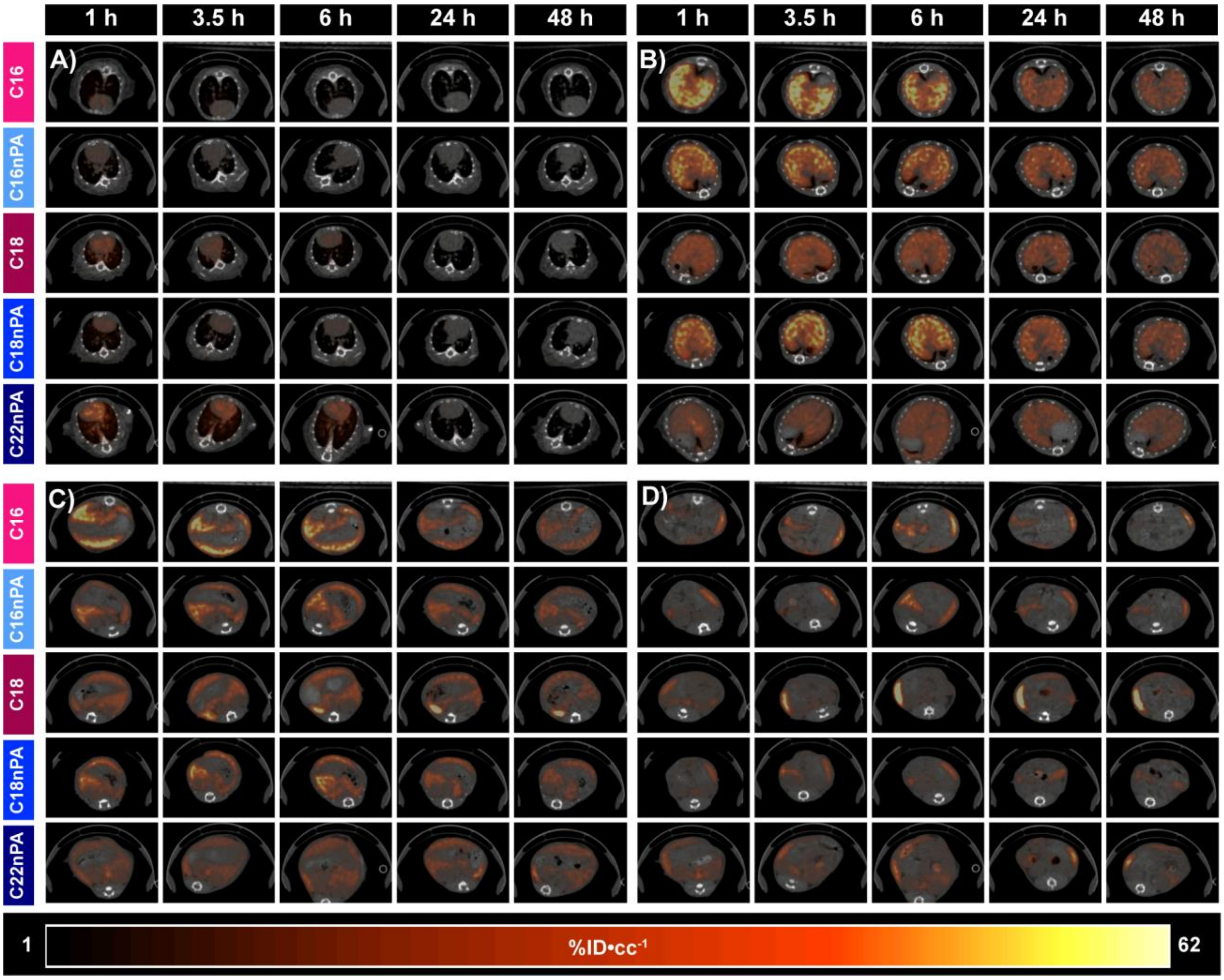


**Figure S12**: Axial sample PET/CT images for pDefs at 5 timepoints over 48 hours in healthy, BALB/c mice. **A)** Mid thoracic view bisecting heart and lungs, **B)** upper abdominal view capturing dome of liver and stomach, **C)** mid-upper abdominal view capturing descending hepatic lobes and bisecting stomach/pancreas, and **D)** mid abdominal view capturing spleen and intestines. Dynamic range of PET is 1–62 %ID·cc$^{-1}$. N=5-8 for all formulations.

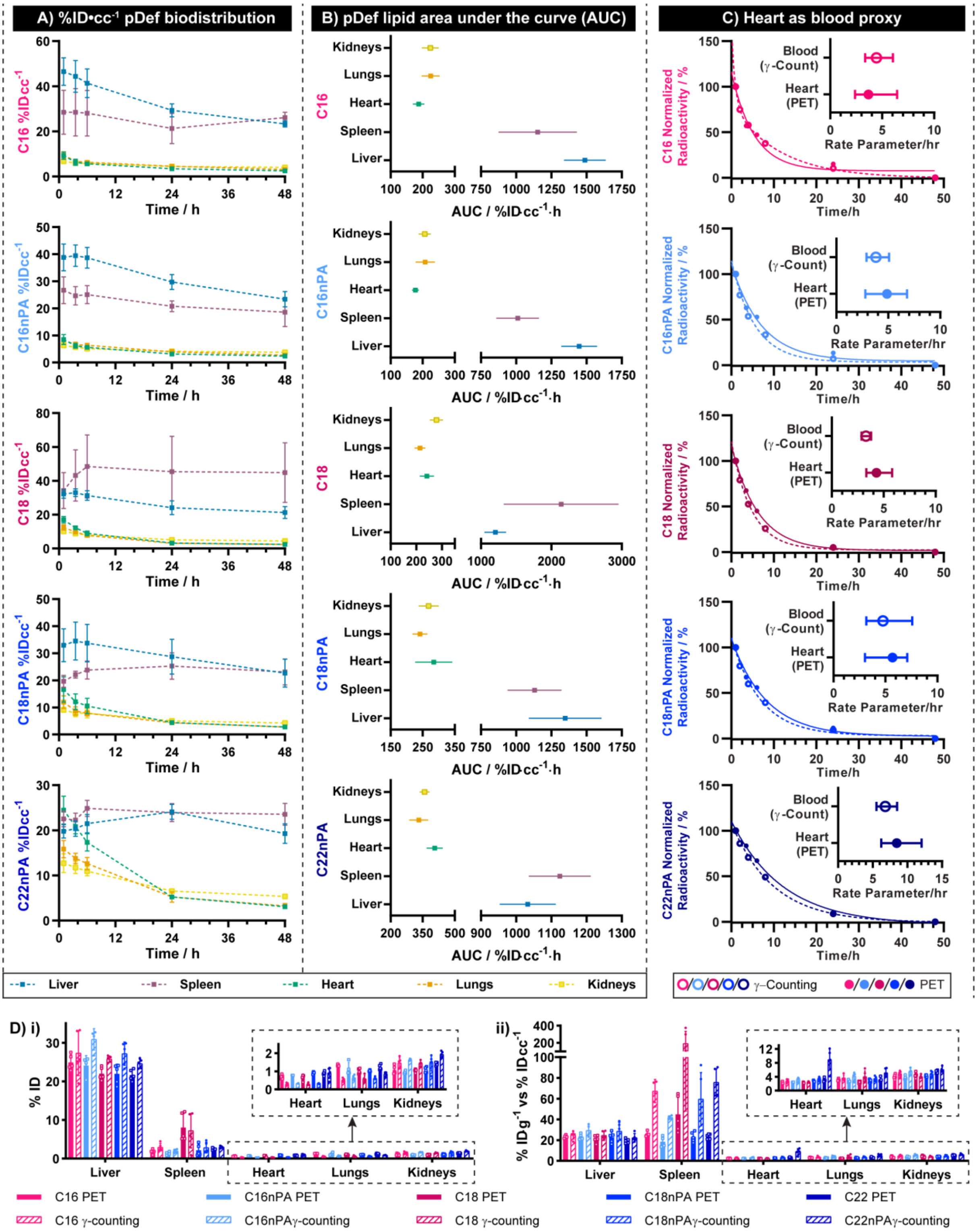


**Figure S13**: Healthy BALB/c pDef pharmacokinetics. **A)** Longitudinal quantitative biodistribution from PET imaging of weight-normalized fractional injected dose (%ID·cc$^{-1}$) and **B)** PET-based area-under-curve (AUC) plots (%ID·cc$^{-1}$·h), comparing relative organ uptake over time and total dose exposure for each pDef formulation, respectively. **C)** Comparison of time-matched blood clearance data obtained from γ-counting to PET-quantified heart signal, illustrating that both predict identifical rate parameters / clearance half-lives, which supports the use of PET signal quantifieid within the heart over time as a proxy for microbubble shell fragment signal within circulation. **D)** Comparison of (i) %ID and (ii) %ID·g$^{-1}$ vs %ID·cc$^{-1}$ obtained from γ-counting and PET image quantification, respectively, showing broad agreement across pDef formulations. Weight-normalized pDef quantitation in the spleen via PET imaging underestimated values relative to γ-counting, likely due to differences between organ volumes and weights. However, PET-based %ID·cc$^{-1}$ quantification retained all observed accumulation trends between different pDef formulations. N=5-8 for all formulations.

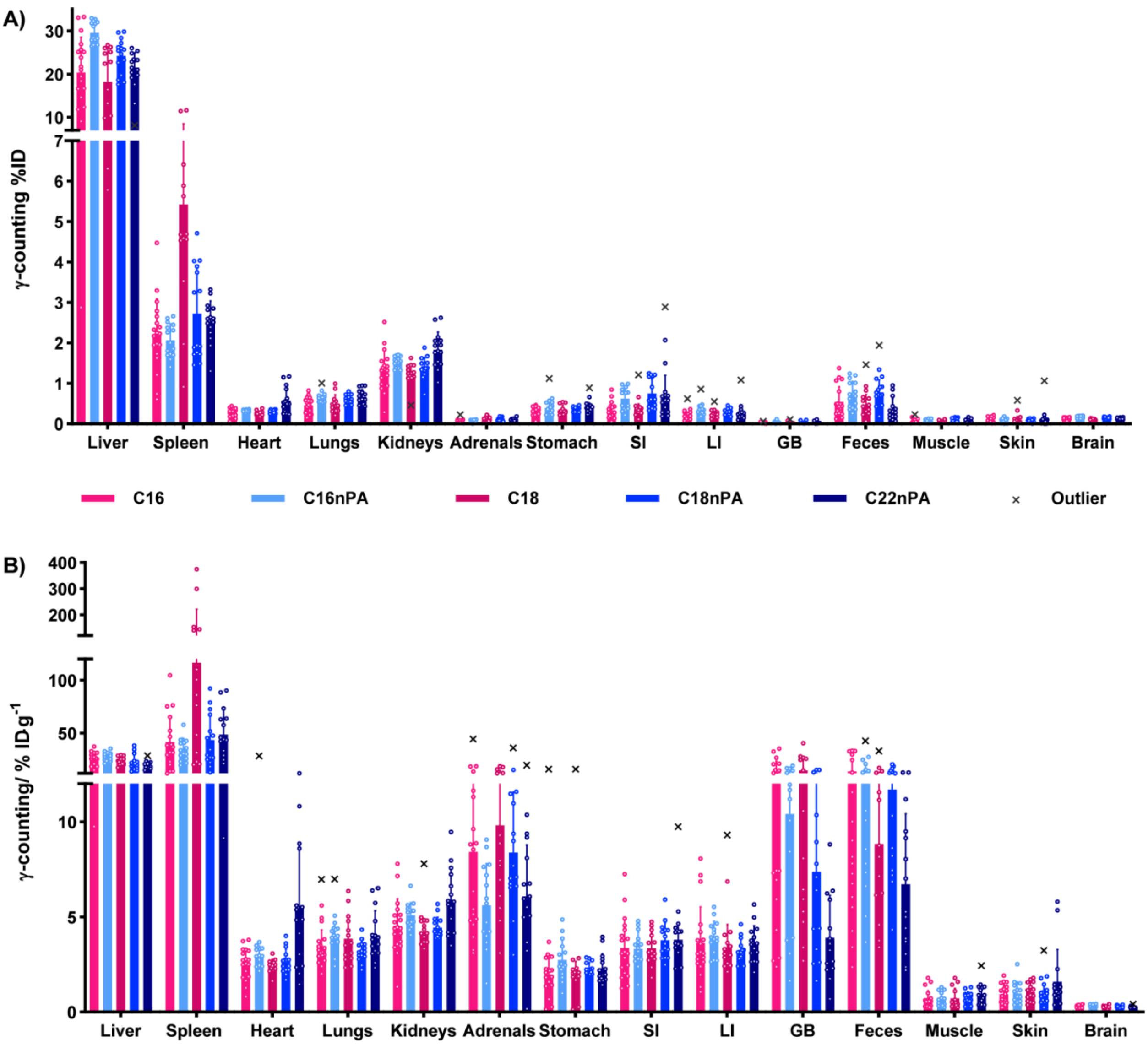


**Figure S14**: Complete healthy BALB/c pDef shell fragment biodistribution assessed by ex vivo tissue γ-counting 48 hours post-injection. Measurements represented as **A)** %ID and **B)** %ID·g$^{-1}$ are shown. Outliers identified by the Grubb's Test are denoted as 'x's within their respective datasets. SI: small intestine, LI: large intestine, GB: intact gall bladder. N=5-8 for all formulations.

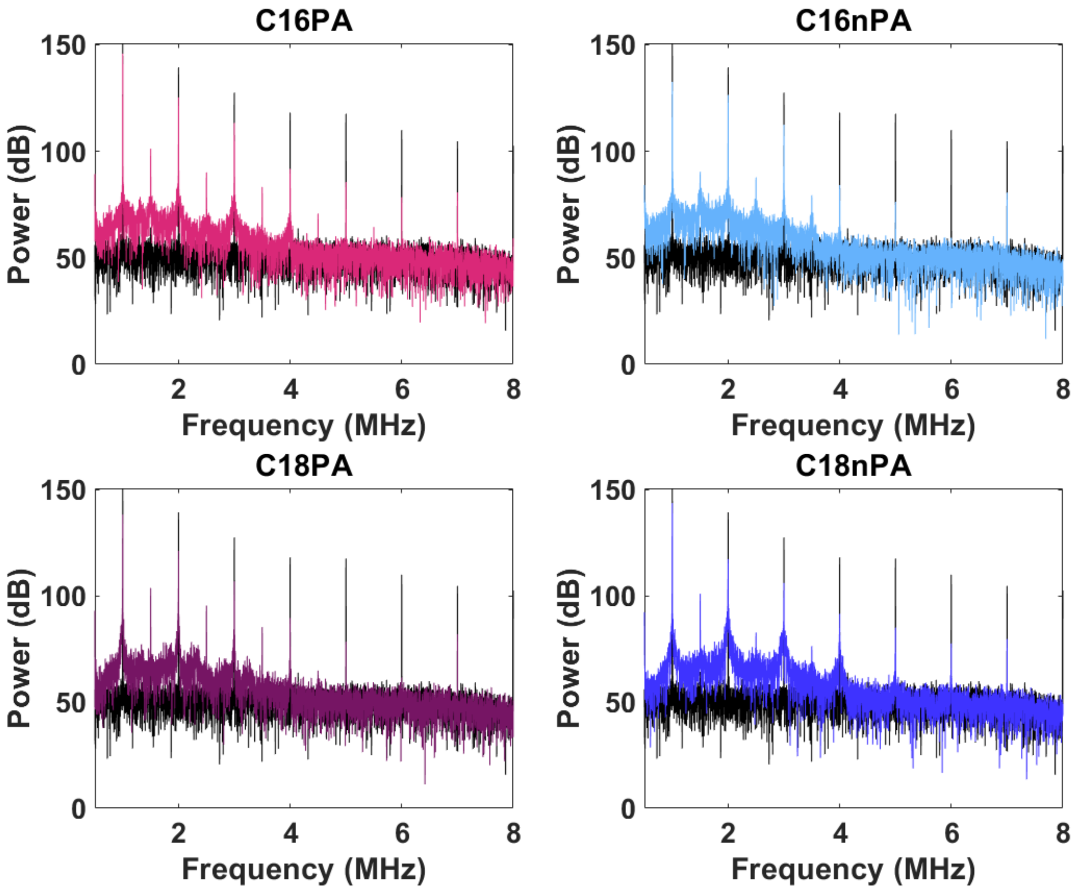


**Figure S15**: Representative power spectra processed from radiofrequency data collected using passive acoustic detection (PAD) during in vivo focused ultrasound sonication of orthotopically-situated 4T1 tumours on BALB/c mice. Baseline measurements (black) were collected for each mouse before microbubble injection, and the acoustic response of microbubbles (colored) was captured within 20 seconds following injection, during which time prior imaging experiments confirm they all retain their capacity to provide acoustic contrast. The emergence of subharmonic peaks and elevated broadband emissions following microbubble injection indicate the presence of stable and inertial cavitation, respectively.

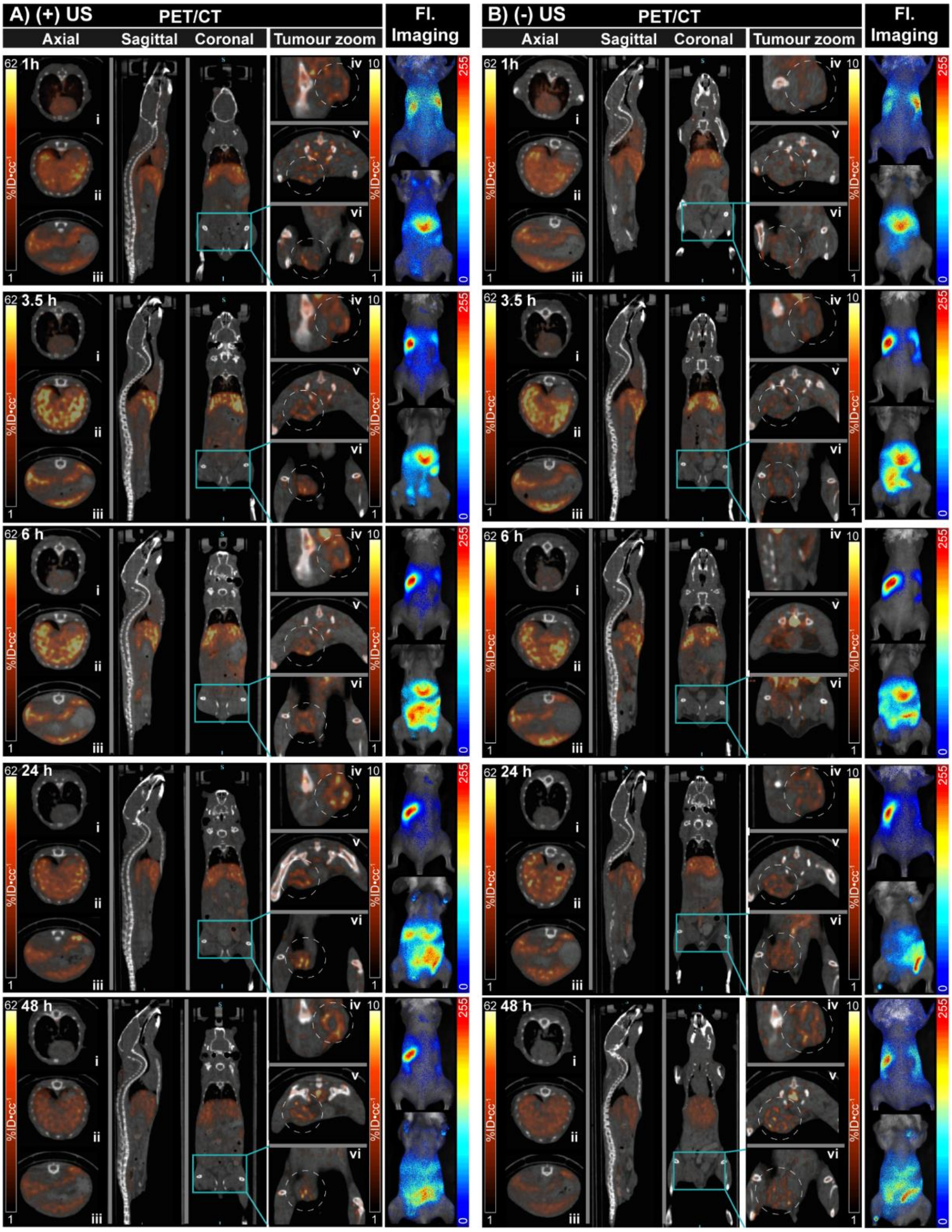


**Figure S16**: C16 pDef representative PET/CT and in vivo hyperspectral fluorescence imaging of 4T1 orthotopic tumor-bearing mice in **A)** FUS-treated and **B)** FUS-untreated cohorts. The different perspectives in i-vi highlight different perspectives or organs, and they are consistent between Figures S16-S19. The views from left to right in each section are as follows: i) Axial – heart; ii) Axial – dome of liver; iii) Axial – inferior portions of liver lobes (left, bottom) and spleen (upper right); Sagittal – liver, stomach, spleen (descending below thorax); Coronal – liver (top abdomen), spleen (right abdomen), tumor at mammary fat pad on lower abdomen; iv) Sagittal – tumor; v) Axial – tumor; vi) Coronal – tumor. PET dynamic range of 1–62 %ID/g for i)-iii) and full body views (left scale bar); PET dynamic range of 1–10 %ID/g for tumor views (right scale bar). Hyperspectral fluorescence imaging dynamic range determined by maximum and minimum intensity values across all data and applied consistently. N=5-8 for all formulations and treatment arms.

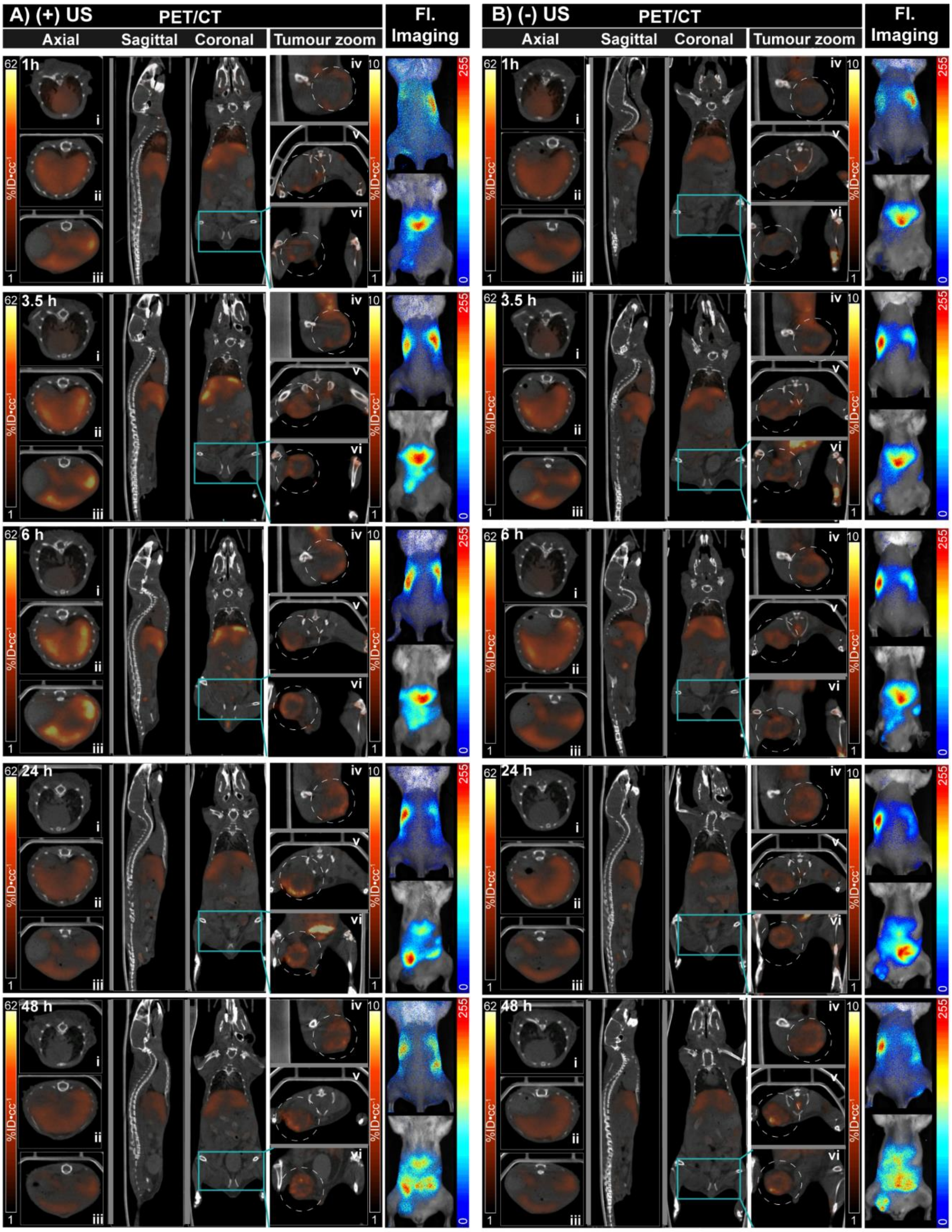


**Figure S17**: C16nPA pDef representative PET/CT and in vivo hyperspectral fluorescence imaging in **A)** FUS-treated and **B)** FUS-untreated animals bearing 4T1 orthotopic tumors. Description of perspectives and dynamic ranges provided in caption for Figure S16. N=5-8 for all formulations and treatment arms.

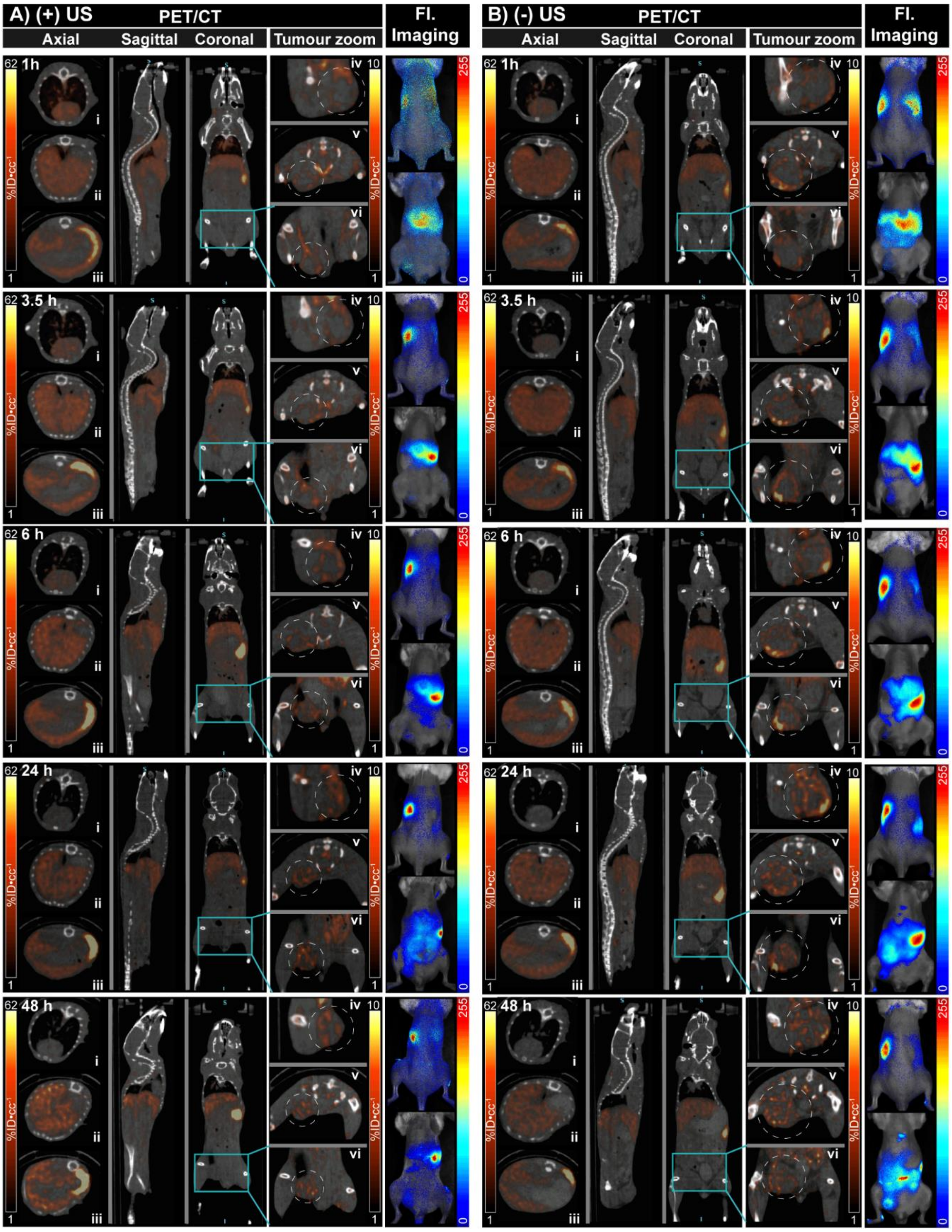


**Figure S18**: C18 pDef representative PET/CT and in vivo hyperspectral fluorescence imaging in **A)** FUS-treated and **B)** FUS-untreated animals bearing 4T1 orthotopic tumors. Description of perspectives and dynamic ranges provided in caption for Figure S16. N=5-8 for all formulations and treatment arms.

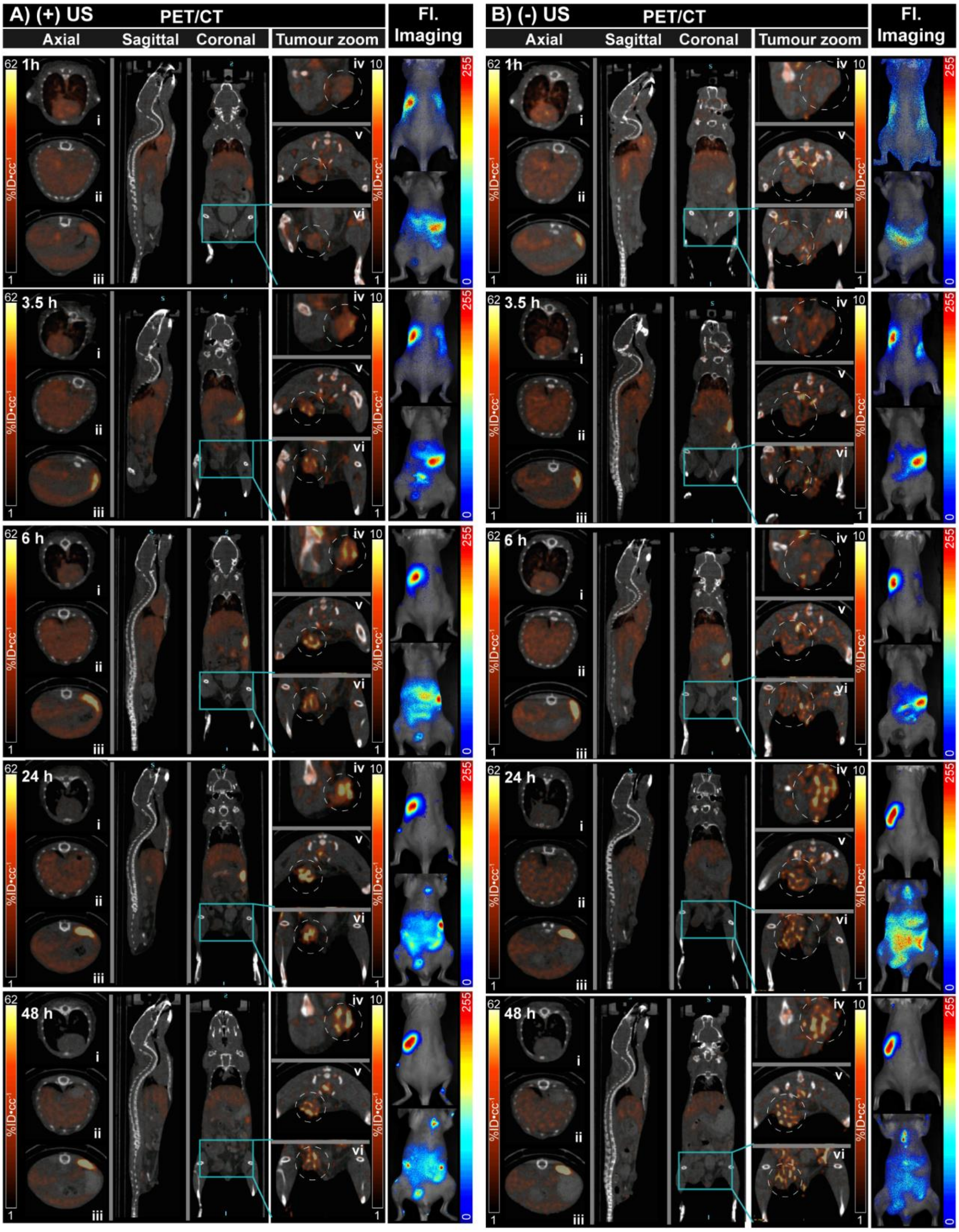


**Figure S19**: C18nPA pDef representative PET/CT and in vivo hyperspectral fluorescence imaging in **A)** FUS-treated and **B)** FUS-untreated animals bearing 4T1 orthotopic tumors. Description of perspectives and dynamic ranges provided in caption for Figure S16. N=5-8 for all formulations and treatment arms.

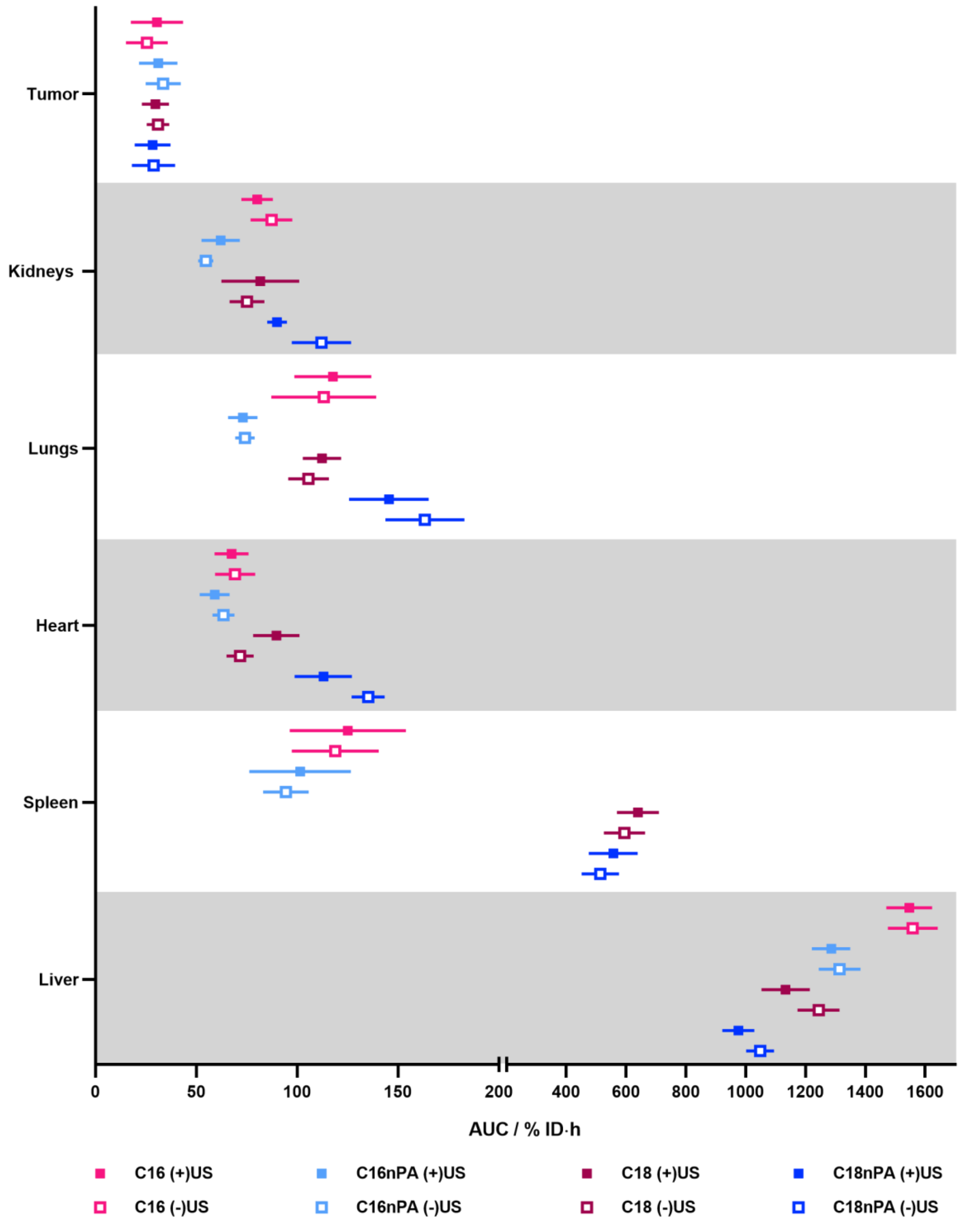


**Figure S20**: Cumulative dose exposure for each organ in the form of area-under-curve (AUC) of pDef biodistribution for PET-quantified %ID time series data in 4T1 orthotopic tumor-bearing mice. Solid markers indicate FUS-treated animals, while empty markers indicate FUS-untreated animals. All calculated AUC values are presented as mean ± 95% CIs. N=5-6 for all formulations and treatment arms.

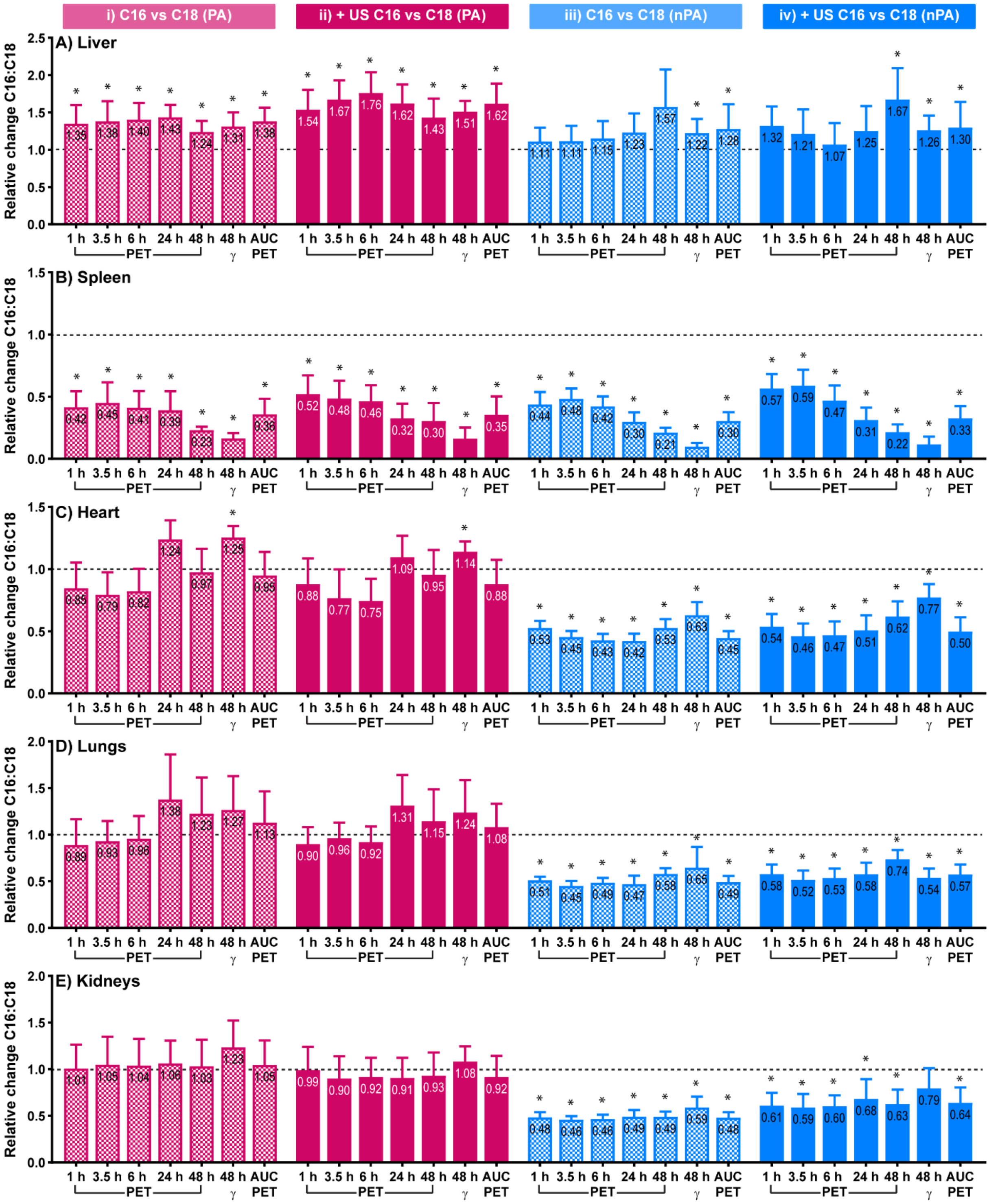


**Figure S21**: Illustrating the impact of microbubble **chain length** on shell fragment biodistribution in the **A)** liver, **B)** spleen, **C)** heart, **D)** lungs, and **E)** kidneys from PET imaging and end-point γ-counting in 4T1 tumor-bearing animals. Values are presented as the ratio of means ± propagated SD (N=5-6 for all formulations and treatment arms) between C16 chain length microbubbles and C18 chain length microbubbles for each of the 4 groupings: i) anionic, FUS-untreated (light pink); ii) anionic, FUS-treated (pink); iii) uncharged, FUS-untreated (light blue); and iv) uncharged, FUS-treated (blue). Values above the dotted black line indicate greater relative shell fragment accumulation / exposure for shorter chain length microbubbles, while values below the dotted black line indicate reduced relative shell fragment accumulation / exposure for shorter chain length microbubbles. Significant changes are starred, as determined via Wilcoxon Rank Sum tests with Benjamini-Hochberg multi-comparison correct (FDR 0.1, significant p values < (i/m)Q).

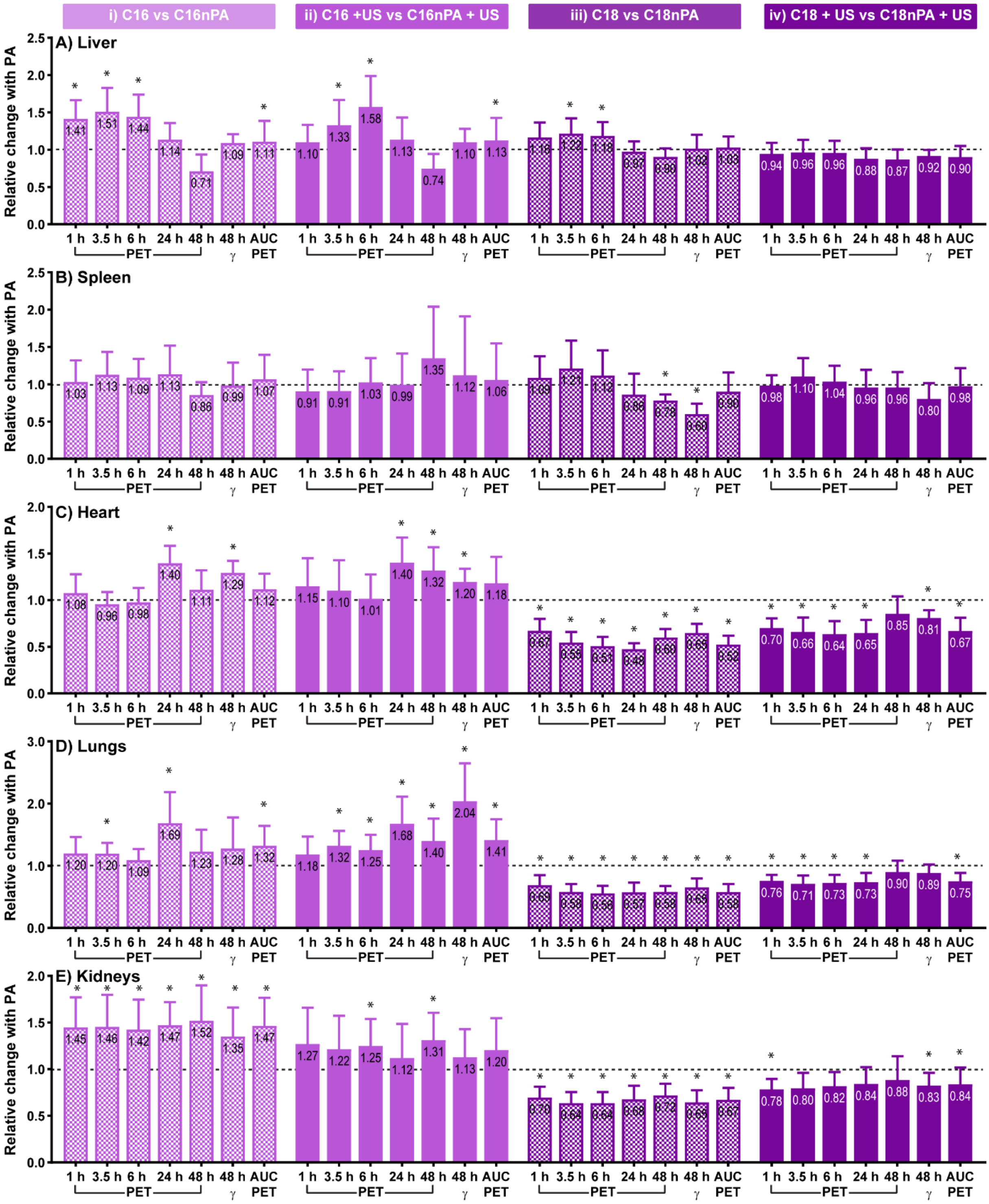


**Figure S22**: Illustrating the impact of **microbubble charge** on shell fragment biodistribution in the **A)** liver, **B)** spleen, **C)** heart, **D)** lungs, and **E)** kidneys from PET imaging and end-point γ-counting in 4T1 tumor-bearing animals. Values are presented as the ratio of means ± propagated SD (N=5-6 for all formulations and treatment arms) between microbubble containing a negatively-charged phosphatidic acid component and those without one (nPA) for each of the 4 groupings: i) C16 chain length, FUS-untreated (light purple, hashed); ii) C16 chain length, FUS-treated (light purple, solid); iii) C18 chain length, FUS-untreated (dark purple, hashed); and iv) C18 chain length, FUS-treated (dark purple solid). Values above the dotted black line indicate greater relative shell fragment accumulation / exposure for negatively charged microbubbles, while values below the dotted black line indicate reduced relative shell fragment accumulation / exposure for negatively charged microbubbles. Significant changes are starred, as determined via Wilcoxon Rank Sum tests with Benjamini-Hochberg multi-comparison correct (FDR 0.1, significant p values < (i/m)Q).

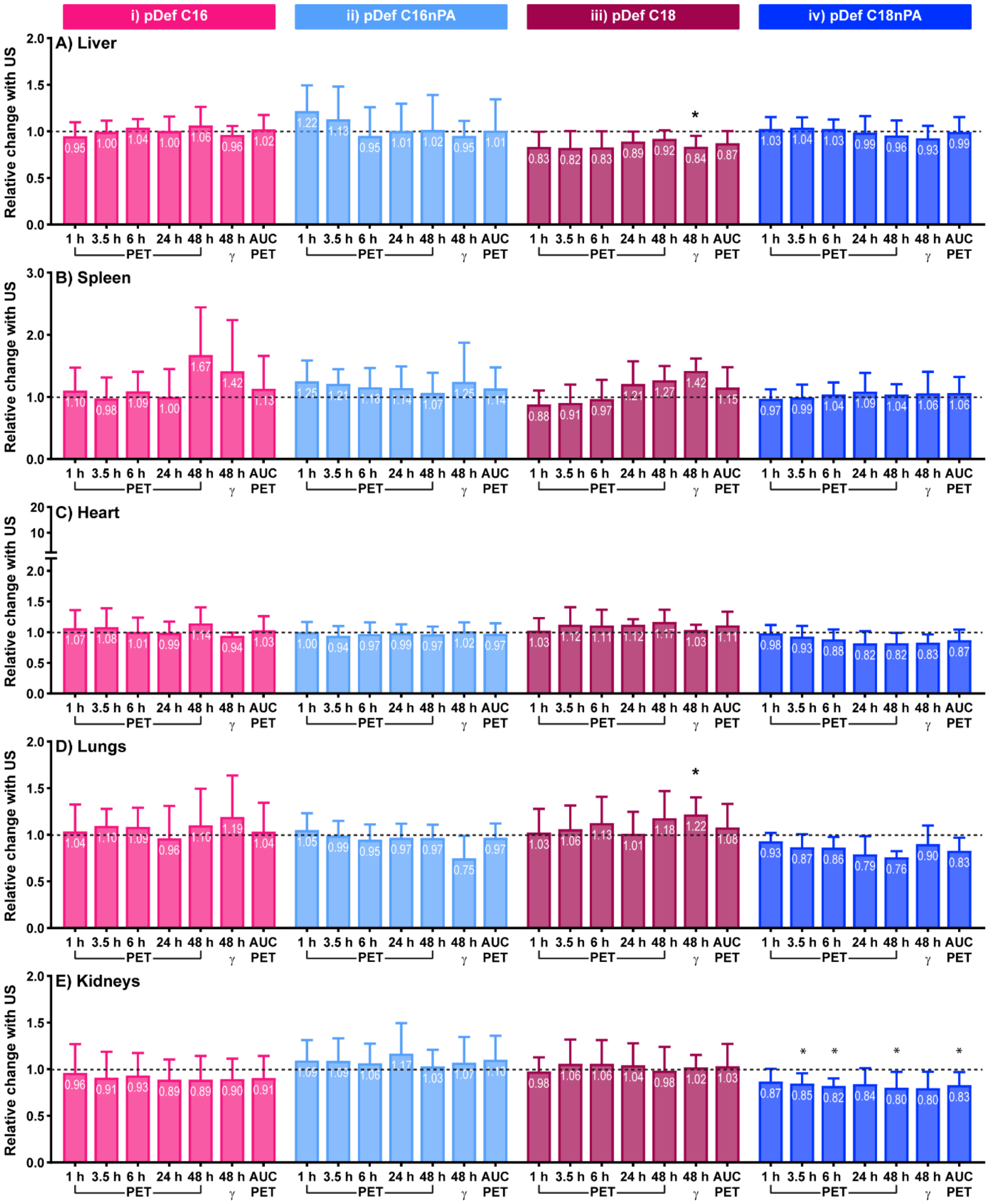


**Figure S23**: Illustrating the impact of **focused ultrasound application** on shell fragment biodistribution in the **A)** liver, **B)** spleen, **C)** heart, **D)** lungs, and **E)** kidneys from PET imaging and end-point γ-counting in 4T1 tumor-bearing animals. Values are presented as the ratio of means ± propagated SD (N=5-6 for all formulations and treatment arms) between animals treated with FUS following microbubble injection microbubble (+US) and those not exposed to FUS at all (-US) for each of the 4 groupings: i) C16 chain length, negative charge (C16: pink); ii) C16 chain length, neutral charge (C16nPA: light blue); iii) C18 chain length, negative charge (C18: dark red); and iv) C18 chain length, neutral charge (C18nPA, dark blue). Values above the dotted black line indicate greater relative shell fragment accumulation / exposure for FUS-treated animals, while values below the dotted black line indicate reduced relative shell fragment accumulation / exposure for FUS-treated animals. Significant changes are starred, as determined via Wilcoxon Rank Sum tests with Benjamini-Hochberg multi-comparison correct (FDR 0.1, significant p values <  (i/m)Q).

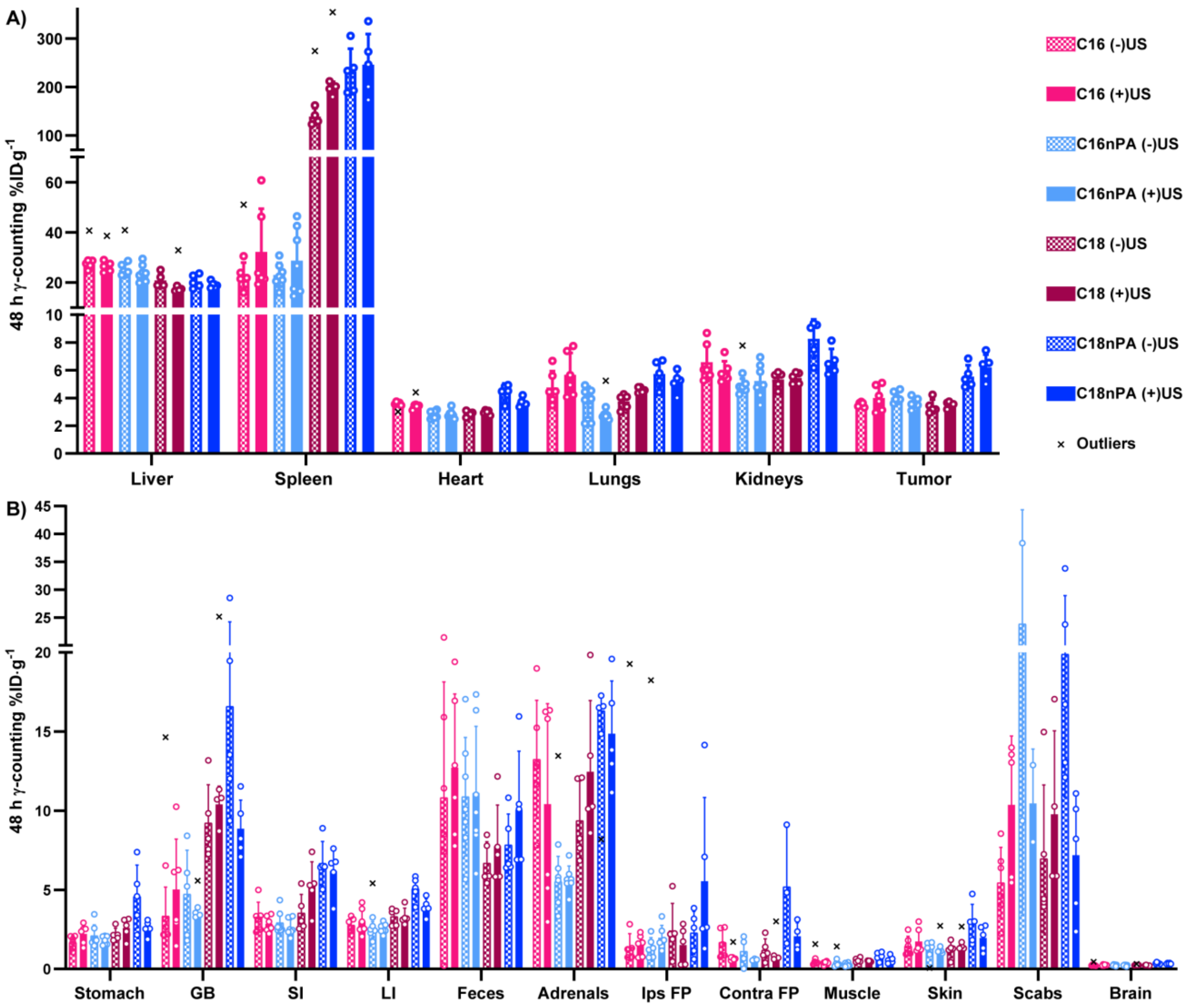


**Figure S24**: 4T1 tumor-bearing BALB/c pDef shell fragment biodistribution assessed by ex vivo tissue γ-counting 48 hours post-injection. Measurements represented as %ID·g$^{-1}$ are shown for **A)** the 6 primary organs of this study and **B)** other important organs and tissues of interest. Outliers identified by Grubb's Test are denoted as 'x's within their respective datasets. GB: intact gall bladder, SI: small intestine, LI: large intestine, Ips FP: ipsilateral inguinal mammary fat pad, Contra FP: contralateral inguinal mammary fat pad, Scabs: portion of skin ulceration overlying 4T1 tumor, if present. N=5-8 for all formulations and experimental arms.

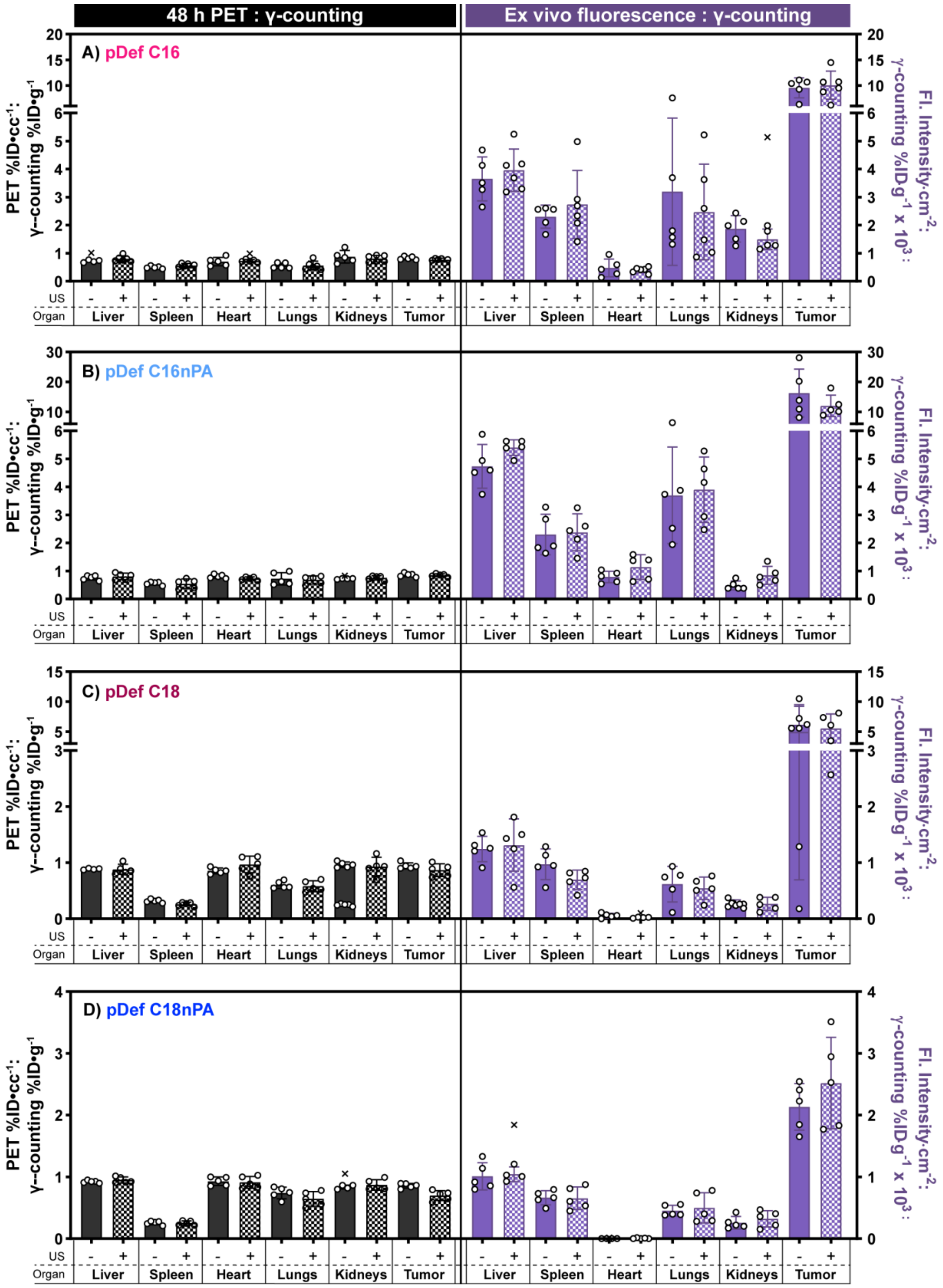


**Figure S25**: Comparison of quantitative PET and semi-qualitative hyperspectral fluorescence imaging-based biodistribution in major organs to ex vivo γ-counting. Values do not represent true ratios given unit differences but provide the consistency between PET and γ-counting (black, left half of graph) across formulations and organs versus fluorescence and γ-counting (purple, right half of graph). Rows correspond to different pDef formulations, including **A)** C16, **B)** C16nPA, **C)** C18, and **D)** C18nPA. PET: γ-counting ratios are consistent across formulations and organs. In contrast, fluorescence cannot be scaled uniformly to γ-counting across organs or formulations, demonstrating that different tumors exhibit different degrees of fluorescence unquenching of pDefs. This asserts the importance of using quantitative means, like PET, to gain true biodistribution data of microbubble shells.

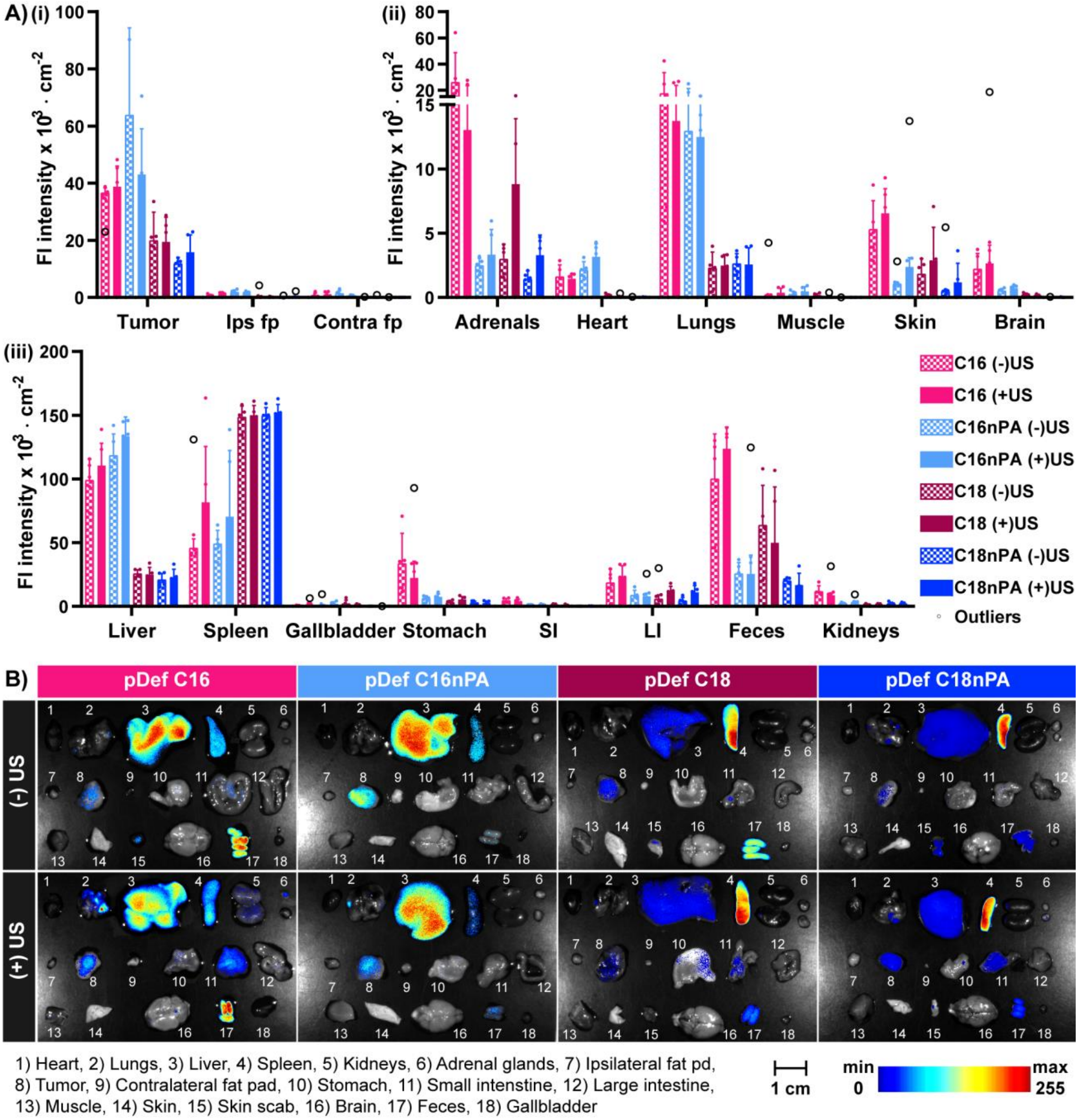


**Figure S26**: Ex vivo hyperspectral fluorescence imaging analysis of organs dissected from 4T1 tumor-bearing mice 48 hours post-injection. **A)** Quantified fluorescence intensity within ROIs corresponding to organs and tissues of interest following autofluorescence removal via spectral unmixing. Outliers are represented by gray circles. All values are displayed as mean ± SD (N=5-6). **B)** Representative ex vivo fluorescence imaging of dissected organs. Spectrally-unmixed fluorescence data is overlaid onto white light images for visualization. Scale bar: 1 cm. Consistent scaling applied to all images.

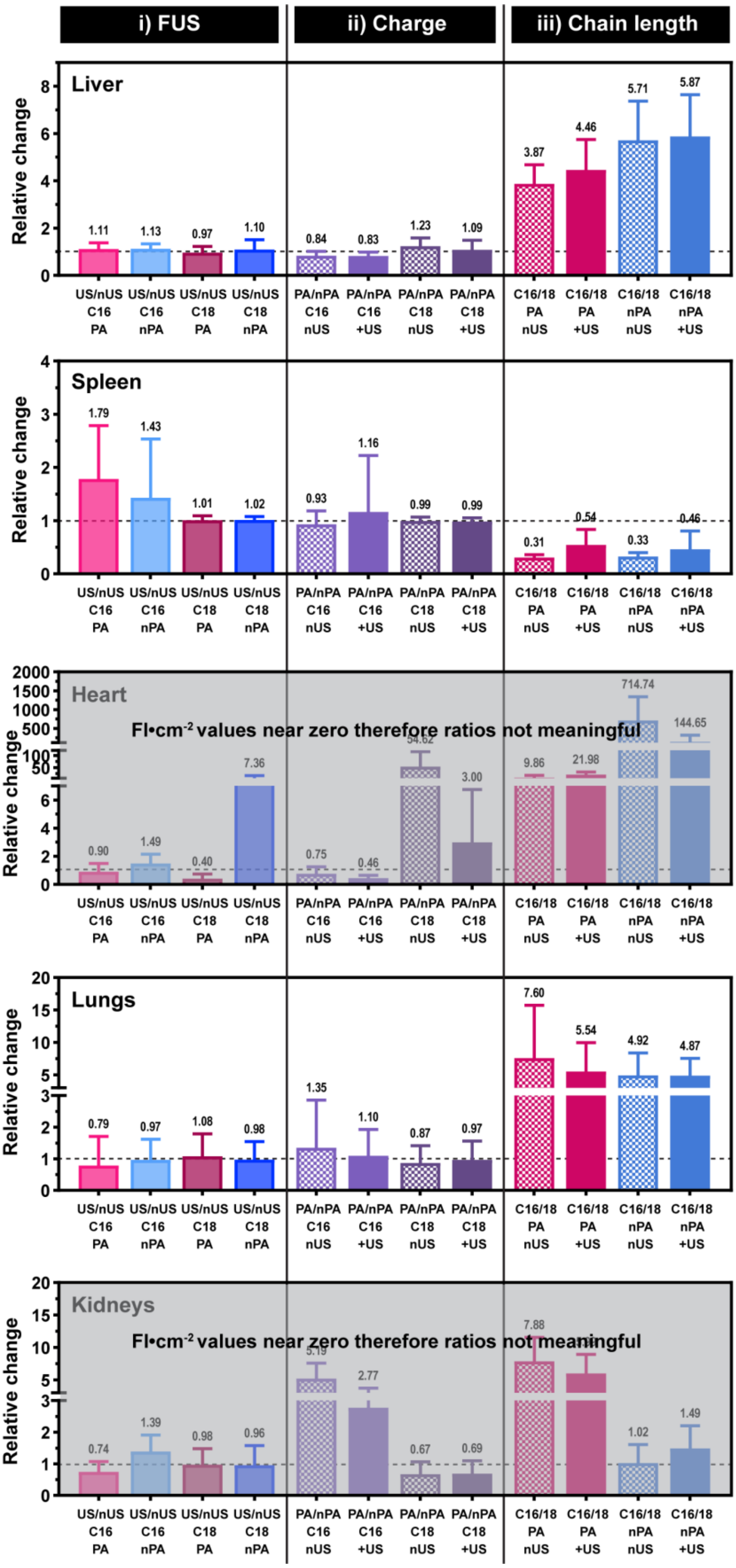


**Figure S27**: Illustrating the impact of **A)** focused ultrasound application, **B)** microbubble charge, and **C)** microbubble chain length on shell fragment **ex vivo fluorescence** of tissues dissected 48 h post-injection. Values represent the ratio of means ± propagated SD (N=5-6 for all formulations and treatment arms) as done in Figures S21-S23. Each row corresponds to a different organ, including liver, spleen, heart, lungs, and kidneys. Ratios calculated for the heart and kidneys are not meaningful in interpretation as fluorescence in these organs was near or at zero. They have accordingly been shaded grey.

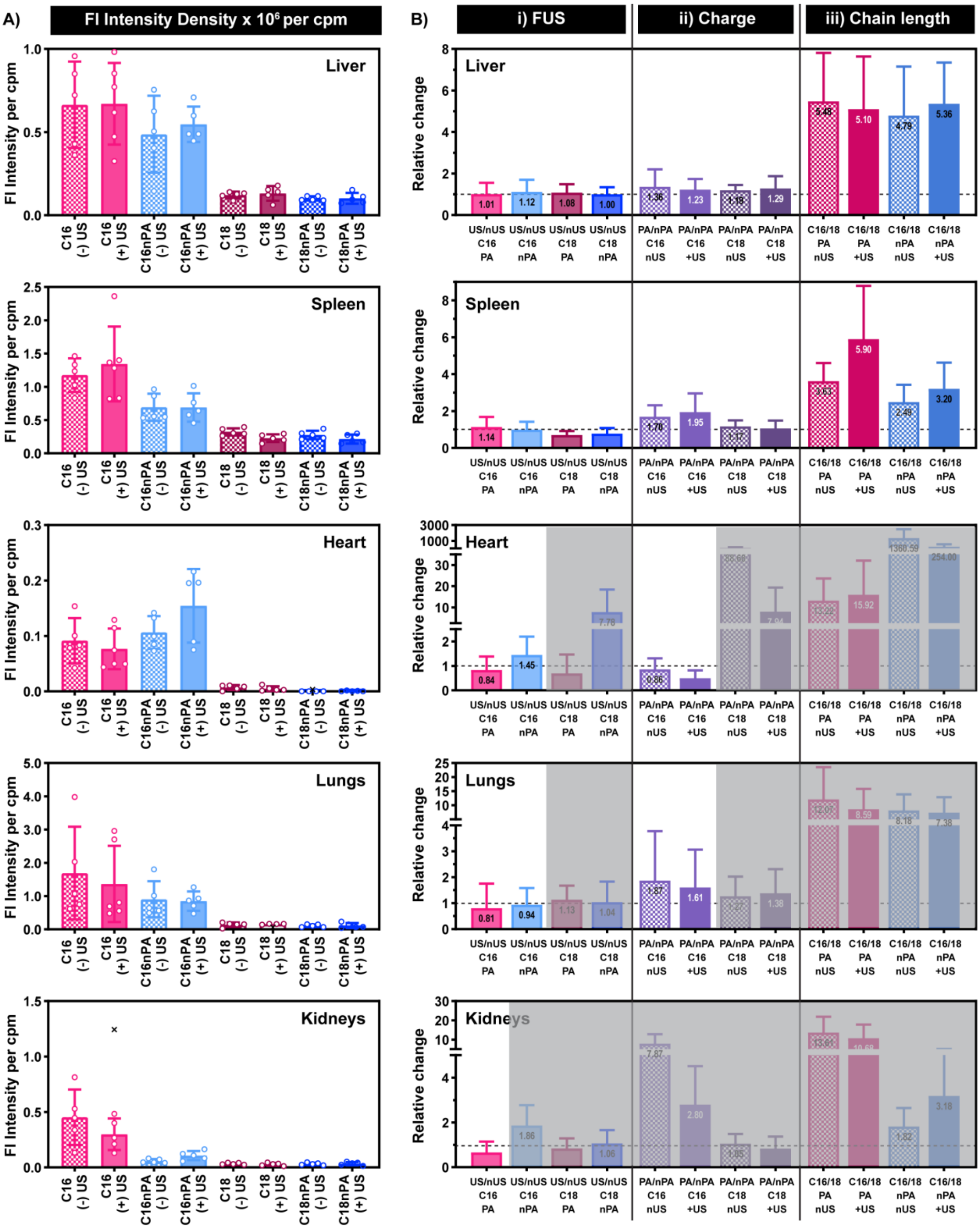


**Figure S28**: **A)** Ex vivo fluorescence intensity normalized by corrected radioactivity (γ-counting) as a proxy for degree of fluorescence unquenching, showcasing how c16 pDefs undergo greater structural dissociation relative to c18 pDefs across the liver, spleen, heart, lungs, and kidneys. **B)** Illustrating the impact of **(i)** focused ultrasound application, **(ii)** microbubble charge, and **(iii)** microbubble chain length on fluorescence unquenching of pDefs. Values are presented as the ratio of means ± propagated SD (N=5-6 for all formulations and treatment arms). Values above the dotted black line indicate greater unquenching given the presence/increase of the stated factor. Some ratios calculated for the heart, lungs and kidneys are not meaningful in interpretation as fluorescence:cpm values in these organs were near zero. They have accordingly been shaded grey.

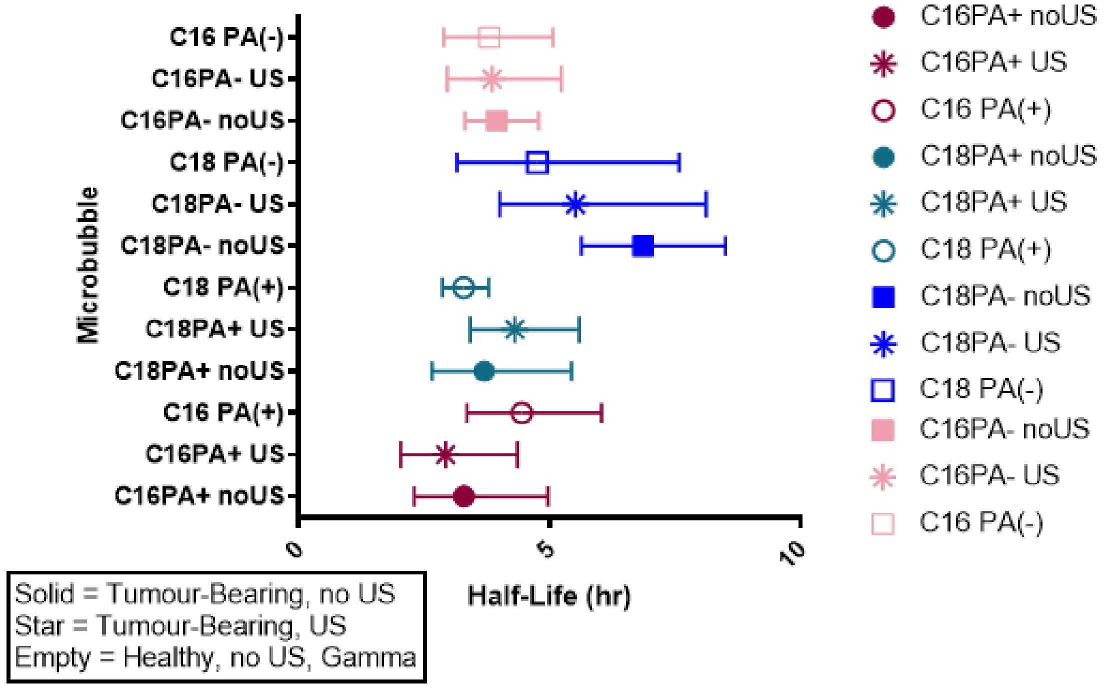


**Figure S29**: Comparison of PET-contoured data for the heart and blood clearance data. Data series were time-matched and modeled using non-linear regression. Formulations were found to not be statistically different from one another when comparing PET and blood clearance data.

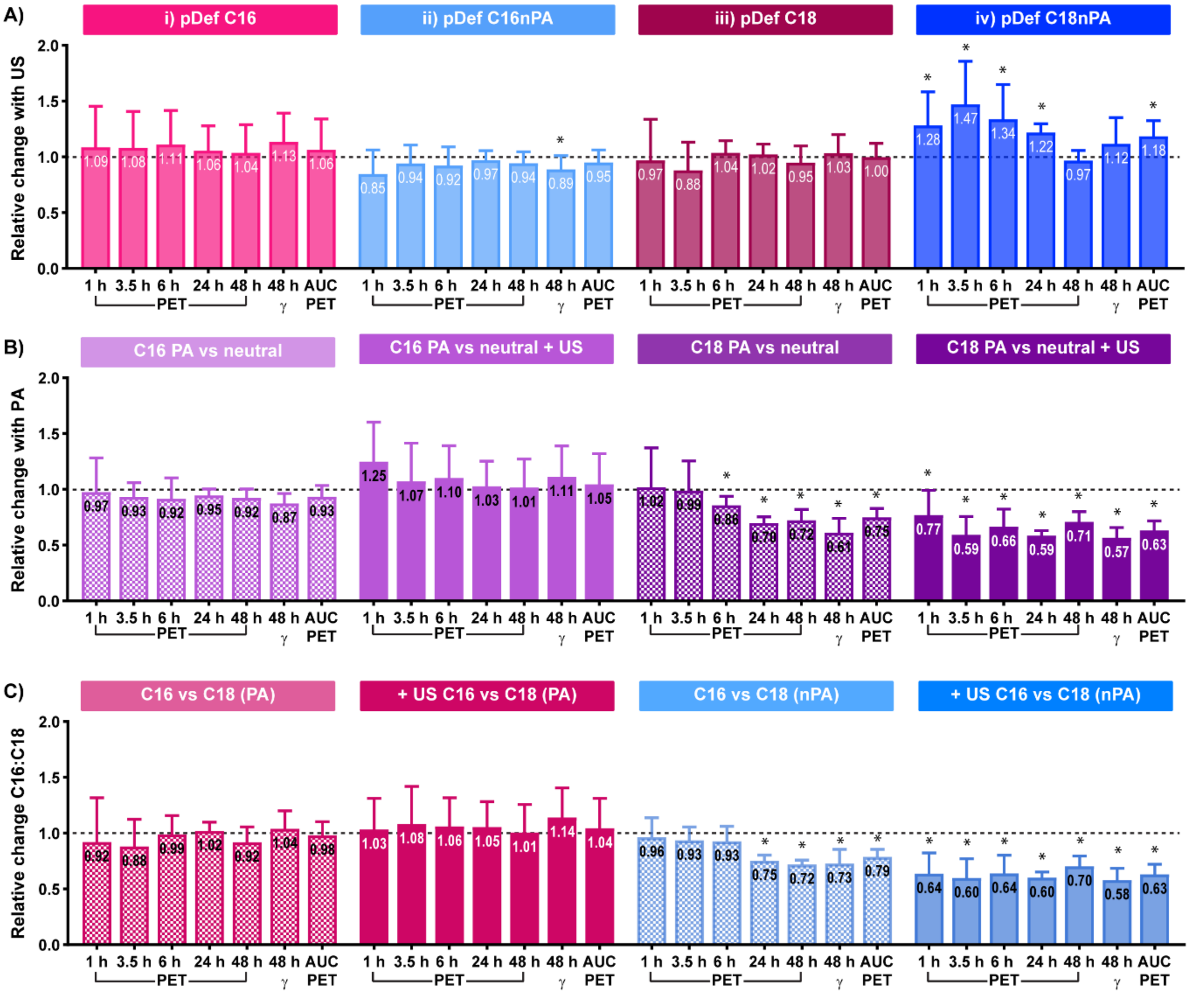


**Figure S30**: Illustrating the impact of **A) focused ultrasound application**, **B) charge, and C) chain length** on shell fragment tumor accumulation from PET imaging and end-point γ-counting in 4T1 tumor-bearing animals. Values are presented as the ratio of means ± propagated SD (N=5-8 for all formulations and treatment arms) between all ratios (FUS/no FUS, charged/uncharged and c16/c18). Values above the dotted black line indicate greater relative shell fragment accumulation / exposure for the associated parameter, while values below the dotted black line indicate reduced relative shell fragment accumulation / exposure for the associated parameter. Significant changes are starred, as determined via one-tailed t-tests (FUS effect, as anticipated change was a one-directional increase) or Wilcoxon Rank Sum tests with Benjamini-Hochberg multi-comparison correct (FDR 0.1, significant p values < (i/m)Q).

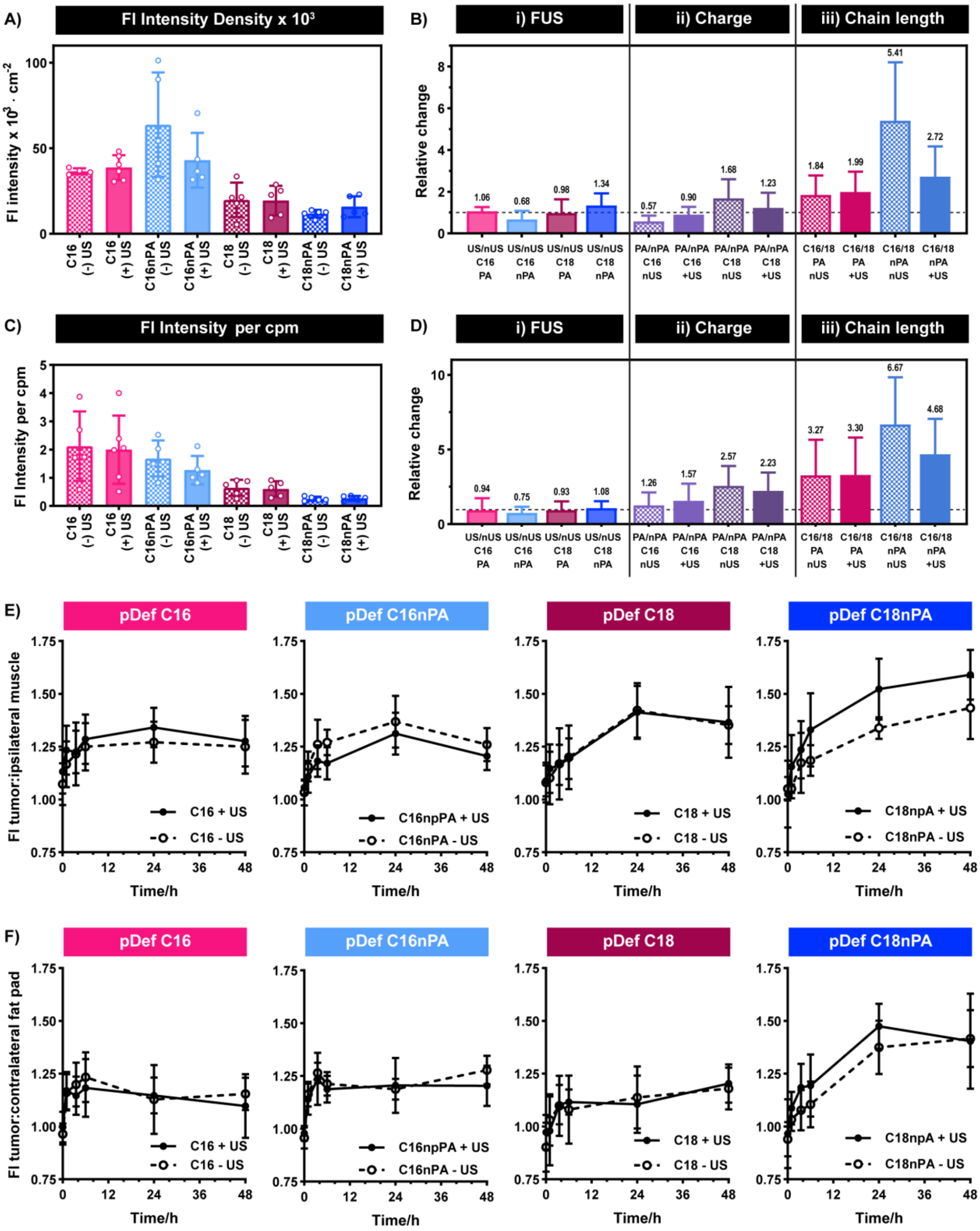


**Figure S31**: Hyperspectral fluorescence imaging signal quantification within 4T1 tumors. **A)** Fluorescence density was quantified by ROI analysis following removal of autofluorescence through spectral unmixing. **B)** The associated impacts of **(i)** focused ultrasound application, **(ii)** charge, and **(iii)** lipid chain length are shown. **C)** Total fluorescence per radioactivity quantified by gamma counting demonstrates relative fluorescence unquenching of pDefs in 4T1 tumors. **D)** The associated impacts of **(i)** focused ultrasound application, **(ii)** charge, and **(iii)** lipid chain length are shown. In vivo ROI analysis of raw hyperspectral fluorescence was conducted against **E)** surrounding ipsilateral muscle and **F)** the contralateral fat pad to demonstrate relative fluorescence signal on versus off-target. Values are presented as the mean or ratio of means ± SD (N=5-6) for all formulations and treatment arms. Values above the dotted black line indicate greater relative fluorescence for the associated parameter, while values below the dotted black line indicate reduced relative fluorescence.

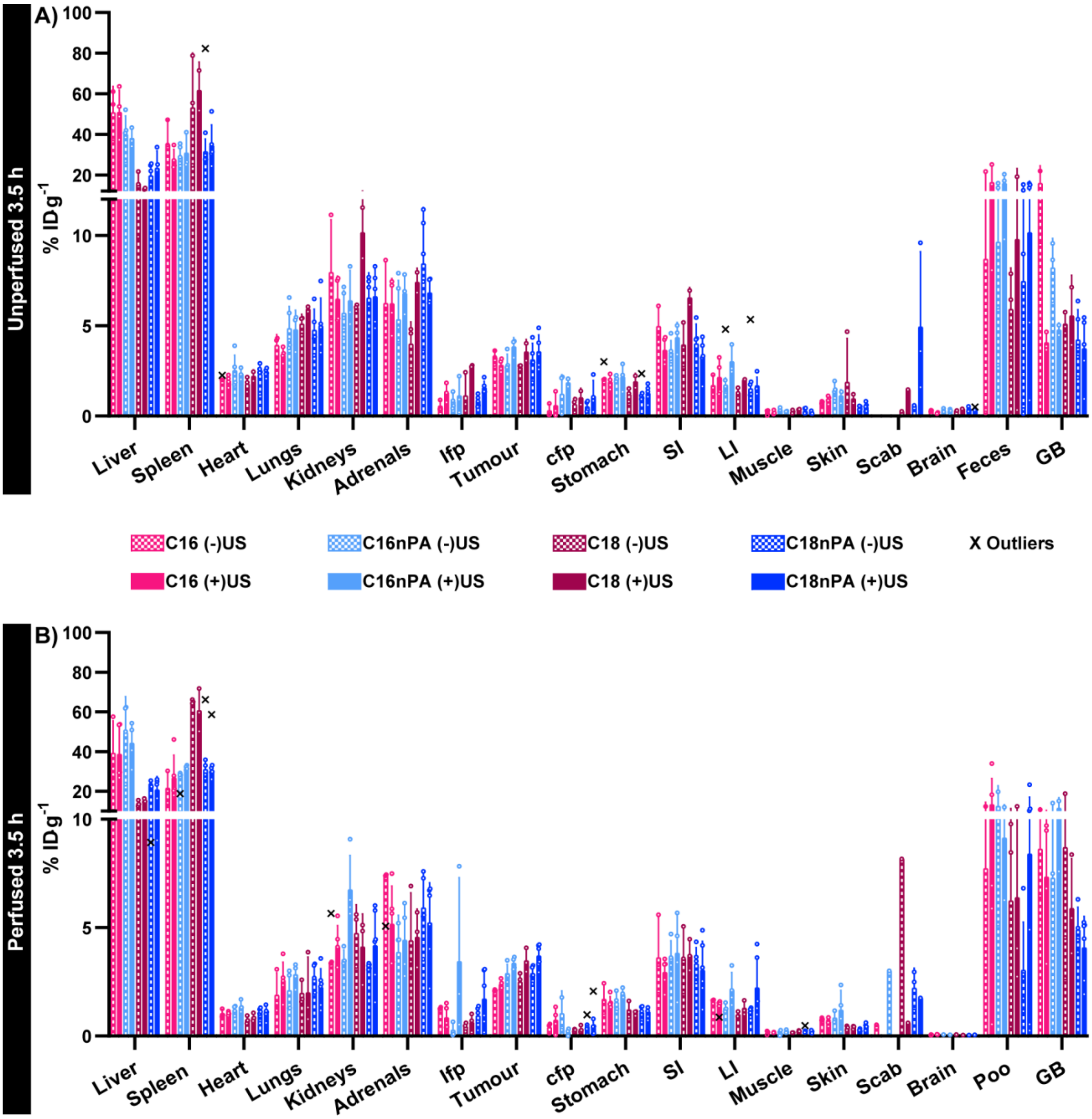


**Figure S32**: 4T1 tumor-bearing BALB/c pDef shell fragment biodistribution assessed by ex vivo tissue γ-counting 3.5 hours post-injection for **A)** unperfused and **B)** perfused animals. Outliers identified by Grubb's Test are denoted as 'x's within their respective datasets. GB: intact gall bladder, SI: small intestine, LI: large intestine, Ips FP: ipsilateral inguinal mammary fat pad, Contra FP: contralateral inguinal mammary fat pad, Scabs: portion of skin ulceration overlying 4T1 tumor, if present. N=5-8 for all formulations and experimental arms.

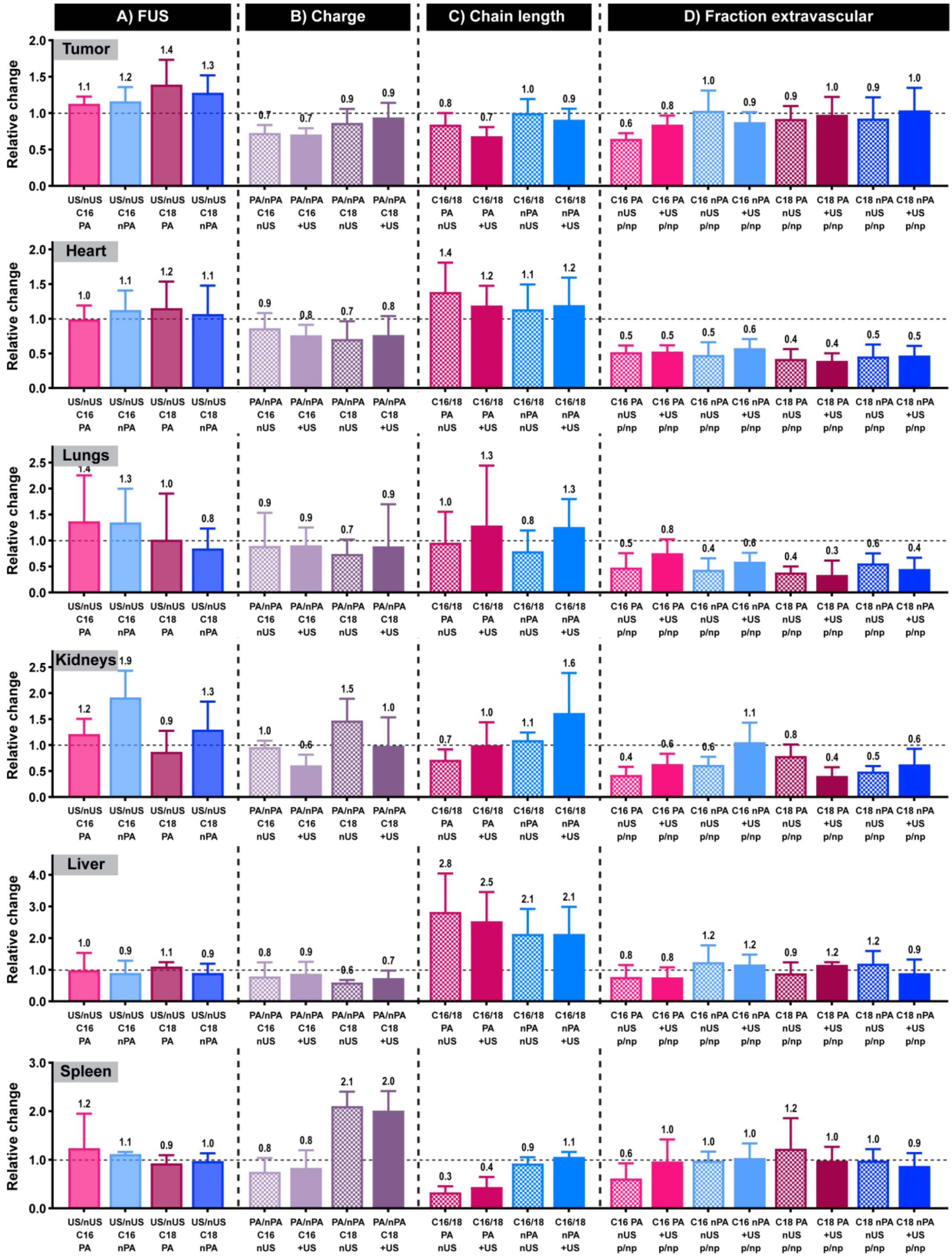


**Figure S33**: Impact of focused ultrasound (**A**; FUS/no FUS), microbubble charge (**B**; charged/uncharged) and microbubble lipid chain length (**C**; c16/c18) on tumor, heart, lung, kidney, liver and spleen uptake of pDef shells, presented as ratios of %ID·g$^{-1}$ obtained from gamma counting of organs from perfused 4T1 tumor-bearing animals. **D)** Ratio of %ID·g$^{-1}$ for perfused/unperfused organs provides a proxy for the fraction of shell accumulation that is extravascular in nature (inclusive of endothelial uptake). Values are presented as the ratio of means ± propagated SD (N=5-8 for all formulations and treatment arms). Values above the dotted black line indicate greater relative shell fragment accumulation / exposure for the associated parameter, while values below the dotted black line indicate reduced relative shell fragment accumulation / exposure for the associated parameter.

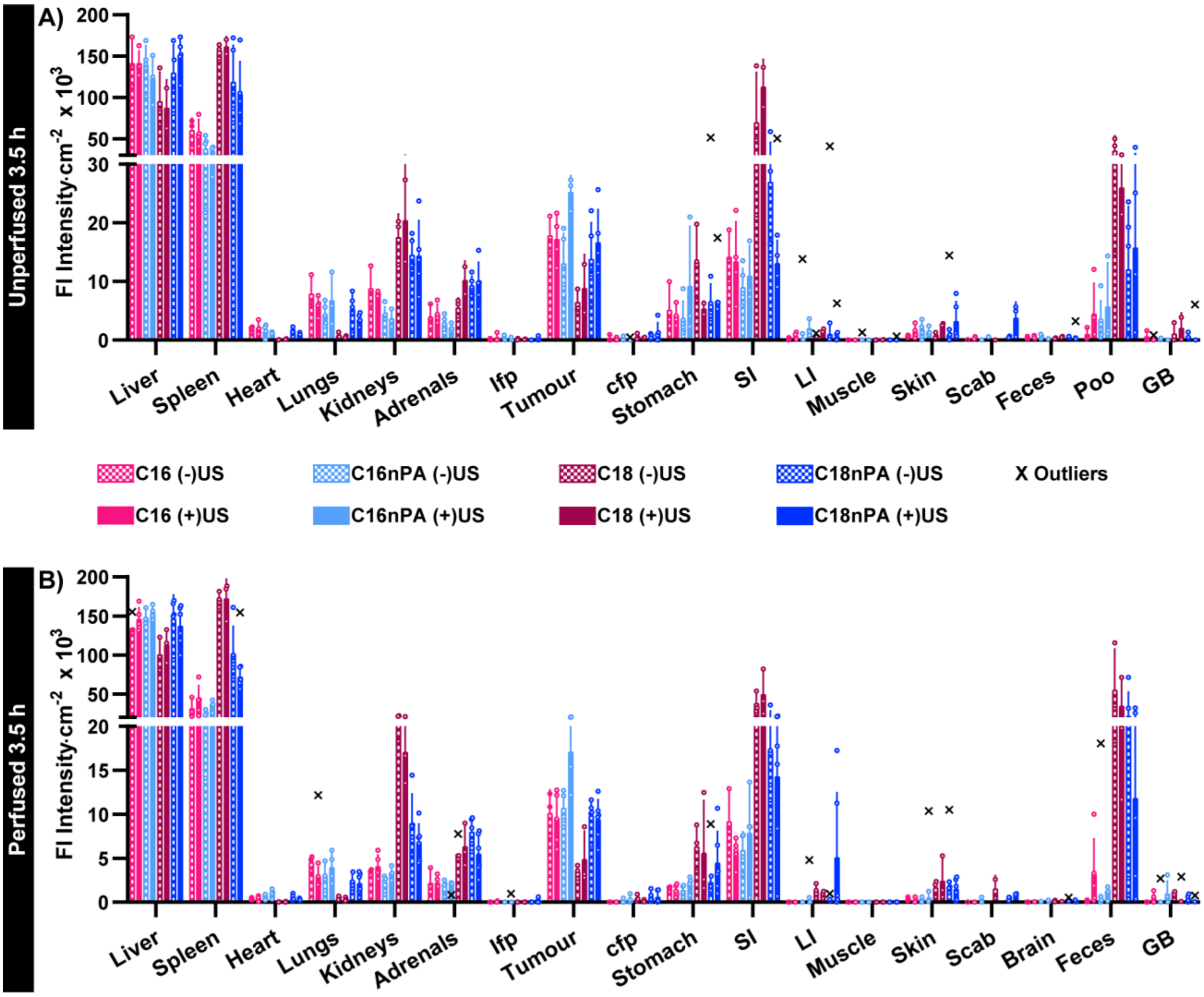


**Figure S34**: 4T1 tumor-bearing BALB/c ex vivo tissue hyperspectral fluorescence imaging ROI signals 3.5 hours post-injection for **A)** unperfused and **B)** perfused animals. Outliers identified by Grubb's Test are denoted as 'x's within their respective datasets. GB: intact gall bladder, SI: small intestine, LI: large intestine, Ips FP: ipsilateral inguinal mammary fat pad, Contra FP: contralateral inguinal mammary fat pad, Scabs: portion of skin ulceration overlying 4T1 tumor, if present. N=5-8 for all formulations and experimental arms.

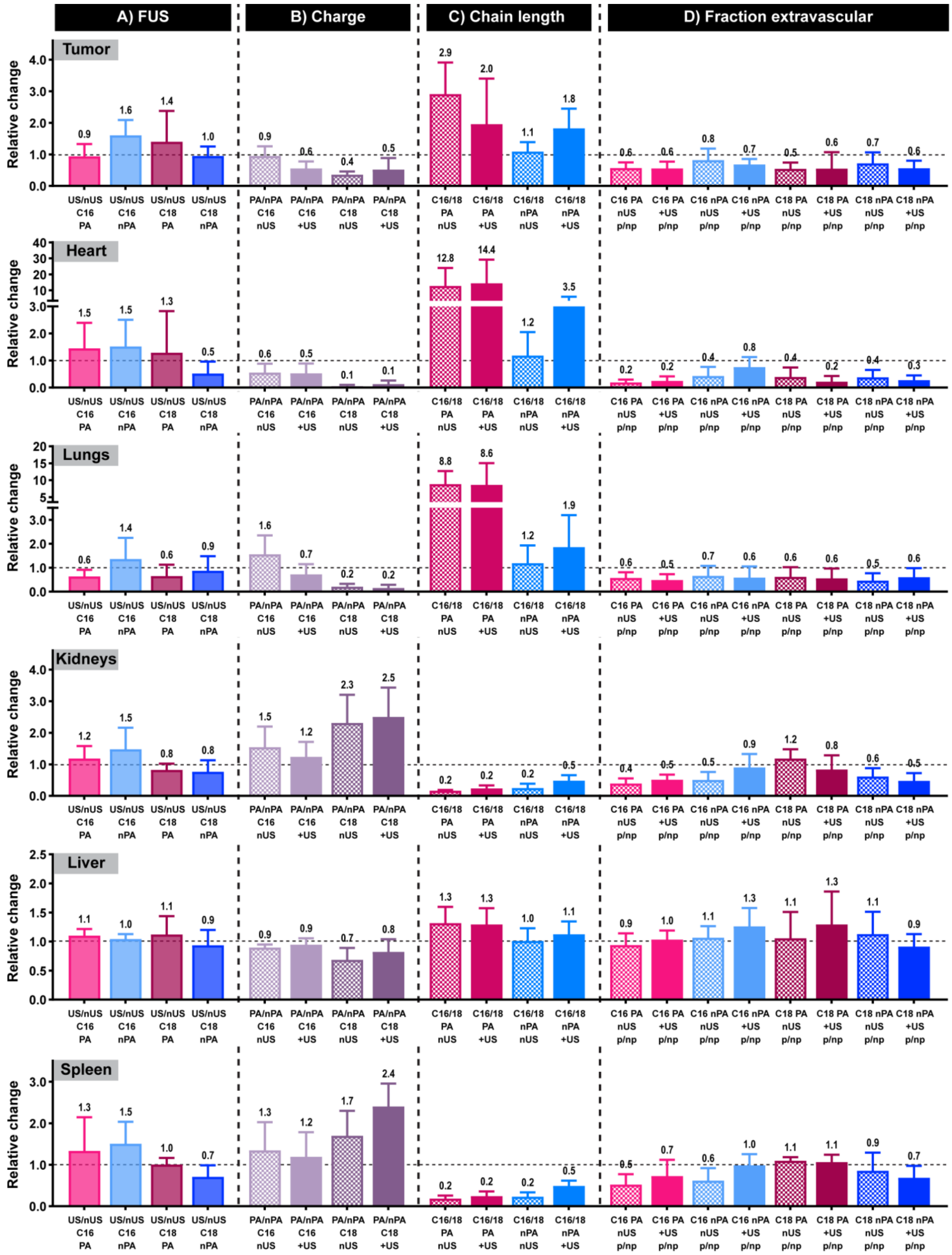


**Figure S35**: Impact of focused ultrasound (**A**; FUS/no FUS), microbubble charge **(B**; charged/uncharged) and microbubble lipid chain length (**C**; c16/c18) on tumor, heart, lung, kidney, liver and spleen fluorescence signal obtained from ROIs drawn manually in ImageJ and summarized in Figure S34. **D)** Ratio of fluorescence signal·$cm^{-2}$ for perfused/unperfused organs provides a proxy for the fraction of shell signal that is extravascular in nature (inclusive of endothelial uptake). Values are presented as the ratio of means ± propagated SD (N=5-8 for all formulations and treatment arms). Values above the dotted black line indicate greater relative shell fragment accumulation / exposure for the associated parameter, while values below the dotted black line indicate reduced relative shell fragment accumulation / exposure for the associated parameter.

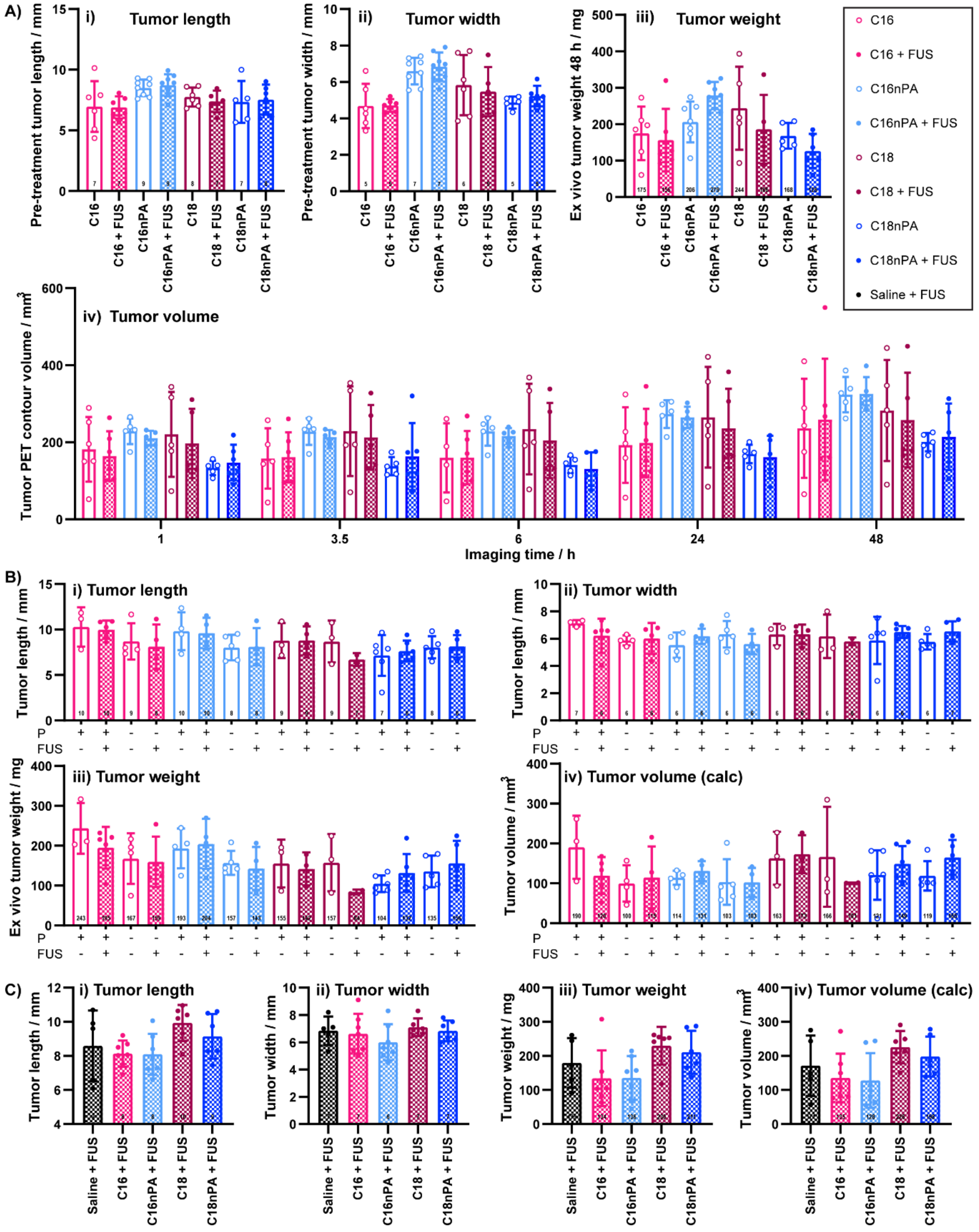


**Figure S36**: 4T1 tumor sizes for **A)** PET/CT kinetic biodistribution, **B)** 3.5 h perfusion and **C)** Evans Blue studies. Tumor length (**i**) and width (**ii**) were measured with calipers, on the basis of which animals were divided into the summarized cohorts. Tumor weights were measured following excision at 48 hours post treatment for PET/CT cohorts (**A, iii**), and 3.5 hours post treatment for both perfusion (**B, iii**) and Evans Blue studies (**C, iii**). Tumor volumes were empirically measured via PET/CT for kinetic biodistribution studies (**A, iv**) and were estimated via ellipsoid volume calculations for perfusion (**B, iv**) and Evans Blue (**C, iv**) cohorts based on caliper measurements. All data is presented as an average ± standard deviation for each experimental arm.

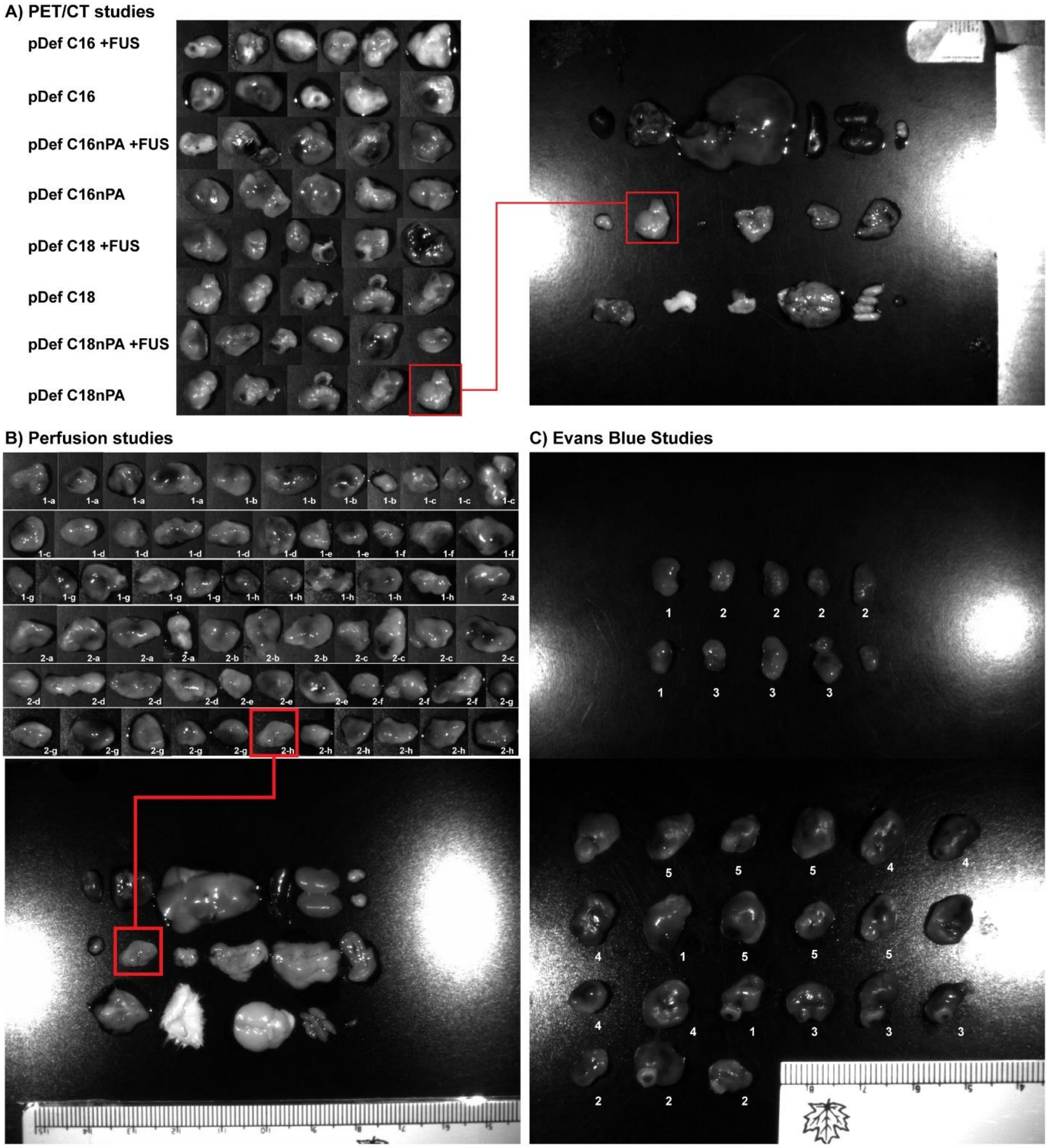


**Figure S37**: White light images of excised 4T1 tumors for **A)** PET/CT kinetic biodistribution, **B)** 3.5 h perfusion and **C)** Evans Blue studies taken using a CRI Maestro® imaging system. For **A)** and **B)**, all tumors were imaged separately alongside other excised organs, and an example of the full plate view (including ruler scale bar) is provided (outlined in red) for one of the mice. Lines in each of the ruler scale bars represent 1 mm intervals. In **B)**, tumors are labeled as 1-unperfused, 2-perfused, and letters represent different microbubble/FUS treatments: a) C16 pDef + FUS, b) C16 pDef, c) C16nPA pDef + FUS, d) C16nPA pDef, e) C18 pDef + FUS, f) C18 pDef, g) C18nPA pDef + FUS, and h) C18nPA pDef. In **C)**, numbers represent the following treatment groups: 1-saline+FUS control, 2-C16 pDef + FUS, 3-C16nPA pDef + FUS, 4-C18 pDef + FUS, and 5-C18nPA pDef+FUS.

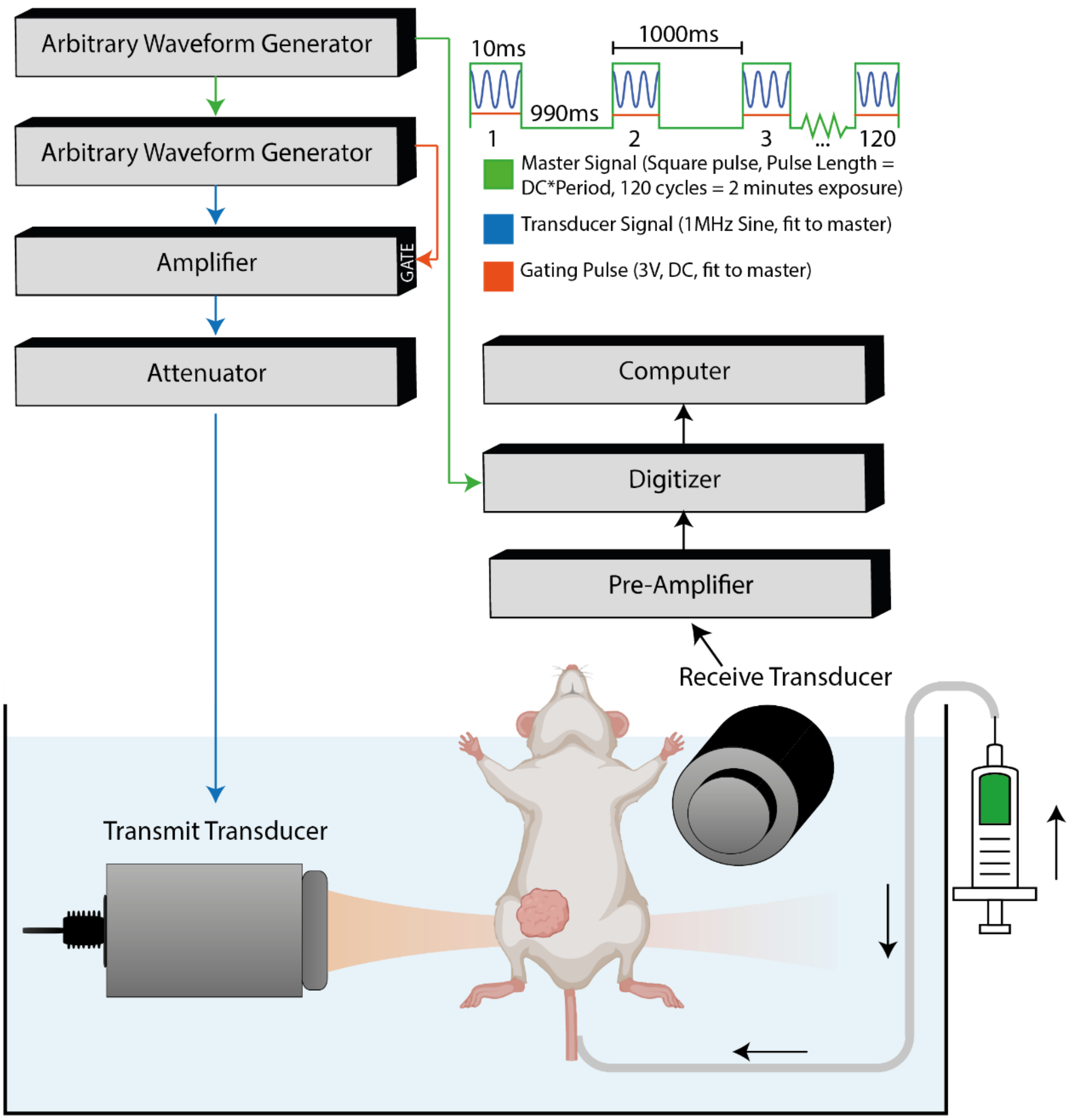


**Figure S368**: Schematic of the components of the custom focused ultrasound delivery system constructed for this work. The orientation pictured was used for in vivo tumor FUS exposures.

References

1. Lovell, J. F.; Jin, C. S.; Huynh, E.; Jin, H.; Kim, C.; Rubinstein, J. L.; Chan, W. C. W.; Cao, W.; Wang, L. V.; Zheng, G., Porphysome nanovesicles generated by porphyrin bilayers for use as multimodal biophotonic contrast agents. *Nature Materials* **2011,** *10* (4), 324-332.
2. FDA, Definity(R) (Perflutren Lipid Microsphere) Injectable Suspension. 2001.
3. Huynh, E.; Jin, C. S.; Wilson, B. C.; Zheng, G., Aggregate enhanced trimodal porphyrin shell microbubbles for ultrasound, photoacoustic, and fluorescence imaging. *Bioconjug. Chem.* **2014,** *25* (4), 796-801.
4. Liu, T. W.; MacDonald, T. D.; Shi, J.; Wilson, B. C.; Zheng, G., Intrinsically copper-64-labeled organic nanoparticles as radiotracers. *Angew. Chem. Int. Ed. Engl.* **2012,** *51* (52), 13128-31.
5. Overchuk, M.; Harmatys, K. M.; Sindhwani, S.; Rajora, M. A.; Koebel, A.; Charron, D. M.; Syed, A. M.; Chen, J.; Pomper, M. G.; Wilson, B. C.; Chan, W. C. W.; Zheng, G., Subtherapeutic Photodynamic Treatment Facilitates Tumor Nanomedicine Delivery and Overcomes Desmoplasia. *Nano Lett.* **2021,** *21* (1), 344-352.
6. Cheng, M. H. Y.; Overchuk, M.; Rajora, M. A.; Lou, J. W. H.; Chen, Y.; Pomper, M. G.; Chen, J.; Zheng, G., Targeted Theranostic 111In/Lu-Nanotexaphyrin for SPECT Imaging and Photodynamic Therapy. *Mol. Pharm.* **2022,** *19* (6), 1803-1813.
7. Rajora, M. A.; Ding, L.; Valic, M.; Jiang, W.; Overchuk, M.; Chen, J.; Zheng, G., Tailored theranostic apolipoprotein E3 porphyrin-lipid nanoparticles target glioblastoma. *Chem. Sci.* **2017,** *8* (8), 5371-5384.
8. Olson, B. J.; Markwell, J., Assays for determination of protein concentration. *Current protocols in protein science / editorial board, John E. Coligan ... [et al.]* **2007,** *Chapter 3*.
9. Pulaski, B. A.; Ostrand-Rosenberg, S., Mouse 4T1 breast tumor model. *Current protocols in immunology / edited by John E. Coligan ... [et al.]* **2001,** *Chapter 20*.
10. Greis, C., Quantitative evaluation of microvascular blood flow by contrast-enhanced ultrasound (CEUS). *Clin. Hemorheol. Microcirc.* **2011**.
11. Strouthos, C.; Lampaskis, M.; Sboros, V.; McNeilly, A.; Averkiou, M., Indicator dilution models for the quantification of microvascular blood flow with bolus administration of ultrasound contrast agents. *IEEE Transactions on Ultrasonics, Ferroelectrics, and Frequency Control* **2010**.
12. Madsen, M. T., A simplified formulation of the gamma variate function. *Phys. Med. Biol.* **1992,** *37* (7), 1597-1600.
13. Pellow, C.; Acconcia, C.; Zheng, G.; Goertz, D. E., Threshold-dependent nonlinear scattering from porphyrin nanobubbles for vascular and extravascular applications. *Phys. Med. Biol.* **2018,** *63* (21).
14. Pellow, C.; O'Reilly, M. A.; Hynynen, K.; Zheng, G.; Goertz, D. E., Simultaneous Intravital Optical and Acoustic Monitoring of Ultrasound-Triggered Nanobubble Generation and Extravasation. *Nano Lett.* **2020,** *20* (6), 4512-4519.
15. Huynh, E.; Leung, B. Y.; Helfield, B. L.; Shakiba, M.; Gandier, J. A.; Jin, C. S.; Master, E. R.; Wilson, B. C.; Goertz, D. E.; Zheng, G., In situ conversion of porphyrin microbubbles to nanoparticles for multimodality imaging. *Nat Nanotechnol* **2015,** *10* (4), 325-32.
16. Arvanitis, C. D.; Askoxylakis, V.; Guo, Y.; Datta, M.; Kloepper, J.; Ferraro, G. B.; Bernabeu, M. O.; Fukumura, D.; McDannold, N.; Jain, R. K., Mechanisms of enhanced drug delivery in brain metastases with focused ultrasound-induced blood-tumor barrier disruption. *Proc. Natl. Acad. Sci. U. S. A.* **2018,** *115* (37), E8717-E8726.
17. Yan, F.; Li, L.; Deng, Z.; Jin, Q.; Chen, J.; Yang, W.; Yeh, C. K.; Wu, J.; Shandas, R.; Liu, X.; Zheng, H., Paclitaxel-liposome-microbubble complexes as ultrasound-triggered therapeutic drug delivery carriers. *J. Control. Release* **2013,** *166* (3), 246-255.

18. Gratton, S. E. A.; Pohlhaus, P. D.; Lee, J.; Guo, J.; Cho, M. J.; DeSimone, J. M., Nanofabricated particles for engineered drug therapies: A preliminary biodistribution study of PRINT™ nanoparticles. *J. Control. Release* **2007,** *121* (1-2), 10-18.
19. Dhaliwal, A.; Ma, J.; Zheng, M.; Rajora, M. A.; Lyu, Q.; Ma, S.; Oliva, L.; Valic, M.; Zheng, B.; Wang, B., Deep Learning for Nanomedicine Auto-Segmentation and Pharmacokinetics. *Nat Commun* **2023,** *In submission*.
